# Analytical Techniques for Phytocannabinoid Profiling of Cannabis and Cannabis-Based Products—A Comprehensive Review

**DOI:** 10.3390/molecules27030975

**Published:** 2022-02-01

**Authors:** Gjoshe Stefkov, Ivana Cvetkovikj Karanfilova, Veronika Stoilkovska Gjorgievska, Ana Trajkovska, Nikola Geskovski, Marija Karapandzova, Svetlana Kulevanova

**Affiliations:** 1 Institute of Pharmacognosy, Faculty of Pharmacy, Ss. Cyril and Methodius University, Bul. Majka Tereza 47, 1000 Skopje, North Macedonia; gost@ff.ukim.edu.mk (G.S.); vsgjorgievska@ff.ukim.edu.mk (V.S.G.); ana.trajkovska@ff.ukim.edu.mk (A.T.); Marija_Karapandzova@ff.ukim.edu.mk (M.K.); svku@ff.ukim.edu.mk (S.K.); 2 Institute of Pharmaceutical Technology, Faculty of Pharmacy, Ss. Cyril and Methodius University, Bul. Majka Tereza 47, 1000 Skopje, North Macedonia; ngeskovski@ff.ukim.edu.mk

**Keywords:** *Cannabis sativa*, sample preparation, analysis, quality control

## Abstract

Cannabis is gaining increasing attention due to the high pharmacological potential and updated legislation authorizing multiple uses. The development of time- and cost-efficient analytical methods is of crucial importance for phytocannabinoid profiling. This review aims to capture the versatility of analytical methods for phytocannabinoid profiling of cannabis and cannabis-based products in the past four decades (1980–2021). The thorough overview of more than 220 scientific papers reporting different analytical techniques for phytocannabinoid profiling points out their respective advantages and drawbacks in terms of their complexity, duration, selectivity, sensitivity and robustness for their specific application, along with the most widely used sample preparation strategies. In particular, chromatographic and spectroscopic methods, are presented and discussed. Acquired knowledge of phytocannabinoid profile became extremely relevant and further enhanced chemotaxonomic classification, cultivation set-ups examination, association of medical and adverse health effects with potency and/or interplay of certain phytocannabinoids and other active constituents, quality control (QC), and stability studies, as well as development and harmonization of global quality standards. Further improvement in phytocannabinoid profiling should be focused on untargeted analysis using orthogonal analytical methods, which, joined with cheminformatics approaches for compound identification and MSLs, would lead to the identification of a multitude of new phytocannabinoids.

## 1. Introduction

*Cannabis sativa* L. (*C. sativa* L.)*,* from the family Cannabaceae, is the most widely cultivated, trafficked, consumed and investigated, yet most notorious and controversial, plant in the world [1,2]. It is one of the oldest known crops to humanity, with first records of use dating to 3000 B.C. [3], and one of the most commonly used plants for industrial and medical purposes, with a global legal market expected to reach 147 billion USD by the end of 2027 but also the world’s most widespread drug of abuse [1], comprising around 200 million global users.

### 1.1. Botany of C. sativa

*C. sativa* is an annual dioecious plant with histaminate male and pistillate female flowers on separate plants. It grows up to 5 m height, with serrated leaves with a distinct vein pattern that extends to their tips [4]. The inflorescences of the female plants produce several individual bunches of flowers, a large cluster on the upper torso and various small clusters in each branch, covered by trichome glands containing resin rich in phytocannabinoids and terpenoids. Phytocannabinoids are mainly accumulated in the glands of both capitate stalked and capitate-sessile trichomes, but mostly in the latter [4]. *C. sativa* was first classified in 1753 by the Swedish botanist Carolus Linnaeus (Carl Von Linné). More than 2 centuries later, despite its wide use, *C. sativa* is considered a plant with inconclusive taxonomic organization and evolutionary history that are the subject of constant scientific debates [3,5,6]. The United Nations Office on Drugs and Crime (UNODC) [5] considers that the plant has only one recognized species, *C. sativa* L. [5,6,7], although other reported taxa for this genus, such as *C. sativa* subsp. *sativa*, *C. sativa* subsp. *indica*, *C. sativa* subsp. *ruderalis*, *C. sativa* subsp. *spontanea*, and *C. sativa* subsp. *kafiristanca* are currently recognized as subspecies [3,5,6,7]. Today, due to the difficulty in distinguishing cannabis species either morphologically or chemically, and given the continuous changes occurring in subspecies according to the cultivation environment, the designation *C. sativa* is considered suitable for all plants from the genus [3,5]. 

### 1.2. Phytocannabinoids

A wide variety of chemical constituents, i.e., more than 750 compounds, have been identified in *C. sativa*, belonging to the various classes of natural products such as mono- and sesquiterpenes, flavonoids, steroidsand nitrogen-containing compounds [8]. Among them, more than 100 are classified as phytocannabinoids [9], the family of plant-derived C_21_ or C_22_ terpenophenolic compounds, including analogues and metabolites. They are synthesized in secreting cells of glandular trichomes in a biosynthetic pathway from geranyl pyrophosphate (GPP) as the parent precursor of both phytocannabinoids and terpenes. By coupling with olivetolic acid or divarinic acid, C_5_ or C_3_ cannabinoid acids are produced, respectively [10]. Most phytocannabinoids naturally occur as acidic precursors in unfertilized female flowers prior to senescence [2], of which delta-9-tetrahydrocannabinolic acid (Δ^9^-THCA), cannabidiolic acid (CBDA), cannabigerolic acid (CBGA) and cannabichromenic acid (CBCA) are most abundant, with cannabidivarinic acid (CBDVA) and tetrahydrocannabivarinic acid (THCVA) as less abundant [11]. Lower phytocannabinoids content is found in leavesand stems, while absent from roots and seeds.

**Figure 1:**
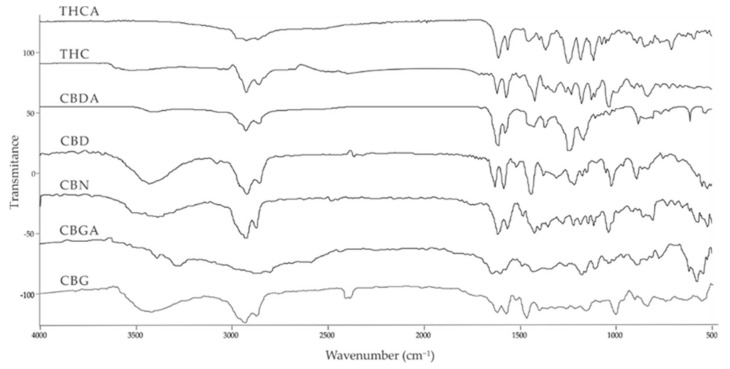
Mid−IR spectra from the main cannabinoids; THCA, THC, CBDA, CBD were adapted from [21,22]; CBN, CBGA and CBG were adapted from [18].

Cannabinoid acids are converted to their neutral counterparts by decarboxylation induced by heat or age. Cannabidiol (CBD), the first cannabinoid was isolated from *C. sativa* in 1963 [12], -delta-9--tetrahydrocannabinol (Δ^9^-THC), the second cannabinoid from *C. sativa* in 1964 [13], delta-8-tetrahydrocannabinol (Δ^8^-THC), the third, cannabigerol (CBG), isolated in 1964, followed by cannabichromene (CBC), isolated in 1966 [14], cannabidivarin (CBDV) [15] and tetrahydrocannabivarin (THCV) [16], which are formed from CBDA, Δ^9^-THCA, CBGA, CBCA, CBDVA and THCVA, respectively [17]. Oxidative degradation of Δ^9^-THC results in the formation of cannabinol (CBN), while isomerization leads to the formation of the more stable, but less active isomer of Δ^9^-THC, Δ^8^-THC. Δ^9^-THCA can degrade to cannabinolic acid (CBNA) and further to CBN. Molecular and structural formula, molecular mass and major fragments as well as UV-VIS spectrum and mid-IR spectra of major phytocannabinoids are presented in Table 1 and Figure 1.

### 1.3. Use of C. sativa

For more than 12,000 years, *Cannabis* spp. is used as a source of textile fiber and food worldwide [23]. The earliest data for medical use of *Cannabis* by the Assyrians goes back to 3000 B.C. Follow-up records date from around 2700 B.C. in China, where *C. sativa* was used as a medicine for menstrual fatigue, rheumatism, malaria, constipation and other conditions. It was later used by other ancient civilizations-the Egyptians (ca. 1700 B.C.), the Indians (ca. 1600 B.C.), the Persians (ca. 750 B.C.), the Greeks and the Romans (ca. 450 B.C.). Historically, *Cannabis* was also used for additional indications such as glaucoma, anal fissures, diarrhoea, as obstetric aid and as anxiety relief [23]. The plant was introduced to the modern western medicine in the early 19th century, and was mainly indicated for treating pain, glaucoma, nausea, depression and neuralgia [24]. 

Today, medical use of *C. sativa* includes multiple indications supported by reliable clinical evidence. Such are the treatment of chronic pain, mutiple sclerosis, resistant epilepsy and chemotherapy-associated nausea and vomiting, apetite and weight loss associated with HIV/AIDS, Tourette syndrome, anxiety disorders, sleep disorders, post-traumatic stress disorder and schizophrenia [1,25,26]. Multiple additional health benefits of *C. sativa* extracts are reported in in vitro and in vivo trials, such as lowering blood cholesterol, triglycerides and blood pressure, antioxidant and antimicrobial activity [27]. *C. sativa* and its extracts are also used in the treatment of dermatitis and degenerative imunological diseases, both as supplements and as traditional medications [25]. Cannabis oils, oral solutions, oil-like concentrates and tinctures used orally and sublingually, lotions, balms, creams, bath salts, salves, gels, patches and other topical products as well as rectal and vaginal products (suppositories, tablets) are the most common pharmaceutical dosage forms employed [26].

Moreover, hemp is additionally used in food and beverage production, as hempseeds are shown to have great nutritional value: high content of protein, containingnine essential amino acids, dietary fibers and an ideal ratio of ω-6:ω-3 fatty acids (3:1). The same applies to hempseed flour and oils, which have high content of proteins, insoluble fibers and polyunsaturated fatty acids. The “cannabis edibles” are the latest type of cannabis-based products that recently became popular. Chewing gums, lollipops, caramel hard candy, berry gummies, lozenges, candy bars, jam, tea, soda, coffee, water, honey, etc., containing Δ^9^-THC- and/or CBD-dominant extracts and concentrates are widely marketed [28]. 

Today, cannabis is the most commonly used illicit drug worldwide, despite the strict international control for more than eight decades [9].

### 1.4. Legal Aspect of C. sativa

*C. sativa* and/or cannabis-based products have been legalized for medical use in 41 countries (23 in Europe) between 2012–2021. As *C. sativa* and cannabis-based products are classified based on the Δ^9^-THC content with psychotropic properties, and most of them contain drug-type *C. sativa* extracts, various legal limitations in many countries still exist. In total, 50 countries in Europe, Asia, North and South America use the plant for industrial purposes [29]. Cultivation and supply of 69 *C. sativa* varieties [30] with Δ^9^-THC content not exceeding 0.2% is legal in EU [31], with some exclusions (Czech Republic and Austria, <0.3%, Switzerland 1.0%).The industrial use of *C. sativa* is focused on production of >2500 products used in agriculture, textile production, recycling, automotive industry, furniture production, paper industry, production of construction materials, energy production, personal care products and medical supplements [29]. 

Cultivation and use of marijuana, the crude drug derived from *C. sativa* for recreational purposes, is not legalized in Europe, but it is decriminalized in 32 countries worldwide (16 in Europe), with various limitations regarding the amount of dry marijuana, number of cultivated plants and punishing public consumption [32].

Several Pharmacopoeias, including the German pharmacopoeia (DAB), Swiss pharmacopoeia (Ph.Helv.), European Pharmacopoeia (Ph.Eur.), and the American Herbal Pharmacopoeia (AHP) comprise monographs defining *Cannabis* flowers (“Cannabis inflorescence”) as herbal substance that consists of whole or crushed, flowering, dried shoot tips of the female plants of *C. sativa* L. (Cannabaceae) that contain 90.0–110.0% of the amounts of phytocannabinoids specified in the label, such as Δ^9^-THC and CBD, as well as cannabinoid carboxylic acids such as Δ^9^-THCA and CBDA, calculated as Δ^9^-THC or CBD, based on the dried drug [33,34,35,36]. 

Due to legal issues, chemotype classification of *C. sativa* is nowadays much more significant. Depending on the content of Δ^9^-THC and CBD, authorities have classified generally three chemotypes of *C. sativa* L.: Δ^9^-THC-predominant type, i.e., drug-type (CBD/Δ^9^-THC = 0.00–0.005), CBD-predominant type, i.e., fiber type (“hemp” type) (CBD/Δ^9^-THC = 15.0–25.0) and intermediate chemotype (CBD/Δ^9^-THC = 0.5–3.0) [37]. AHP proposes more comprehensive chemotype classification. Six chemotypes are defined: (1) type I-Drug (0.5–15% ∆^9^-THC; 0.01–0.16%CBD and 50:1 Δ^9^-THC/CBD ratio); type II-Intermediate (0.5–5% ∆^9^-THC; 0.9–7.3% CBD and 0.25/~2 Δ^9^-THC/CBD ratio); type III-Fiber (0.05–0.70% ∆^9^-THC; 1.0–13.6% CBD and <1:5 Δ^9^-THC/CBD ratio); type IV-CBG (<0.05% ∆^9^-THC; <0.5% CBD); type V-non-cannabinoid (∆^9^-THC = 0; CBD = 0) [33]. In addition, DAB defines a discontinued cannabis extract-*Cannabis* *extractum normatum* as an extract from whole or shredded, flowering, dried shoot tips of the female plants of *C. sativa* L. that contains Δ^9^-THC at least 1% and at most 25% (*m/m*) for the extract and 90.0 to 110.0% of the nominal salary specified in the label, and CBD maximum 10.0% (*m/m*) for the extract and 90.0 to 110.0% of the nominal content stated in the label [38]. High within-chemotype variability is recorded, due to changes in growing and storage conditions, such as environmental factors of cultivation (climates and elevation of cultivated area), the development stage of the plant at harvest time and genetic characteristics of seed-stocks [2,17]. 

### 1.5. Incentive for Investigating Phytocannabinoids in C. sativa and Cannabis-Based Products

Under the pressure of its criminal association, the chemical constitution, pharmacological effects, genetic structure, evolutionary and domestication history of remained poorly understood until the last decade of the 20th century. Authorized investigations related to *C. sativa* were either forensic studies to aid law enforcement or medical and social research specifically intended to document and reduce harmful effects [6]. Since the last decade of the 20th century, a great urge for more thorough investigation of *C. sativa* appeared, mainly as a result of the resurrection of production of *C. sativa* for non-narcotic and medical purposes and the growing tolerance of the extremely widespread recreational use. This increased attention *C. sativa* and cannabis-based products gained due to their high pharmacological potential, updated legislation authorizing many different uses, and, thus, the emerging need to control their quality. This imposes a great challenge for academics, particularly in the field of natural products, from which a contribution to improve and standardize the extraction and characterization of the bioactive compounds from *C. sativa* species is expected. 

Scientific and technological development in regards to *C. sativa* began, highlighting the need of sensitive, specific and robust analytical methods for identification and quantification of the active constituents of *C. sativa*. Chemical profiling of the plant became extremely relevant, since the acquired knowledge further enhanced: (1) chemotaxonomic classification; (2) cultivation set-ups examination, and thus adjustment of cultivation conditions and breeding methodologies in order to produce *C. sativa* varieties with fit-for-purpose physicochemical properties; (3) investigation of potency of seized samples, thus discovering sources of interconnected illegal production and trafficking; (4) association of medical and adverse health effects with potency and/or interplay of certain phytocannabinoids and other active *C. sativa* constituents and (5) QC of medical cannabis and final medical cannabis-based products and potency examination [1,2]. 

The multitude of cannabis-based products including cannabis extracts, oils, resins, pharmaceutical dosage forms, cannabis-infused edibles and beverages are obliged to comply with the national regulatories’ quality control regulations, especially in terms of phytocannabinoids content. Accurate qualitative and quantitative analyses of the phytocannabinoids content and chemical profile in cannabis plants are extremely relevant, in order to associate medicinal and possible adverse health effects with the potency of certain phytocannabinoids and other compounds, such as terpenoids [2]. One of the most relevant problems in analytical determinations for QC, especially when there are legal problems related with quantitation, such as for cannabis, relates to the proficiency of laboratories. Both qualitative and quantitative determinations require carrying out of standardized assays that meet the analytical criteria approved by the relevant control authorities.

This review aims to capture the versatility of analytical methods for natural phytocannabinoids profiling in cannabis and cannabis-based products in the past four decades (1980–2021). As such, this thorough overview is first of its kind. Other most recent reviews cover either a shorter time period, i.e., 2002–2016 [1], 2010–2016 [2], 2009–2019 [39], focus on both plant materials and biological matrices [1,39], describe multiple [2] or single (GC) [39] instrumental analytical platforms, omit cannabis-based products [4] or, apart from phytocannabinoids, also include profiling of other bioactive *C. sativa* constituents [1]. 

## 2. Analytical Methods for Phytocannabinoid Profiling

**Figure 2:**
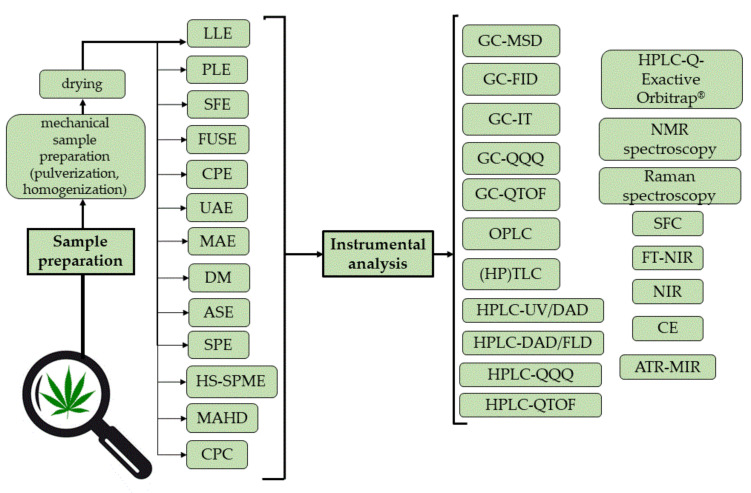
Analytical methods used in phytocannabinoid profiling of cannabis and cannabis-based products.

The overview of the analytical methods for phytocannabinoid profiling used in phytocannabinoid profiling of cannabis and cannabis-based products is schematically presented in Figure 2.

### 2.1. Sample Preparation Techniques

Rapid and simple extraction methods are essential for time- and material-efficient high-throughput phytocannabinoid profiling. Optimization of the three key parameters during extraction is crucial for the overall analytical method [40], which are: (1) the *granulometry* of the solid sample; (2) the *system temperature* and (3) the *affinity* of the extraction liquid towards compounds of interest [41]. *C. sativa* and cannabis-based products are very complex and inhomogeneous matrices, as their different parts may have different cannabinoid profiles due to the variety of phytocannabinoids, terpenes and other volatile compounds and high sugar and fat content. Thus, extraction of phytocannabinoids from plant material and cannabis-based products in an efficient and consistent manner, with acquisition of accurate and reliable potency data can be a challenging task. In addition, there are no standardized preparation procedures for the hemp-based infusions (hemp leaf, hemp-based tea mixtures), and the cannabinoid content could be significantly affected by the infusion preparation procedure [42]. Finally, there is large and unpredictable variability of the average composition as a result of genetic and environmental differences, making the efforts for standardization of sample preparation techniques an ultimatum [43].

Initial steps of sample preparation include mechanical preparation aiming to increase the contact surface between the solvent and the active ingredients. It is of special importance for cannabis plant material, since, despite that most of the active resin is claimed to occur in the superficial glandular trichomes, significant amounts are found in non-glandular tissues. Consequently, immersion of unbroken fresh plant material would give unsatisfactory extraction [44]. Cannabis or its resins are reduced to small pieces by a grater [5] or spatula [45], grinded or pulverized, while cannabis oils are directly proceeded to instrumental analysis.

Manual pulverization and homogenization of the dried plant material can be performed using mortar and pestle [46,47,48,49,50], metal spoon [51] or glass rod [52], by cutting the plant material [53] or crushing and riddling (0.5 mm) [54] or by manual grinder [50,55]. According to the UNODC [5], dried herbal cannabis material and cannabis resins should be pulverized by a cutter (at high revolution speed, i.e., 100 rps) and sieved (mesh size 1 mm). Plant samples can also be homogenized in a crucible [56], in laboratory blender, usually to 60–80 mesh (177–250 µm) particle size, but sometimes a larger (≤335 µm) or smaller (100–150 µm) particle size is required [57,58,59]. Unlike classical, mechanical hand grinders and electrical grinders, some sample preparation methods employ superfine grinding of cannabis plant material [60,61,62]. However, manual grinding with a handheld herb grinder resulted in higher yield of total phytocannabinoids (17.5 ± 0.5%) than with electric blender (12.0 ± 0.3%). The minimization of analyte loss using manual grinder is attributed to the adhesion of cannabis resin to the blades and plastic housing surface of a plastic blender during the high-speed pulverization [50]. Mechanical grinding-activation in an intensity planetary vibrational mill [62,63], ball mill [55], knife mill [64] or freeze mill [65] are also applied. Instead of drying, fresh cannabis plant material can be frozen with liquid nitrogen and crushed [66,67] or frozen, lyophilised at −50 °C and grounded by hand [68] or in a mill [69]. Comparison of coarse homogenization by sieving through a 1-mm mesh and fine homogenization with a ball mill revealed better extraction efficiency for CBDA and THCA for the finely powdered plant material, and no difference for the neutral phytocannabinoids (CBD, Δ^9^-THC) [55].

In the next step, thermal processing occurs, which aims to remove moisture, usually to 8–13% residual humidity, as recommended by UNODC [5], achieved by drying at room temperature for several days or at 70 °C until the leaves become brittle or, according to EC, within 48 h using any method below 70 °C [70]. Lower drying temperatures should be avoided, as they result in mould growth [71]. Despite this, drying is frequently performed at variety of temperatures and durations, such as at 135 °C, 2 h, 120 °C [72], 103 °C, 4 h [73], 65 °C, 16 h [74] or 48 h [75,76], 60 °C, 12 h [77], 40–50 °C [54], 40 °C,72 h [78], 48 h [17,79] or 24 h [74,80,81], 38 °C, 4–8 h [82], 35 °C, 24 h [83], 30–40 °C, 1–2 days [46], 30 °C [57] on forced ventilation oven, by natural ventilation at 32 °C for 60 h [84], at 30 °C overnight [52] or for 4 h [80] or air-dried at room temperature (20–22 °C) for 24 h [81], for 3 days [44,85], 6 days after harvesting [86], for 4 weeks [87] or until a residual humidity ≤12% is achieved [47].

Dried and mechanically processed samples are extracted using maceration, LLE, PLE, HS-SPME, SFE or FUSE. Other extraction techniques, including ultrasonic assisted extraction (UAE), microwave assisted extraction (MAE), dynamic maceration (DM) and accelerated solvent extraction (ASE), are faster and use less extraction fluids than the “classic” maceration. The number of the consecutive extractions did not have significant effects on total phytocannabinoid yield. Yet, phytocannabinoids yield after sonication was found to be slightly lower than the yield obtained by one-day DM [50]. Phytocannabinoid extraction was omitted in only one study, which, consequently, reported low sensitivity (Table S1); thus difficulties occurred during quantification of trace phytocannabinoidsin cannabis plant tissues [88]. The summary of the properties of the most frequently used sample preparation techniques are given in Table 2.

The conventional sample preparation methods for cannabis plant material are maceration and LLE using versatile organic solvents with great affinity towards phytocannabinoids. Although universal and simple, they are time-consuming and not environment-friendly, as they require large quantity of organic solvents.

UNODC recommends maceration prior to GC-flame ionization detector (FID) analysis; 0.2 g dried and homogenized herbal cannabis, 0.1 g cannabis resin or 0.05 g cannabis oil is extracted with internal standard (IS) solution of tribenzylamine in 96% EtOH (0.5 mg/mL) for 15 min in an ultrasonic bath [5]. DAB’s *Cannabis flos* monograph proposes extraction with EtOH (96%, *v/v*), while the AHP’s Cannabis inflorescence monograph proposes extraction with MeOH/CHCl_3_ (9:1, *v/v*) [33]. EC recommends maceration of 0.1 g semi-fine powdered herbal cannabis with IS solution of 35 mg of squalane/100 mL hexane [70].

The most commonly used solvent and solvent mixtures for extraction of phytocannabinoids from cannabis and cannabis-based products are given in Table 3. 

Absolute EtOH is the most preferred organic solvent for maceration and LLE due to its great affinity for phytocannabinoid structure [54,115,146] that leads to high extraction efficiencies. EtOH is, however, known to co-extract significant amount of pigments and ballast from cannabis plant material, much more than CHCl_3_, enhancing matrix interferences [44]. To avoid this, *n-*hexane is used [87]. For highly aqueous cannabis-based products, such as coffee beverages, 100% MeCN is preferred LLE solvent [90]. CHCl_3_ is preferred for extraction of dried glandular plant material (leaves), cannabis resin and reefers (mixtures of tobacco with powdered resin or herbal cannabis), yielding 99% extraction efficiency, much higher than light petroleum [44]. Hexane was found to be the worst performer in terms of recovery of all phytocannabinoids, while EtOH was shown to possess the most appropriate polarity for cannabinoid compounds [40]. 

Prior to TLC/HPTLC analysis, maceration and/or LLE is the extraction technique of choice. Phytocannabinoids are extracted from *cannabis* plant material using MeOH, according to Ph.Eur., Ph.Helv. and DAB [34,35,36] and published studies [147], dichloromethane, according to AHP [33], solvent of choice (petroleum ether, MeOH, *n-*hexane, toluene, CHCl_3_ and solvent mixtures, e.g., MeOH/CHCl_3_ (9:1, *v/v*)), according to UNODC [5]. Prior to maceration/LLE, lactose-containing cannabis products (cream cheese, butter, coffee beverages with milk) require lactase pre-treatment/lactose hydrolysis in order to avoid matrix interferences [90].

Maceration and LLE of semisolid fatty/oily matrices of cannabis-based products, such as butter, margarine, chocolate bars, nonpolar topical ointments and balms requires warming of the samples on a hot plate after addition of the extraction solvent in order to melt the matrix. Prior to maceration/LLE, lactose-containing cannabis products (cream cheese, butter, coffee beverages with milk) require lactase pre-treatment/lactose hydrolysis in order to avoid matrix interferences [90]. For high sugar and carbohydrate matrices including hard candies, honey and fruit preserves, a matrix trapping effect occurs, resulting in the formation of glassy, impervious precipitates which are hard to extract. To avoid this, the aqueous portion of the extractant is added first, followed by warming the matrix in order to dissolve the sugars and carbohydrates, and addition of ACN (to 83–91% final proportion) [90]. Maceration and LLE of cannabis-based products is further aggravated by the presence of glycerine and propylene glycol, especially in oral supplements and vape products. They interfere cannabinoid profiling significantly. High levels of co-extracted glycerine or propylene glycol may swamp the silylating derivatization agents, disabling complete derivatization of CBD to CBD-2TMS, along with undesired side conversion of Δ^8^-THC and Δ^9^-THC. The problem can be avoided with CAN extraction, as sugars and glycerine have much lower solubility in ACN than in EtOH [90].

While most of the methods include vortexing, ultrasonication in bath and centrifugation immediately after solvent addition, older methods include immersion, e.g., for 1 h [9,75,121,143,144], overnight [121], 10-days soaking [123], several hours’ maceration [87] or heating near boiling for few hours in solvent, followed by separation of the liquid extract by filtration [108]. Only one study evaluated the shaking time during extraction with MeOH/CHCl_3_ (9:1, *v/v*) at room temperature in a range from 10 min to overnight shaking and showed that 20 min of shaking gained sufficient extraction efficiency for Δ^9^-THC, CBD and CBN [101]. Finally, exhaustive extractions in Soxhlet apparatus are rarely performed [95,117]. In terms of temperature conditions, extraction is performed with highest efficiency at room temperature, with rare exemptions (e.g., at 4 °C) [77], in order to avoid conversion of phytocannabinoids from plant material [1]. If additional heating is required (e.g., when no preliminary decarboxylation was performed), hot extraction (e.g., at 70–78 °C) is performed by sonication [92] or in Soxhlet apparatus [95,117].

After extraction of cannabis plant material, additional filtrationis performed, using sintered glass disc [44], sterile cotton plugs [9,75,143,144], PFTE syringe filters, with 0.22 µm [68,111] or 0.45 µm pore size [56,94,95,108], millipore filter (0.45 µm) [50,84], nylon filters (0.45 µm) [133], membrane filter [49], PVD filter (0.22 µm) [113] or, alternatively, drying on MgSO_4_ and filtration [67,138]. The insoluble plant material can also be removed by vacuum filtration. The filtered extract is usually dried under nitrogen and dissolved in IS (androst-4-ene-3,17-dione) in EtOH [58,77], or in anhydrous pyridine [119]. Alternatively, IS can be added directly to the supernatant after filtration [44] or centrifugation [10,103,106,111]. As filtration cannot be performed efficiently for cannabis resin extracts due to filter clogging by felt of trichomes and other vegetable debris [44], centrifugation is only performed.

Solvent exchange may also be included as final step, by diluting extracts with solvents and solvent mixtures more similar to the mobile phases employed, in case of (HP)LC analysis. For example, cannabis-infused chocolate is first soaked in IS solutions and isopropyl alcohol, extracted with MeCN + 1% acetic acid and finally diluted with MeCN prior to LC-MS/MS or MeCN/H_2_O (75:25, *v/v*) prior to HPLC-UV analysis [65]. 

Solvent exchange is also performed prior to GC analysis, especially when derivatization is performed [7,80,87,104,121,123,133]. Extracts are reduced to dryness usually under gentle N_2_ steam, causing least damage to total extracted amounts of phytocannabinoids and terpenoids than drying in rotary evaporator or in a speedvac, the latter reducing the concentrations of Δ^9^-THC and CBG for two-thirds [66]. Reduced extracts can be dissolved in solvents (e.g., pyridine and benzene [116], CHCl_3_ [5], MeCN [116], dry EtAc [55]), in a mixture of derivatization agent and solvent (e.g., toluene and BSTFA [94], pyridine and BSTFA + 1% TMCS [118], pyridine, isooctane and MSTFA [99], pyridine and BSTFA [90], pyridine and MSTFA + 1% TMCS [84]) or directly in the derivatization agent [80,99,100,133]. As silylation agents are harmful for GC injection port and column, additional evaporation to dryness is frequently performed, followed by dissolution in solvent, e.g., *n-*hexane [84] or MeCN [95]. If no derivatization is performed, EtAc [120,126,127] and EtOH [66,123] are the most commonly used solvents for reconstitution prior to GC analysis.

Before instrumental analysis, decarboxylation may be also introduced, initiating thermal degradation of phytocannabinoid acids to neutral counterparts for the purpose of accurate phytocannabinoid profiling and potency examination. Dried extracts are most commonly heated at 150–210 °C for 10–30 min and reconstituted in the same solvent or solvent mixtures [7,52,60,101,128], or, alternatively, at 50 °C for 180 min and then 145 °C for 15 min [69]. A total 15 min of decarboxylation at a temperature range of 120–180 °C showed that maximum yield is achieved at 140–160 °C, with no significant within-range differences [115]. Decarboxylation temperatures higher than 160 °C should be avoided, as Δ^9^-THC is oxidized to CBN [115] and isomerised to Δ^8^-THC [87]. However, it is almost impossible for decarboxylation to yield 100%, which initiates significant discrepancies in potency data. Laboratories quantifying the total Δ^9^-THC as the sum of the Δ^9^-THC already present in the plant and Δ^9^-THCA get higher values than laboratories that perform decarboxylation prior to instrumental analysis [115].

Once prepared, cannabis extract should be stored in light-protected conditions, at room temperature, refrigerated at 4 °C [17,48,84,126], at −20 °C [53,103] or at −80 °C [78] prior to instrumental analysis.

HS-SPME is a solvent-free sample preparation method used for analysis of phytocannabinoids in the headspace over solutions or solid samples [5]. Multiple factors affect the extraction efficiency during HS-SPME, including SPME fiber coating, exposure temperature, extraction time and desorption time. Evaluation of the effect of the fiber coating on extraction efficiencies of Δ^9^-THC, CBD and CBN from herbal cannabis samples showed that among polydimethylsiloxane (PDMS) 100 µm, PDMS/divinylbenzene (PDMS/DVB) 65 µm, Carboxen^®^/PDMS and divinylbenzene/Carboxen^®^/PDMS (DVB/Carboxen^®^/PDMS) 50/30 µm, PDMS 100 µm performed optimally in general, although the PDMS/DVB fiber provided higher extraction efficiency for CBD, due to its higher polarity and affinity to PDMS/DVB. Among the three exposure temperatures (80 °C, 90 °C, 150 °C), 150 °C was optimal, simultaneously promoting volatilization and decarboxylation [59,148]. Extraction time depends upon matrix viscosity and lipophilicity that define the speed of diffusion of analytes from the liquid to the gas phase and, as a result, HS-SPME rate and efficiency. HS-SPME is more appropriate for simpler matrices (e.g., cannabis tea), as the extraction recoveries are proportional to the sample amount [99], while complex liquid- and protein-containing matrices cause significant matrix retention and lower recoveries, with higher LODs and lower method precision. For fatty/oily matrices, such as versatile hemp foods, alkaline hydrolysis with NaOH and Na_2_CO_3_ is performed prior to HS-SPME in order to saponify the matrix lipids and reduce lipid matrix interferences [99]. Therefore, extraction time varies depending upon the sample matrix, from 10 min for herbal cannabis [59,148] to 25 min for different hemp food products [99]. Finally, desorption time depends upon analytes’ lipophilicities [148]. It is superior to LLE (*n-*hexane/EtAc (9:1, *v/v*)) in terms of chromatographic peak shape and matrix interferences, despite the good agreement of achieved LODs in food samples [99].

SFE uses supercritical fluids (SCFs) and liquefied gases as green solvents for extraction and fractionation of complex samples. SFE offers low solvent consumption and ensures stability of thermolabile and light-sensitive compounds. For the purpose of phytocannabinoid profiling, SFE is rarely used in sample preparation; its main purpose is to separate the aromatic fraction for further analysis. It is usually performed using supercritical CO_2_ (SC-CO_2_) as attractive SCF with solvent strength tuned by sensitive changes in temperature and pressure above the critical point (31.1 °C, 73.7 bar), conditions that are experimentally easy to reach [4], along with the low cost, short processing time and low environmental impact [149]. However, SC-CO_2_ is a low polarity solvent that poorly dissolves phytocannabinoids; therefore, employment of co-solvent, usually H_2_O, alcohols and acids, to improve the overall extraction rate of phytocannabinoids is required. EtOH (5–20% in CO_2_) is most commonly used co-solvent, added in constant flow [40,43,143,150,151] or in pulses [143]. Higher SC-CO_2_ pressures offer lower extraction selectivity, but high initial extraction rate, apparent solubility and total yield, which are also a function of temperature, exposure time and phytocannabinoid content of the plant material [143]. A 90–94% extraction yields for Δ^9^-THC, CBD and CBN are achieved at 100 bar, 35 °C and 1 mL/min flow during 10 min [43,150]; at 340 bar, 55 °C and 200 g/min maximal yields up to 92% are reported [143], along with satisfactory yields at milder conditions, (37 °C, 250 bar). Extraction efficiency was further improved by washing of the extract with fresh SC-CO_2_ and addition of a cold separator (separating chamber) immediately after the sample containing chamber [149].

When compared to DM, UAE and MAE with same extraction solvent (EtOH) in same *w/v* ratio to sample, no significant difference existed between SFE and UAE, with the lowest extraction yields for CBD, CBDA and CBGA. DM and MAE showed higher yield for CBD and CBGA, but DM was selected as the optimal sample preparation technique (EtOH, room °C, 45 min). In case of MAE, increased CBD yield was accompanied by decreased CBDA yield, suggesting partial decarboxylation due to high extraction temperature [142].

FUSE is used for phytocannabinoid extraction from herbal cannabis, employing cyclohexane/isopropanol (1:1, *v/v*) in an ice-water media in order to avoid degradation and solvent evaporation, followed by centrifugation and filtration through 0.45 µm nylon filter [43]. FUSE is slightly more efficient than SFE for extraction of Δ^9^-THC, CBD and CBN, as 80% of the phytocannabinoids are extracted at the first extraction, while <40% are extracted after the third extraction with pure SC-CO_2,_ and more than 90% are extracted with the first extraction in the presence of co-solvent. Therefore, adding the ability of SFE to separate terpenes from phytocannabinoids, and therefore minimize matrix interferences, SFE was selected as more optimal sample preparation technique [43].

PLE is one of the fastest and most efficient extraction techniques for plant metabolites. High extraction yields are achieved under pressure, using the extractant at a temperature above its normal boiling point, thus increasing its diffusion into the plant matrix. PLE on herbal cannabis is performed using *n-*hexane as extraction solvent at 100 °C and 40 bar for 15 min. MeOH and *n-*hexane are found equally efficient for Δ^9^-THC and CBN, but not for Δ^9^-THCA, as it is less soluble in *n-*hexane [95]. PLE with hot water, i.e., pressurized hot water extraction (PHWE) is used to yield CBD-rich extracts while supressing the THC and CBN content. Here, decarboxylation is performed in situ, i.e., in the extraction cell, heated in the oven prior to the dynamic extraction [80].

SPE with QuEchERS is used for purification of honey extracts, with high recoveries for CBDA, CBGA, Δ^9^-THCA, CBG, CBD and Δ^9^-THC and low intra- and inter-day variability. The method was more efficient than UAE with H_2_O at 40 °C, yielding homogeneous solution with no phase separation or solid residues, followed by extraction of phytocannabinoids from the aqueous phase through LLE with *n-*hexane or EtAc, observing higher phytocannabinoid yield in *n-*hexane extracts [40]. QuEChERS is also used for purification of MeCN extracts of hemp seeds, hempseed oil, hemp proteins, raw and skimmed milk, coffee and chocolate, using reaction mixture of MgSO_4_/NaCl/C_6_H_5_Na_3_O_7_ × 2H_2_O/disodium hydrogen citrate sesquihydrate (4:1:1:0.5, *w/w*); with further supernatant dilution with MeCN/H_2_O (1:1) or with H_2_O. d-SPE with MgSO_4_, different combinations of C_18_, primary secondary amines and zirconia-coated silica sorbents were also evaluated. When PSA is used, cannabinoids were trapped by interactions with amines, resulting in low recoveries; other combinations achieved satisfactory extraction [144].

Cloud point extraction (CPE) involves employment of non-ionic surfactant, salt (Na_2_SO_4_) and deionized water to extract Δ^9^-THC from cannabis resin using heating (40–90 °C) and centrifugation. Despite the low extraction efficiency (60%), CPE offers many advantages, such as the possibility of extraction and pre-concentration of analytes in a single, solvent-free step and avoidance of analyte loss during solvent evaporation [145].

Centrifugal partition chromatography (CPC) is a liquid-liquid partitioning technique in which the stationary phase is immobilized by centrifugation force, while the mobile phase is pumped through at high flow rates. Compounds are separated based on the differences in partition coefficients. CPC allows large-scale extraction of phytocannabinoids, i.e., Δ^9^-THC, CBD, CBN, CBG, Δ^9^-THCA, CBGA and CBDA with high efficiency (>90%) from herbal cannabis material using two-phase system *n*-hexane/MeOH/H_2_O with 25 mM formic acid (5:3:2, *v/v/v*) for Δ^9^-THCA, CBGA and CBDA and hexane/acetone/MeCN (5:2:3, *v/v/v*) [87].

Microwave-assisted hydrodistillation (MAHD) can provide a volatile hydrodistillate that is rich in monoterpenes, sesquiterpenes, and a small amount of phytocannabinoids. The optimized MAHD procedure in a pilot-scale reactor yielded 0.35 ± 0.02% *w/w* of hydrodistillate, while conventional hydrodistillation gave only 0.12 ± 0.01%, *w/w* (in relation to dry inflorescence mass). During MAHD, phytocannabinoid decarboxylation inside the residual matrix was around 70% (69.01 ± 0.98% and 74.32 ± 1.02% for THC and CBD respectively) [146]. In other studies, MAHD resulting essential oils are dominant in CBD content (2.11–20.06 mg/g); interestingly, the essential oils from dried plant material also contain CBDV, CBL and cannabicitran (CBT) [152].

### 2.2. Instrumental Analysis

**Figure 3:**
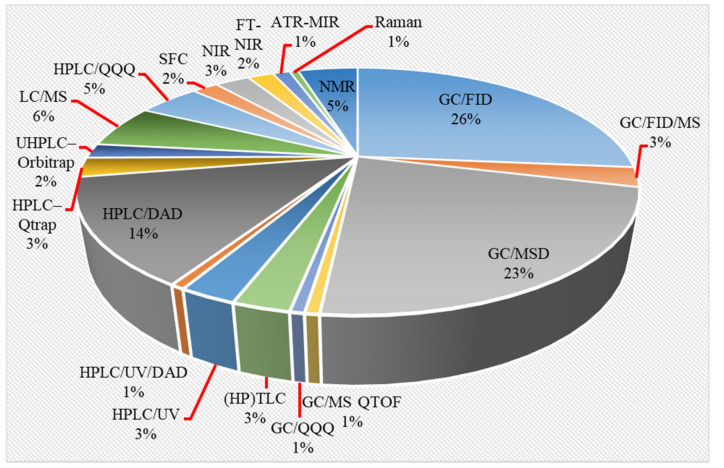
Prevalence of analytical techniques used for phytocannabinoid analyses.

Influenced by the intense scientific and technological development in regard to *C. sativa* cultivation, analytical platforms for phytocannabinoid profiling in cannabis and cannabis-based products have intensively evolved over the last four decades (Figure 3). GC- and LC-based methods as most commonly used, have achieved comparable accuracy, selectivity, linearity, sensitivity and precision in phytocannabinoid profiling and are both used in routine and investigational analysis of cannabis and cannabis-based products. 

Despite the lack of standardization process for analysis of phytocannabinoids omitting the comparison of reliability of measurement among analytical platforms, a recent interlaboratory study concluded that GC-MS is the most accurate and robust analytical method for phytocannabinoid profiling, performing much better than GC-FID and UHPLC-MS/MS [55].

The advantages and disadvantages of the most frequently used analytical techniques for analysis of cannabis and different products are given in Table 4.

#### 2.2.1. GC-Based Methods

GC coupled to versatile detectors and mass analyzers is one of the oldest, but still the most preferred and researched analytical platforms for phytocannabinoid profiling in both plant material and biological matrices due to its robustness, reproducibility, sensitivity and speed [1,42,58,153]. As such, GC methods are officially employed by authorities for phytocannabinoid profiling [1], including the predominant phytocannabinoids (Δ^9^-THC, CBD and CBN) and quantification of Δ^9^-THC/CBD ratio. This analytical platform is also used for terpene profiling, pesticide screening and residual solvents analysis, which affords potential benefits to regulatory bodies and cannabis industry [39]. 

By combining short columns, fast oven temperature ramps, high carrier gas linear velocities, narrow columns, hydrogen carrier gas and low film thickness, fast and robust GC methods are generated, appropriate for phytocannabinoid profiling in both research and monitoring purposes [39]. The access to the well-established MSLs, such as the National Institute of Standards and Technology (NIST) Mass Spectral Library and the Wiley Registry Mass Spectral Library eases compound identification through GC-MS analysis. Here, phytocannabinoids identification is performed by comparison of acquired MS or MS/MS spectrum to spectra present MSLs, and further confirmed by analysis of analytical standards. Although the main employment of GC-based platforms is for profiling of terpenes, it has been extensively used for phytocannabinoid profiling as well (Table S1).

- Derivatizationof Phytocannabinoids

First action, after injection of the sample in the injector port of the GC (regardless of employed detector), is vaporization that is achieved at temperature ranges 250–290 °C and causes in situ decarboxylation of acidic phytocannabinoids (Δ^9^-THCA, CBDA, CBGA) to the corresponding neutral phytocannabinoids (Δ^9^-THC, CBD, CBN) prior to chromatographic separation. Thus, acidic and neutral phytocannabinoids are not distinguishable, but rather the result is the sum of neutral cannabinoid present in the extract and neutral cannabinoid generated during decarboxylation. The issue is of no concern for studies aiming to quantify total THC (Δ^9^-THC and Δ^9^-THCA) levels, as it is the case with most of the GC-based studies included in this review without prior derivatization [9,17,18,45,47,48,49,52,54,56,58,59,63,67,72,73,74,75,76,79,81,82,85,86,89,93,96,98,101,102,103,105,108,115,121,122,123,124,128,138,139,141,148,150,152,154,155,156,157,158,159,160] (Table S1). However, derivatization is of great importance for studies aiming more thorough phytocannabinoid profiling. In order to prevent their degradation and achieve profiling of the native chemical constitution of the cannabis material, a reaction of derivatization has to be performed prior to GC analysis. In such a way, derivatization improves the limited volatility and thermal stability of phytocannabinoids, and thus their amenability to GC analysis, which further improves peak shape, peak resolution (especially for CBC and CBD) and sensitivity [161]. This comes at the price of increased analyses cost, duration [2] and measurement uncertainty, as derivatization yields are sometimes highly variable and seldomly obtain 100% for phytocannabinoids [23,91,122], making quantification results speculative.

Silylation is the most common derivatization reaction performed. It involves substitution of a hydrogen atom that is bound to a hetero atom (such as -OH, -COOH, -NH_2_, =NH, and -SH) by a silyl group, i.e., a trimethylsilyl (TMS) or *tert-*butyldimethylsilyl group (TBDMS). The resulting TMS/TBDMS derivatives have lower polarity and increased thermal and catalytic stability and GC amenability. However, they can be thermally degraded in injector port and/or column system [162].

Versatile silylation agents are used, ordered by reactivity: hexamethyldisilazine (HMDS) [88], N-methyl-(trimethylsilyl) trifluoroacetamide (MSTFA) [153], N, O-bistrifluoroacetamide (BSTFA) [90], alone or accompanied by a catalyst, usually 1% trimethylchlorosilane (TMCS) [58,99,100,123,124,133] for TMS derivatization and N-*tert-*butyldimethylsilyl-N-methyltrifluoroacetamide (MTBSTFA) alone or with 1% tert-butyldimethylchlorosilane (t-BDMCS) as a catalyst for TBDMS derivatization. A study comparing the derivatization efficiency of different alkylsilylation agents, HMDS + trifluoroacetic acid (TFA), MSTFA, activated MSTFA, MSTFA + 1% TMCS, BSTFA, BSTFA + 1% TMCS, MTBSTFA and MTBSTFA + 1% t-BDMSC, using pyridine as additional catalyst for CBC, CBD, CBG, CBN, Δ^9^-THC and Δ^9^-THCA in standard solutions and plant matrix concluded that maximum responses are obtained with HMDS + TFA. Responses were not influenced by catalysts, as well as reaction solvent (pyridine, EtAc, MeCN) [88]. Other less frequently exploited derivatization is methylation of the hydroxyl groups of the phytocannabinoids using trimethylsulfonium hydroxide (TMSH) [97]. Formation of alkylboronate-TMS derivatives using alkylboronic acid and BSTFA + TMCS showed that methyl- and n-butylboronates yielded derivatives that were stable for several weeks at 4 °C had improved GC peaks when compared to TMS derivatives, and MS characteristics, with preserved fragmentation patterns of the underivatized compound [116].

- GC Columns

Various GC-based methods became popular and widely used during the 40-year span of phytocannabinoids profiling (Table S1). E.C. recommends use of glass capillary column 25 m long and 0.22 mm wide, impregnated with 5% non-polar phenyl-methyl-siloxane phase, that would allow good separation of phytocannabinoids [70]. Older GC-based methods employ glass columns packed with 2% OV-17 on Chromosorb WHP (mesh 100–120) [119], 2% OV-17 on 100–200 mesh GasChrom Q) [98,123,140], 2% OV-17 [58] and glass column packed with 3% SE-30 on 100/200 mesh Gas Chrom Q [116]. Later, they were replaced by capillary columns with cross-linked and bonded stationary phases with various polarity. Here, analytes are separated based on differences in polarity, molecular mass and boiling point. As most frequently, phytocannabinoid profiling studies are focused on the most prominent phytocannabinoids, that are Δ^8^-THC, Δ^9^-THC, CBC, CBD, CBDA, CBDV, CBG, CBGA, CBN, Δ^9^-THCA and THCV. They contain aromatic, alkyl and alcohol moieties; it is expected that the proportion of phenyl groups in mixed dimethylpolysiloxane-silphenylene or mixed dimethylpolysiloxane-dimethyl-dimephenyl stationary phases to have an impact on their chromatographic separation. Wide employed thin-filmed capillary columns with non-polar stationary phases are used, such as 5%-diphenyl-dimethylpolysiloxane columns, including HP-5 (for FID) or HP-5MS (for MS) [50,52,57,58,62,70,85,89,91,94,101,103,104,126,133,141,142,154,156], DB-5MS [9,99,108,128,151,163,164], Rxi-5MS [73], Mega-5MS [146], BP-5 [45], RTX-5 [94,161], MDN-5S [91], SE-52 [128], ZB-5 [95], Zebron ZB-5HT Inferno [102] and SLB-5MS [152]. 100% dimethylpolysiloxane columns, such as HP-1 [10,109,112,165], SPB-1 [85], OV-1 [74] and DB-1 [17,23,75,77,84,144,148] are preferred for more successful separation of CBC and CBD, apart from all other phytocannabinoids, or are used only for separation of CBC and CBD [10,109,112]. Such columns are appropriate for analysis of CBDV, THCV, CBD, CBC, Δ^8^-THC, Δ^9^-THC, CBN but not for CBG [163]. Simultaneous injection on two column with different polarities, i.e., a medium-polar (HP-50+) and non-polar column (DB-1MS) is another option for better separation of CBC and CBD [18]. 

Columns with intermediate polarity, such as Rxi-35Sil MS [90] and DB-35MS [163] (35% (-phenyl)methylpolysiloxane) and Zebron ZB-624 (6%- cyanopropyl-phenyl, 94-dimethylpolysiloxane) [106] are also used for cannabinoid separation. Rxi-35Sil MS may offer slightly wider retention window than a 5% silphenylene phase (DG-5MS) and 5% diphenyl phase (HP-5MS) [90]. However, columns with 35% (phenyl)methylpolysiloxane stationary phase might produce multiple peaks for CBC, one of which coeluted with THCV. Low to mid-polarity columns, such as DB-170 (14%-cyanopropyl-phenyl)-methylpolysiloxane) are not appropriate, as they produce low responses, distorted peak shape and tailing [163]. Despite this, the nonpolar 100% dimethylpolysiloxane column DB-1HT was used for successful separation of Δ^9^-THC, CBD, CBC, CBN and other minor cannabinoids, along with terpenes, sesquiterpenes, sterols, diglycerides and triglycerides [166].

- GC Detectors

A large proportion of the profiling studies employ FID [10,17,49,52,55,56,58,70,73,74,75,76,79,82,85,93,94,95,96,98,101,102,103,106,108,115,118,119,138,141,157,158,159] or dual FID [9,75,121,144]. FID offers more accurate quantitative response with respect to MS, but at the price of lower sensitivity and specificity. The need of analogue analytical standards only makes FID low-cost and simpler analyzer when compared to MS, which needs the use of the corresponding deuterated analytical standards [1]. Moreover, GC-FID methods are more robust than GC-MS methods both in full scan and SIM mode [107]. However, due to its inability to discriminate CBD and CBC, FID is often replaced by MS detectors for more thorough cannabinoid profiling.

GC-MS is the most researched analytical platform for cannabinoid profiling to date [1,42,153,167]. Electron impact ionization (EI) is the most commonly used ionization technique in cannabinoid profiling, while others, i.e., chemical ionization (CI) in positive/negative mode and atmospheric pressure ionization (API) are preferred in forensic analysis of phytocannabinoids in biological samples. Mass selective detector (MSD) [45,47,49,55,59,74,81,86,89,90,94,96,121,122,123,124,128,139,146,148,154,160,166], single quadrupole (Q) [57,66,76,163] and triple quadrupole (QQQ) are the most frequently used configurations. Improved sensitivity, specificity and reproducibility, with reduced noise level and interferences are achieved by monitoring specific ions (selected ion monitoring, SIM mode, MSDs and Qs) or fragmentation reactions (multiple reaction monitoring mode, MRM mode, QQQ), then using the analyzers in scan mode. Ion trap (IT) is seldomly used [23,70,151], despite its advantageous ability to acquire structural information by higher fragmentation (MS^3^, MS^4^ … MS^n^). Quadrupole-time-of-flight (Q-TOF) mass analyzer is rarely used for quantification purposes, but rather for untargeted cannabinoid profiling of *C. sativa* extracts [78]. Rarely, FID and Q are simultaneously used by installing a “Y” splitting unit at the column outlet [152]. Finally, a novel variation of GC-EI-MS, cold EI, based on interfacing GC and MS with supersonic molecular beams (SMB) in a fly-through ion source was recently successfully employed for an inaccurate, sensitive, reproducible and comprehensive full (including phytocannabinoid) profiling of herbal cannabis extracts [166].

A relatively novel GC-based analytical platform employs analyser based on vacuum UV (VUV) operating in the UV/VUV spectral range (120–240 nm). UV/VUV absorption events are very sensitive for differentiating isomers (positional isomers and diastereomers). This, together with the ability to deconvolute overlapping spectra [4], which significantly shortens analysis time, makes GC-UV/VUV potentially favourized analytical platform over GC-MS. Almost all phytocannabinoids exhibit maximum absorbance in the region 170–200 nm, with no overlap among different phytocannabinoids and significant spectral differentiability. This is especially important for CBC and CBD. However, this method reports high LODs and, thus, cannot be employed for phytocannabinoid profiling in biological matrices and cannabis-based products, but is sufficiently sensitive for plant matrices [161].

Two-dimensional (2D) gas chromatography (GC × GC) is reported to offer better chromatographic resolution of phytocannabinoids [11,56,150,160]. Despite this, it is rarely used for phytocannabinoid profiling; chemical fingerprinting and classification are more commonly performed GC x GC is usually coupled to MS [56,160] or to FID/MS [150]. Combination of columns of different polarity is used, as, for example, 100% dimethylpolysiloxane and polyethylene glycol in sol-gel matrix [56], or a non-polar column (e.g., DB-5 or HP-5MS) for first-dimension separation and medium-polarity column (e.g., DB-17) in the second dimension [150,160].

#### 2.2.2. LC-Based Methods

LC-based methods are recently becoming methods of choice for qualitative and quantitative phytocannabinoid profiling. The simplified sample preparation and the low temperatures, high pressure and high flow rates used during TLC, HPTLC, HPLC and UHPLC, and the recently emerging supercritical fluid chromatography (SFC) analysis allow sample preservation without decarboxylation and decomposition, reliable separation of neutral and acidic phytocannabinoid species and, thus, direct identification and quantification of both neutral and acidic forms of phytocannabinoids in the extracted samples [18,165]. The simplified sample preparation methods, along with avoidance of analytes loss favourized LC over GC in cannabinoid profiling [66].

- TLC and HPTLC Methods

TLC is an attractive method for analyses of herbal drug constituents [168], and especially suitable method for the purpose of preliminary semi-quantitative screening of cannabinoid content in routine tests [164]. It is method of choice for identification of cannabis flowers in all *Cannabis flos* monographs (DAB, AHP, Ph.Helv., Ph.Eur.) [33,34,35,36] and DAB’s *Cannabis extractum normatum* monograph [38]. Using TLC, cannabinoid identification is performed by comparing retardation factors (R_F_s) of analytes with R_F_s of standards on a TLC plate developed with appropriate mobile phase, whereas visual evaluation is obtained by dipping or spraying the TLC plate into/with the appropriate visualization reagent under UV light, or under daylight. In DAB [36] and Ph.Eur. [35] analytical monographs of cannabis flower, a MeOH extract of 0.1 g pulverized drug is identified by comparison of R_F_s of analytes to reference solutions of CBD and Δ^9^-THCA (5 mg each) in MeOH. Solutions are applied on TLC C_18_ silica gel F_254_ plate (2 to 10 μm) and developed with H_2_O/glacial acetic acid/MeOH (15:15:70, *v/v/v*). After air drying, the TLC plate is sprayed with vanillin reagent, dried at 100–105 °C for 15 min and examined on daylight. Identical procedure applies to *Cannabis extractum normatum* according to DAB [38]. Ph.Helv. includes filtration of MeOH extract of cannabis flower through membrane filter (0.45 μm) as an additional step prior to application to the C_18_ silica gel F_254_ plate and reference solution of CBDA in MeCN and Δ^9^-THCA in 2-isopropanol, following the identical procedure for development and detection [34]. AHP’s cannabis flower monograph employs TLC C_18_ F_254_ plate with MeOH/H_2_O with 1% glacial acetic acid (75:25, *v/v*) as a mobile phase for identification of CBC, Δ^9^-THC, CBN, CBG, CBD THCV, Δ^9^-THCA and CBDA in dichloromethane extract of 0.1 g pulverized drug. Visualization is performed using Fast Blue reagent and vanillin/H_2_SO_4_ under UV (254 nm) [33]. UNODC suggests maceration in ultrasound bath with 10 mL of solvent (MeOH, petroleum ether, *n*-hexane, toluene, CHCl_3_ or solvent combinations–MeOH:CHCl_3_ (9:1, *v/v*) for 15 min at room °C, using three systems for elution of HPTLC silica gel plates (A: petroleum ether 60/90/diethyl ether (80:20, *v/v*); B: cyclohexane/di-isopropyl ether/diethylamine (52:40:8, *v/v*) and C (for cannabinoid acids): *n-*hexane:dioxane/MeOH (70:20:10, *v/v*)). Fast Blue reagent BB or RR in MeOH or MeOH:H_2_O is used as spaying reagent using visualization method 1 or method 2 [5].

The accuracy, repeatability and the acceptable LODs and LOQs in the linear dynamic range of this methodology makes TLC methods attractive for fingerprinting cannabis [4]; however, such parameters are fairly low compared to the more sophisticated LC analytical platforms. The “classic” TLC became further less utilized due to its inconvenience to document for peer review, poor resolution due to systematic errors rising from hand-spotting, temperature/humidity control and imprecise R_F_ measurement [167,169].

Recent advances in TLC are in the development of HPTLC methods. Such reliable methods could offer advantages over both HPLC and GC techniques for cannabis profiling, including its ability to analyse multiple samples simultaneously and the consequently lower running costs and analysis runtime. Further, automation of sample application in HPTLC methods eliminate systematic errors, provide better resolution and generate reports for more convenient documentation for peer review of casework [170]. In that spirit, normal-phase HPTLC with an automated spotter is shown to achieve better separation than TLC for the main neutral phytocannabinoids. The method is comparable within a small degree of error (±0.5%) to a validated HPLC method [110].

Older methods use plates spread with a layer of a slurry of alumina/CaSO_4_/H_2_O (22.0 g:3.0 g:50 mL), activated at 110 °C for 30 min and stored under anhydrous CaCl_2_ [131], silica gel G plates [129], precoated silica gel G plates [171] and silica gel G layers impregnated with dimethylformamide [120]. Reverse phase (RP)-TLC is performed using RP-18 HPTLC plates [129] and RP-C_18_ bonded silica gel F plates [18]. The more recent TLC/HPTLC methods most commonly use HPTLC silica gel 60 F_254_ plates for successful separation of 11 phytocannabinoids (Δ^9^-THC, CBD, CBN, CBC, THCV, Δ^8^-THC, CBDV, CBG, CBGA, CBDA, Δ^9^-THCA) [127] or of Δ^9^-THC, CBD, CBN and CBG [172], or for separation of Δ^9^-THC, CBD and CBN only [125], silica gel 60 [110], silica gel 60F [135] or silica gel plates [5,126]. For some methods, for instance, the type of TLC plate was not clearly defined [130,147].

Most suitable mobile phases include xylene/hexane/diethylamine (25:10:1, *v/v/v*) [127], CHCl_3_, with plate prewashing with MeOH [110], hexane/diethyl ether (80:20, *v/v*), which allowed clear separation between Δ^8^-THC, Δ^9^-THC, CBD and CBN [125], cyclohexane/toluene/diethylether (75:15:10, *v/v/v*) [126], benzene/*n-*hexane/diethylamine (25:10:1, *v/v/v*) [130], benzene/chloroform (50:50, *v/v*) [131], diethylether/petroleum ether (1:4, *v/v*) [120], benzene/*n-*hexane/diethtylamine (25:5:0.5, *v/v/v*) [147], n-hexane/CHCl_3_/dioxane (89:8.75:2.25, *v/v/v*) [135], benzene, benzene/*n-*hexane (6:4, *v/v*), benzene/n-hexane/diethylamine (70:25:5, *v/v/v)* [129], MeOH/dioxane/hexane (1:2:7, *v/v/v*), hexane/EtAc (4:1, *v/v*), hexane/diethylether (4:1, *v/v*) [171], hexane/ethyl ether (8:2, *v/v*) [172], petroleum ether/deithylether (8:2, *v/v*), cyclohexane/diisopropyl ether/deithylamine (52:40:8, *v/v/v*) or n-hexane/dioxane/MeOH (7:2:1, *v/v/v)* [5]. For 2D TLC, first *n-*heptane/dichloromethane/butan-2-one (83:5:12, *v/v/v*) was used for first and n-hexane/acetone (86:14, *v/v*) for second development after 90° rotation [129]. RP-TLC employs MeCN:H_2_O (9:1, *v/v*) [129] or MeOH/5% acetic acid (19:1, *v/v*) [18] as mobile phase.

Evaluation of 10 mobile phases (hexane/diethylether (80:20, *v/v*), toluene, *n-*heptane/diethyl ether/formic acid (75:25:0.3, *v/v/v*), CHCl_3_, hexane/acetone (87:13, *v/v*), benzene, xylene/hexane/diethylamine (25:10:1, *v/v/v*), 4–8% diethyamine in toluene, MeOH/H_2_O with 0.1% glacial acetic acid (75:25, *v/v*) and hexane/acetone (75:25, *v/v*) on Silica gel 60 F_254_ plate showed that xylene/hexane/diethylamine (25:10:1, *v/v/v*) allows most precise bands and best separation of Δ^9^-THC, CBD and CBN, but without migration of Δ^9^-THCA, CBDA and CBGA. The cannabinoid acids were successfully separated using *n-*heptane/diethyl ether/formic acid (75:25:0.3, *v/v/v*) on C_18_ F_254_ plate and EtOH/H_2_O with 0.1% glacial acetic acid (75:25, *v/v*) on RP-C_18_ F_254_ plate [127]. Another study showed that, when using alkanes as eluents (isooctane, heptane, hexane and pentane/diethylether (90:10, *v/v*), the capability to separate Δ^9^-THC, CBD and CBN decreased as the length of the carbon-bearing chain increases [125].

Visualization of (HP)TLC plates is usually performed using 0.1% aqueous solution of Fast Blue B salt reagent [110,125,126,127,130,147], alone, under white light (254 nm and 366 nm) [127], under UV (254 nm) [131], as solution in 0.1M NaOH [5] or under UV (206 nm) [110] or as 0.5% aqueous solution, followed by 0.1M NaOH [23,125,173]. Prior to Fast Blue B, diethylamine can be applied (50 mg·L^−1^ H_2_O + 20 mL MeOH) [5]. RP-TLC plates are visualized using Fast Blue B in 0.1M NaOH or in 50 g·L^−1^H_2_O/acetone (9:1, *v/v*) [129]. Fast Blue RR was better for visualization of Δ^9^-THC, CBD, CBG and CBN than Fast Blue B salt [172]. As qualitative evaluation for the presence of cannabinoids during (HP)TLC analysis is based on color determination, it is often subject of analysts’ erroneous determination. Recent studies made the pioneering efforts in developing a method for standardizing and naming colors using the Sci-Chromus^®^ software, that significantly reduced the subjectivity of the color names in identifying Δ^9^-THC, CBD, CBN and CBG in cannabis extracts [172].

Apart from TLC and HPTLC, other planar chromatography methods are seldomly used, such as optimum performance laminar chromatography (OPLC) and automated multiple development (AMD) for phytocannabinoid profiling, despite their greater reproducibility due to complete automation. Moreover, OPLC offers extension as semipreparative technique for sample purification, while AMD offers best resolution. The only reported employment of OPLC in AMD in cannabinoid profiling is in hexane extracts of cannabis resins (dried and reconstituted in toluene) and in hexane extracts of *cannabis* resin. OPLC was performed for determination of Δ^9^-THC, CBD and CBN on HTSorb BSLA 011 and HT Sorb BSLA 003 columns using isooctane/diethylether (90:10, *v/v*) as eluent. Semi-preparative OPLC was performed for isolation of CBD from *cannabis* resin using hexane/diethylether (80:20, *v/v*). Using AMD, separation was performed on HPTLC with the elution gradient 1C acetone (100, *v/v*), diisopropylether (100, *v/v*), hexane (100, *v/v*), hexane (100, *v/v*) and hexane (100, *v/v*) during 20 steps. For both OPLC and AMD, visualisation is performed with Fast Blue B salt reagent [125].

- HPLC Methods

The HPLC technique is gaining popularity as the main choice for fingerprinting study for the quality control of herbal drugs [174], thus enabling chemical characterization of herbal medicines [175]. HPLC methods offer larger linear ranges and more consistent calibration curves for all phytocannabinoids in regards to GC-based methods [163]. In terms of reliability, reproducibility and sensitivity, it was shown that high-resolution GC/FID and HPLC-UV methods for quantification of Δ^9^-THC, CBD and CBN are comparable [98].

- HPLC Mobile Phases

Most of the mobile phases used in HPLC/DAD analysis of phytocannabinoids consisted of buffered aqueous solutions of ammonium acetate [176], ammonium formate [83,142], formic acid [11,40,50,60,61,62,63,78,173,177,178,179,180,181,182], acetic acid [19,183,184], *o*-phosphoric acid [165,185] or 5% MeCN/80% MeCN with 0.1% *o*-phosphoric acid [69]. Acidic conditions are preferred for cannabinoid acids (Δ^9^-THCA, CBDA, CBGA).

Pharmacopoeial methods (DAB, Ph.Helv., Ph.Eur.) for assay of *Cannabis flos* and *Cannabis extractum normatum* focus on the five main phytocannabinoids: CBDA, CBD, CBN, Δ^9^-THC and Δ^9^-THCA using aqueous solution of 85% *o*-phosphoric acid and MeCN as mobile phases [34,35,36,38]. AHP’s *Cannabis flos* monography recommends identical mobile phases for quantification of the major phytocannabinoids (Δ^9^-THCA, Δ^9^-THC, CBD, CBDA, CBG, CBGA and CBN) (Table S2) [33]. 

Binary mobile phase system consisted of H_2_O/MeOH (10:90, *v/v*) [186], H_2_O/MeOH (17/83, *v/v*) [187] and H_2_O + 0.1% formic acid/MeOH + 0.1%; formic acid is most commonly used for phytocannabinoid profiling [11,63,65,147,185,188]. Other binary systems that provide good peak shape and improved resolution are also used, such as H_2_O + 0.1% formic acid/MeCN + 0.1% formic acid [40,55,64,80,109,134,144,182,183,185,188,189], H_2_O + 0.1% TFA/MeOH + 0.1% TFA [68], MeCN/H_2_O (75:25, + 0.05% formic acid, *v/v*)/isopropanol:MeCN (80:20 + 0.05% formic acid, *v/v*) [183,184], 5% MeCN + 0.1% formic acid/MeCN + 5% H_2_O + 0.1% formic acid [78], H_2_O + 0.1% formic acid/MeCN [27,63,185], 5% MeCN + 0.1% formic acid/80% MeCN + 0.1% formic acid [69] and MeCN/H_2_O + 0.85% phosphoric acid [42].

Other buffering solutions are less frequently used, such as 0.1% acetic acid in a tertiary system, e.g., H_2_O + 0.1% acetic acid/MeCN + 0.1% acetic acid/MeOH [19], MeCN/0.5% acetic acid (66:34, *v/v*) [190], ammonium formate in a binary system MeOH/H_2_O + 50 mM ammonium formate (pH 5.19) [83], 5 mM ammonium formate + 0.1% HCOOH/MeCN + 0.1% HCOOH [65,100] or 5 mM ammonium formate/MeCN + 0.1% HCOOH [92].

Decrease in buffer concentration from 50 mM to 25 mM eliminated baseline drifting, thus avoiding decrease in UV absorption. As ammonium formate causes co-elution of Δ^9^-THCA with CBG, it is preferably replaced by ammonium acetate (25 mM, pH 4.75), which offers more reproducible, reliable and rugged chromatographic separation, especially between CBG and Δ^9^-THCA with improved peak shape and, thus, sensitivity. Better reproducibility is achieved using H_2_O/MeCN (15:85, *v/v*) + 50 mM phosphoric acid than with 0.1% formic acid as buffering solution [185]. Fast separation of 10 phytocannabinoids in less than 8.5 min using binary system H_2_O + 0.085% phosphoric acid/MeCN + 0.085% phosphoric acid as mobile phase was achieved, that, together with employment of RP-C_18_ column prevented co-elution of CBD and THCV and both isomers, Δ^9^-THC and Δ^8^-THC [165]. Another, less frequently used buffer is triethylammonium phosphate (TMAP) in MilliQ, with MeCN in isocratic elution programme [191].

- HPLC Columns

The multitude of LC-based methods for phytocannabinoid profiling use similar columns; it is the variation of the instrumental conditions that produces superior quantification approaches. Only one methods use direct injection [184], bypassing the column. Most of the published methods employ columns with normal phase C_18_ stationary phase (Ascentis Express C_18_ [40,178], Luna C_18_ [69,177], Kinetex C_18_ [11,19,71], Luna Omega Polar C_18_ [58,114,118,139], Luna Omega PS C_18_ [27], XTerra MS C_18_ [83], Acquity UPLC BEH C_18_ [181], Acquity BEH Shield RP18 [144], MacMod ACE5 C_18_-AR [190], ACE 3 C_18_-PFP [187], ACE Excel 3 C_18_ [111], Poroshell 120 SB-C_18_ [182], Poroshell 120 EC-C_18_ [188,189] or 120 SB-C_18_ [34,35,36,38], Shim-pack XR-ODSII RP C_18_ [149], Nucleodur^®^ C_18_ Gravity [185], Zorbax Eclipse Plus C_18_ [92,183], Zorbax Eclipse XDBC_18_ [186], Zorbax SB-C_18_ [133], Atlantis T3 C_18_ [42], with [11,19,34,35,36,38,83,188,189] or without [43,184,185,188,189,190] guard column or C_18_ guard cartridge [177] that allow reliable separation and quantification of a wide range of phytocannabinoids (focused on, but not limited to, CBDV, CBDA, CBGA, CBG, CBD, THCV, CBN, Δ^9^-THC, Δ^8^-THC, CBC, Δ^9^-THCA). Raptor ARC-18 is the C_18_ column with the widest applicability in phytocannabinoid profiling, offering the most appropriate separation of 17 phytocannabinoids (CBG, CBD, CBN, Δ^9^-THC, Δ^8^-THC, THCA, THCV, THCVA, CBC, CBCA, CBGA, CBDA, CBL, CBLA, CBDV, CBDVA, CBLA) at a runtime suitable for commercial environment. It improves peak resolution issue of some of the aforementioned columns, such as Kinetex C_18_, Luna C_18_, Luna Polar C_18_ and Raptor C_18_ [100] and has been shown to, together with Raptor ARC-18 EXP guard column, be suitable for analysis of pesticides, mycotoxins and cannabinoids [65].

The latest research points out the importance of core-shell technology columns (e.g., Poroshell) for separation of 96 phytocannabinoids by ESI-LC/MS [19]. C_18_ columns with advanced bonding of the trifunctional C_18_ phase and end-capping process, such as Acquity UPLC^®^ HSS T3, are also used [183,184]. RP-C_18_ columns, most commonly Synergi Hydro RP C_18_ [63,65,66], RP-C_18_ Hydro [61,62], Mediterranea RP-C_18_ [78], LiChrospher 60, RP-Select B [191], RP-C_18_ [165], with [60,61,62,63,191] or without [78,165] C_18_ guard column are also used. Other columns, such as Ascentis Express RP-amide are also used [64].

A better chromatographic performance (in terms of both resolution and sensitivity), a shorter analysis time (10 min vs. 12 min) and a considerable saving of solvent consumed while working at a flow rate of 0.5 mL/min instead of 1.5 mL/min, was observed while working on a fuse-core stable bond (SB) RP-C_18_ column rather than fully porous RP-C_18_ column [182]. Another study evaluated three different columns, RP-C_18_, fused-core stable bond (SB) RP-C_18_ and fused-core end-capped (EC) RP-C_18_ with numerous mobile phases and gradient conditions in an attempt to shorten the run time and to increase the separation of 8 phytocannabinoids (CBDA, CBGA, CBD, CBG, CBN, Δ^9^-THCA, Δ^9^-THC and Δ^8^-THCA). The SB RP-C_18_ core shell column provided the best performance due to significant improvement in separation and symmetry of chromatographic peaks with a baseline separation between CBD and CBG within 20 min shorter run time [176].

- HPLC Detectors

For the purpose of phytocannabinoid profiling, (U)HPLC analytical platforms are coupled to UV, DAD, PAD or MS. Phytocannabinoids have low molar absorptivity, which results in relatively low sensitivity of LC methods employing UV and DAD. This restricts employment of DAD detection to low wavelengths where there is often strong background absorbance from the eluent components, especially during gradient elution experiments [176]. Additionally, UV/DAD methods often have low specificity for some phytocannabinoids, e.g., CBDA and CBGA, due to similar UV/DAD spectra [178]. 

Phytocannabinoids have different UV behaviour on the basis of their chemical structure. Cannabinoid acids (CBDA and CBGA) are characterized by three absorption maxima (λ_max_), one stronger at 220–223 nm, the second at 266–270 nm and the third one around 305 nm, while neutral phytocannabinoids (CBD and CBG) show a first λ_max_ at 210–215 nm and an additional one at 270 nm. Generally, the ranges 190–600 nm and 200–650 nm are most commonly used for UV acquisition, while two wavelengths are selected–210 nm for neutral phytocannabinoids and 220 nm for cananbinolic acids [43,72,185]. For DAD, a narrower range is selected (i.e., 200–400 nm [83], 190–500 nm [182]). Single wavelength can be selected for evaluation of multiple phytocannabinoids in hemp seed oils [179], in plant material (230 nm) [68], 214 nm [188,189] or 220 nm in plant material and resins [69,165] or cannabis extracts [27], in commercial veterinary supplements (225 nm) [192] or for quantification of Δ^9^-THC, Δ^9^-THCA, CBN and CBD (210 nm) [191] or Δ^9^-THC and Δ^9^-THCA in plant material (211 nm and 220 nm) [185]. For wide range methods, such as cannabis-based medical extracts [182], cannabis-infused cholcolate [65] and 17 phytocannabinoids in cannabis inflorescences and oils [100], 228 nm has been shown to be the most suitable. Multiple detection wavelengths, e.g., 220 nm, 240 nm, 270 nm and 307 nm are also used for phytocannabinoids profiling [111,190]. Evaluation of these detection wavelengths for five phytocannabinoids (CBD, CBDA, CBN, Δ^9^-THC and Δ^9^-THCA) in versatile cannabis-based products was performed. While none of the phytocannabinoids showed λ_max_ at 240 nm, this wavelength tended to equalize the response (slope) across the five phytocannabinoids, except for CBN, which has the highest response. The highest response for all five components was found with 220 nm and was useful for low level quantification (Table S2). 270 nm and 307 nm provided high selectivity for CBDA and Δ^9^-THCA, which was used to minimize or eliminate detection of matrix interferences, as needed. Detection wavelengths in the range of 270–280 nm are inappropriate for CBD, CBN, and Δ^9^-THC due to retention time (R_t_) interferences [90].

Although many studies employ UV/DAD for separation of the major phytocannabinoids (CBDA, CBD, CBN, Δ^9^-THC and Δ^9^-THCA), most do not account for interference from minor phytocannabinoids (e.g, CBNA). Such interference is of special importance during profiling of concentrates where minor phytocannabinoids can be enriched to detectable levels. Additionally, some terpenes absorb UV light at the same wavelength as phytocannabinoids. All these issues decrease sensitivity, specificity and selectivity of UV/DAD methods, which are easily overcome by employment of MS detection. Detection of neutral phytocannabinoids based on their ability to absorb fluorescence under the acidic conditions used in RP-LC is only recently reported [111] as a fast, low-cost and selective alternative, but without the ability to detect phytocannabinoid acids and a somewhat narrower linear range than DAD due to saturation at high concentrations.

Most commonly used MS analyzers include simple Q spectrometers [181], QQQ analyzers [11,58,68,97,114,147,193,194], IT analyzers [28,43,72,185], QTRAP [67,159,191,195] or high resolution-accurate mass MS (HRAM-MS) analysers, such as Q-TOF [83,88,139,189] and Q-Exactive^TM^ Orbitrap [19,63,65,66].

Phytocannabinoid profiling is most commonly performed using ESI, rarely using variations such as dual source [78] or heated ESI source [19,63]. Acquisitions are performed in positive (+) [88,147,189,191,193,194], negative (−) [134] or both (+) and (−) modes [27,40,55,62,63,64,92,144,178,181]. In fact, all major phytocannabinoids are detected in both ionization modes, except for THCV [78]. However, it is noted that acidic phytocannabinoids (CBDA, THCA, CBCA, CBGA, CBNA, CBCVA) give better signals in the (−) mode, while neutral phytocannabinoids are better ionized in (+) mode (CBDV, CBG, CBD, CBC, CBDA, CBDVA, CBGA, Δ^9^-THCA, Δ^9^-THC and Δ^8^-THC) [43,58,83]. ESI (−) provides identification and quantification of additional minor phytocannabinoids, such as CBGA methyl ester (CBGMA) [40] and improves identification accuracy for two neutral phytocannabinoids (CBL and CBN) [78]. 

Atmospheric pressure chemical ionization (APCI) is also used in (+) [11] and (−) mode [69] for phytocannabinoid profiling in plant material. Formation of (+) charged sodium adducts [M+Na]^+^ instead of the precursor ion (M+ H)^+^ is observed in (+) ionization mode. This feature increases sensitivity and allows accurate identification of 7 phytocannabinoids (cannabicoumaric acid, CBCA, CBGA, CBGAM, 10-ethoxy-9-hydroxy-Δ^6^α-THC, 4-acetoxycannabichromeand Δ^9^-THCA-C4). APCI (−) is shown to be suitable for CBD, CBG and CBGA [69].

MS information from HRAM-MS acquisitions, that is, the accurate mass and mass fragmentation patterns, is used for untargeted phytocannabinoid profiling [19,63,65,66,83]. Compound identification is performed using one or more MSLs, such as mzCloud (HighChem LLC, Bratislava, Slovakia) [196], in-house MSL, such as the recent MSL of LC-MS/MS spectra of 94 phytocannabinoids accompanied with metadata (names, R_t_s, accurate masses, fragmentation patterns and fragments structures) [19], compound DBs, such as and ChemSpider [197] and Human Metabolome DB (HMDB) [198] or cheminformatics software, such as Compound Discoverer (Thermo Fischer Scientific, Waltham, MA, USA).

HPLC-UV high-resolution MS (HRMS) is employed for simultaneous quantification of the two main impurities in “pure” commercial CBG samples (cannabigerovarin (CBGV) and cannabigerobutol (CBGB)) with subsequent confirmation by comparison with synthesized compounds [193].

- Matrix Effect

The sample, i.e., matrix type greatly affects selection of extraction technique, extraction solvent(s), HPLC column, mobile phase and detection method, which further enhances method sensitivity, selectivity and specificity. All three validation parameters are affected by the presence of matrix constituents that co-extracts with phytocannabinoids, causing signal alteration (suppression or enhancement). In that spirit, matrix effect is frequently reported during LC-MS-based phytocannabinoid profiling. Cannabis plant material is complex matrix with high fat, pigment and polar compounds content, being flavonoids and terpenes most prominent. Cannabis-based products are much more versatile in terms of fat-, sugar- and polar-interferences content, thus being more prominent to expressing significant matrix suppression during instrumental analysis. 

Significant polar matrix interferences are reported to occur when EtOH is used for extraction of phytocannabinoids from honey, which also co-extracts several interfering matrix components, such as flavonoids [194]. Matrix effect is also examined during phytocannabinoid profiling of commercial products including oils, creams, and plant material. No significant matrix effect is observed in oil for Δ^9^-THC, CBD, CBDA and Δ^9^-THCA (110.4 ± 116.0%, 105.4 ± 112.2%, 96.3 ± 117.8% and 92.7 ± 107.8%, respectively); acceptable matrix effects for Δ^9^-THC and CBD in plant material (102.0 ± 112.8% and 91.2 ± 129.4%, respectively) and in creams (79.4 ± 93.1% and 83.8 ± 100.2%, respectively). Matrix effect of CBDA was especially pronounced in plant material, with signal enhancement particularly at low concentrations [173].

#### 2.2.3. SFC Methods

Despite being efficient, SFC is an analytical technique that has still not been fully exploited for the analysis, separation and quantification of cannabis plants and cannabis-based products, compared to GC and LC. The limited number of studies available report SFC as a fast (8–10 min runtime), cost-effective method with high specificity and separation power for phytocannabinoid profiling [195,199,200]. Prior to SFC analysis, derivatization and/or decarboxylation of phytocannabinoids is not required, thus reducing the risk of sample contamination (unlike GC). Additionally, SFC allows separation of the neutral from the acidic phytocannabinoids, simultaneously due to the properties of the supercritical fluids, offers shorter analysis time, better resolution and definitive identification in a single chromatogram of cannabis products when compared to both GC-MS and HPLC methods [199]. UHPSFC is shown to offer greater selectivity than UHPLC, but at the price of lower sensitivity, as a result of considerable variation of the refractive index of CO_2_, resulting in greater baseline noise [188].

Recently, the development of ultra-high performance SFC (UHPSFC) improved resolving power and efficiency, such that SFC has regained its popularity as an alternative phytocannabinoid profiling of cannabis plants and cannabis-based products. SFC combined with a 2 µm particle size column offers rapid separation and when coupled to UV or MS detection, offers highly efficient analysis of the main phytocannabinoids, using inexpensive and environmentally friendly SC-CO_2_ as solvent. On the other hand, UHPSFC is considered as highly orthogonal technique which provides different elution order and relative retention of the investigated components compared to UHPLC; therefore, in combination with MS, it could increase the discrimination power of phytocannabinoids in complex matrices. 

For the purpose of phytocannabinoid profiling, SFC using cyanopropyl silica packed column was employed [199], while the reported UHPLSFC methods used BEH 2-EP (2-ethylpyridine) column [195,200]. Evaluation of columns with different stationary phases, Torus 1-AA (1-aminoanthracene), Viridis BEH-2EP (ethyl-pyridine) and Torus Diol (OH) revealed that the latter column achieved the highest number of (although not completely separated) peaks; thus, it is most appropriate for routine phytocannabinoid profiling [188]. SC-CO_2_ is used with MeOH as co-solvent, with a constant (2%) [188] or gradually increasing concentration from 2% to 7% [199], often with the addition of 0.1% formic acid to improve peak shape of cannabinoid acids [188]. UHPSFC methods employ SC-CO_2_ with isopropanol/MeCN (80:20, *v/v*) with 1% H_2_O and linear gradient [195,200]. SFC and UHPSFC are usually coupled either to PDA detectors [188] or to mass analyzers, such as APCI-QQQ [199] and ESI-Q mass analyser [195,200]. The reported linearity, sensitivity and specificity confirm the potential of SFC and UHPSFC to become the main profiling analytical platforms instead of GC- and LC-based methods in near future.

#### 2.2.4. Vibrational Spectroscopy Methods

In the past decade, vibrational spectroscopy techniques (IR, NIR, MIR, FTIR and Raman) emerged as process analytical tool in the pharmaceutical industry for monitoring various quality attributes, and as such were recognized by United States Food and Drug Administration (FDA) [201]. Their ability for high-throughput screening of large volume of sample for a short period of time can exert sampling-based errors and provide rapid, versatile and non-invasive approach in qualitative and quantitative profiling and growth staging of cannabis plant and extracts (Table S3).

The vibrational IR and Raman spectroscopy are considered complementary techniques; even though both are relying on different physical processes, their main observations are focused on light-induced molecular excitation [202]. Vibrational spectroscopy is based on sample’s absorption of light at a defined wavelength range, which occurs as a consequence of the vibrational features of the sample that result in the formation of overtones and combination bands that form the spectrum. The relationship between the spectrum and the physicochemical properties of the sample are mathematically modelled using various multivariate regression methods (principal component analysis (PCA) and increment PCA (iPCA) [203], alone [204] or together with partial least-square (PLS) regression analysis [205,206], based on which the compound concentration in the sample is predicted.

NIR spectroscopy, in conjunction with multivariate data analysis, is a widely accepted approach for the abovementioned analysis. Quantitative data generated by NIR strongly agree with UHPLC-UV data, confirming the potential of employment of NIR spectroscopy in routine monitoring of cannabis plant material and cannabis resins [189]. Dispersive NIR and FT-NIR methods were developed for quantification of eight different phytocannabinoids (CBDV, Δ^9^-THCV, CBD, CBC, Δ^8^-THC, Δ^9^-THC, CBG and CBN) in ground leaves and inflorescences from *C. sativa* [205] and for discriminating illegal and legal cannabis varieties [207]. A similar NIR method demonstrated sensitivity and specificity for CBD quantification in different liquid pharmaceutical products, thus showcasing its potential as a fast method for monitoring of CBD in the production process [206]. Moreover, an NIR method for the growth staging of Cannabis plants was reported as being sensitive to concentrations of phytocannabinoids and volatile substances in the samples, which are also correlated to the plant age, thus justifying the feasibility of the method for growth staging of cannabis [203]. Dispersive NIR using a scanning monochromator [205], FT-NIR spectrometer with integrated Michelson interferometer and a highly sensitive PbS detector [205], FT-NIR spectrometer based on measurement by diffuse reflectance [206], NIR spectrometer with In-Ga-As detector [203], and a handheld NIR with In-Ga-As array detector [189] were so far employed in phytocannabinoid profiling in cannabis ground leaves and inflorescences [205], in cannabis seeds [203] and in liquid pharmaceutical products (medium-chain triglyceride and propylene glycol-based formulations) [206]. Two handheld NIR devices (NIR-S-GI and MicroNIR) are used for in-field determination of Δ^9^-THC content in cannabis inflorescences and cannabis resins. For this purpose, spectrophotometers with a larger sample analysis window are more appropriate for highly heterogenous samples, such as whole cannabis inflorescences [189].

The literature data regarding phytocanabinnoids’ structural and molecular analysis, to date, is very scarce, lacking band assignation and in-depth structural analysis of the molecules [23,208,209] despite the documented ability to provide chemical fingerprinting and qualitative profiling of phytocannabinoids, especially of FTIR in quantification of biological compounds in complex matrices [210]. However, the interest in qualitative profiling of cannabis (identification/classification) and quantitative profiling of the main phytocannabinoids is slowly gaining momentum in the last few years (Supporting information, Table S3). A recent study for ATR mid-IR quantification of Δ^9^-THC and CBD in cannabis flowers and extracts described a stepwise approach in developing multivariate quantification models accompanied by detailed band assignment of the mentioned phytocannabinoids, both for pure compounds and analysed samples (complex matrices) [21]. In a further study by the same group, the potential of ATR mid-IR as a process analytical tool (PAT) for continuous monitoring of Δ^9^-THCA decarboxylation was showcased [22].

The new portable vibrational spectroscopy apparatus versions are very applicable, especially for continuous monitoring of the main phytocannabinoids in all growth stages of cannabis and cannabis-based products manufacturing. Thus, a handheld Raman spectrometer in conjunction with orthogonal PLS-DA was utilized to construct a classification model for discriminating Δ^9^-THCA rich, CBD rich *Cannabis* plants and hemp (low Δ^9^-THCA, CBDA and CBD) [211,212]. Reference standards from the main phytocannabinoids were used to perform a detailed band assignation of the spectra that were further correlated with the loading plot of the multivariate models. In both cases, favourable accuracy descriptors for the classification models were reported, thus showcasing the applicability of the portable Raman device for accurate and fast *Cannabis* plant classification. Raman spectroscopy is a highly sensitive analytical technique, that, due to the variety of monochromatic light sources, and the emergence of surface-enhanced Raman scattering (SERS), stimulated resonance and coherent anti-Stokes Raman scattering (CARS) offers greatly enhanced capability and resolution, especially in its imaging mode. CARS imaging at different Raman vibrations, known as hyperspectral CARS imaging, is a spectroscopic imaging technique with high-resolution capabilities for a chemical distinction that employs sophisticated data processing methods [208]. This analytical method was used to analyze the secondary metabolites (Δ^9^-THCA and CBDA) in glandular cannabis trichomes with distinct spatial resolution, without the need to extract the resin [213]. To get additional morphological data, the authors superimposed the image over a single photon fluorescence and SEM image of the trichomes. The similarity of the chemical fingerprints of the distinct regions with the secondary metabolites was determined with hierarchical clustering analysis (HCA). The proposed methodology enables an easy discrimination between trichomes with high-Δ^9^-THCA and high-CBDA content.

#### 2.2.5. Other Analytical Techniques

- CE

CE is analytical platform used in cannabinoid profiling in only one study [68]. MeCN-based background electrolyte (with 6.5 mM NaOH) in the presence of β-cyclodextrin (βCD), improving orthogonal separation media by transiently interacting with compounds based on their geometry and polarity, was used to separate 14 phytocannabinoids (CBG, CBGA, CBD, CBDA, CBN, Δ^9^-THC, CBC, CBCA, Δ^9^-THCA, THCV, CBDV and CBGVA) by constant transition between the background electrolyte and βCD. CE performed better than HPLC-DAD in terms of selectivity and runtime, but with significantly lower sensitivity.

CE’s variety, capillary electromatography (CEC), coupling the benefits of CE and HPLC methods, has been used in phytocannabinoid profiling coupled with UV PDA detector in only one study [214]. Baseline separation of seven phytocannabinoids (CBG, CBD, CBN, Δ^9^-THC, Δ^8^-THC, CBC, Δ^9^-THCA) was achieved by using C_18_ column and MeCN/25 mM phosphate buffer (75:25, *v/v*) as mobile phase for analysis of MeOH/CHCl_3_ (9:1, *v/v*) *cannabis* plant material extracts, while improved sensitivity is achieved using UV cell with extended path length and injection size.

- NMR Spectroscopy

NMR-based methods are superior for the purpose of 3D-structure elucidation, especially ζ-resolving, ^1^H-^1^H COSY and ^1^H-^13^C heteronuclear multiple quantum coherence (HMQC) and heteronuclear multiple bond correlation (HMBC) spectroscopy. The complete ^1^H- and ^13^C-NMR assignments of the major *Cannabis* constituents, ∆^9^-THC, Δ^9^-THCA, ∆^8^-THC, CBG, CBN, CBD, CBDA, cannflavin A and cannflavin B have been determined on the basis of one- and two-dimensional NMR spectra, including ^1^H- and ^13^C-NMR, ^1^H-^1^H-COSY, HMQC and HMBC [20]. However, they are seldomly used for quantification purposes, due to the laborious separation and isolation steps required, where significant loss of mass can occur and where there is low sensitivity [4,215]. Additional issues are the high instrumental costs and necessity of highly specialized personnel. Despite that, NMR is considered as a highly accurate, reproducible and fast technique [1]. 

For the purpose of phytocannabinoid profiling, NMR spectroscopy is used as (semi)quantitative method alone [20,209] or as an orthogonal technique to LC [216,217,218] or GC [219] for the purpose of qualitative peak assignment of major phytocannabinoids [20], chemical and morphological examination [220], chemotaxonomic classification [20,219,220], metabolomics-based chemovar distinction [51] or quantitative analysis of *cannabis* plant material without the need of pre-purification step [221], chromatographic separation or use of certified reference standards [219]. Cryogenic NMR spectroscopy combines improved sensitivity and noise reduction with a cryogenic cooling system for the receiver coil and preamplifiers. Its improved spectral quality is employed in compound identification from mass limited samples and as orthogonal analytical technique to HPLC in phytocannabinoid profiling in laser-micro dissected samples of capitate-stalked and capitate-sessile trichomes [220].

## 3. Conclusions and Future Directions

Scientific and technological advancements in cultivation, manufacturing, recreational, industrial and medical use of cannabis, as well as updated legislation, led to the development of multitude of analytical methods for phytocannabinoid profiling. Matrix nature greatly affects selection of extraction technique, sample preparation and analytical method due to the fact that significant matrix interferences can occur and aggravate the overall analysis of target phytocannabinoids. Sample preparation for phytocannabinoid profiling in the past four decades is mainly based on versatile types of accelerated maceration, such as SLE, LLE, PLE, SPE, USE, FUSE and MAHD. Recently, new trends have enlightened environmental-friendly techniques, such as easily-automatable HS-SPME and SFE, which adds speed, repeatability and reproducibility to the analyses. From the multitude of analytical platforms, TLC and HPTLC, HPLC-DAD, GC and LC coupled with mass spectrometry (MS or MS/MS), are most commonly used; however, recently emerging techniques are NMR and vibrational spectroscopy methods, such as IR, NIR, FTIR and FT-NIR. TLC, together with HPTLC, which is a suitable method for screening of samples and is included in the pharmacopoeias in the identification methods. Cannabinoid profiling for research, industrial and QC purposes is based mostly on two analytical platforms: GC and LC. GC coupled to versatile detectors and mass analyzers is one of the oldest, the most preferred and researched analytical platforms for phytocannabinoid profiling due to its robustness, reproducibility, sensitivity and speed.

As a result of the advancement of computational tools, mass spectral libraries (MSLs), public compound repositories and compound databases (DBs), as well as various advanced detection techniques, GC has become the analytical platform in forensic, pharmacokinetic and phytochemical analysis of natural phytocannabinoids. As such, GC methods are also officially employed by authorities for terpene profiling, pesticide screening and residual solvents analysis, which affords potential benefits to regulatory bodies and cannabis industry. High pressure and high flow rates used during TLC, HPTLC, HPLC and UHPLC, and the recently emerging SFC technique allow sample preservation without decarboxylation and decomposition, reliable separation of neutral and acidic cannabinoid species and, thus, direct identification and quantification of both neutral and acidic forms of phytocannabinoids in the extracted samples. In the past decades, LC has become an analytical platform of choice (HPLC-DAD and LC-MS) in first line for potency studies but also for untargeted analysis of cannabis and cannabis-based products. Despite the fact that vibrational spectroscopy methods, such as NIR, FTIR, FT-NIR and Raman spectroscopy are reserved for structural elucidation, in the last few years there is an evident trend of their utilization for rapid quantitative phytocannabinoid profiling. Although these vibrational spectroscopy techniques can provide rapid, versatile and non-invasive approach in qualitative and quantitative profiling and growth staging of cannabis plant and extracts, they demonstrate far higher LOD and LOQ than the described chromatography-‘wet’ methods, they are fast, inexpensive, non-destructive and require minimum (e.g., drying, grinding) or no sample preparation. Regardless of high instrumental costs and necessity of highly specialized personnel, NMR is considered as a highly accurate, reproducible and fast technique that offers quantitative analysis of cannabis without the need of pre-purification step, chromatographic separation or use of certified reference standards. Even though there are currently various well-established methods available for chemical analyses of phytocannabinoids, there is still a need for adaptations and enhancement of these methods in the light of new scientific evidence regarding the plant and its plant metabolites, especially taking into account the pharmacological activity and its medical use, association of medical and adverse health effects with potency and/or interplay of certain phytocannabinoids and other active constituents, quality control and stability studies of cannabis and cannabis-based products. Further advancements in phytocannabinoid profiling should move towards untargeted analysis of cannabis plant material and cannabis-based products using orthogonal analytical methods. By employment of cheminformatics approaches for small molecule identification and MSLs, a multitude of new phytocannabinoids and other compounds is expected to be identified in the near future, thus allowing access to complete and accurate phytocannabinoid and terpene profiles.

## Figures and Tables

**Table 1:** Formula, MS and UV data of major phytocannabinoids.

Compound [18]	Molecular Formula and Mr [18]	[M-H]^−^ [MF1-H]^−^ [MF2-H]^−^ [MF3-H]^−^ [19]	Structure [19]	UV-VIS Spectra [18] Acidic HPLC Systems/ Basic HPLC Systems	^1^H NMR in Deuterated Chloroform [4,20]
Δ^9^-THC	C_21_H_30_O_2_ 314.472	C_21_H_29_O_2,_ 313.2173 C_16_H_21_O_2,_ 245.1547 C_12_H_15_O_2,_ 191.1078 C_11_H_15_O_22,_ 179.1067	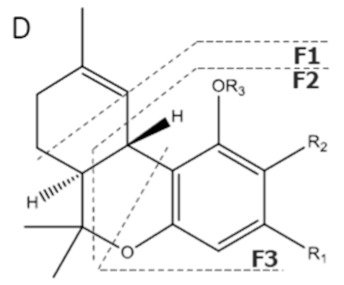 R_1_-C_5_H_11_, R_2_-H, R_3_-H	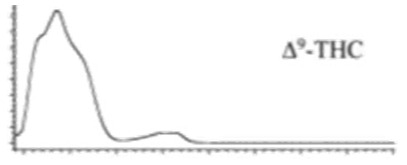 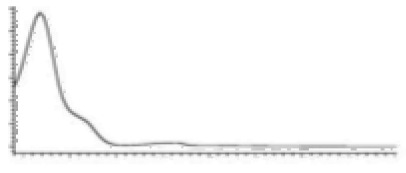	3.20 (1H, dm, 10.9 Hz) 6.31 (1H, q, 1.6 Hz) 1.68 (3H, s) 2.16 (2H, m) 1.90 (1H, m), 1.40 (m) 1.69 (m) 1.41 (3H, s) 1.09 (3H, s) 6.14 (1H, d, 1.6Hz) 6.27 (1H, d, 1.6 Hz) 2.42 (2H, t, 7.3 Hz, 1.6 Hz), 1.55 (2H, q, 7.8 Hz) 1.29 (m) 1.29 (m) d 0.87 (3H, t, 7.0 Hz) 4.87 (1H, s)
Δ^8^-THC	C_21_H_30_O_2_ 314.472	C_21_H_29_O_2,_ 313.2173 C_16_H_21_O_2,_ 245.1547 C_12_H_15_O_2,_ 191.1078 C_11_H_15_O_2,_ 179.1067	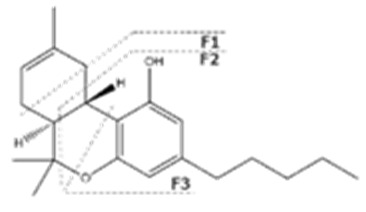 -	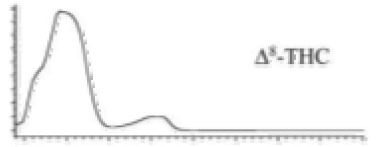 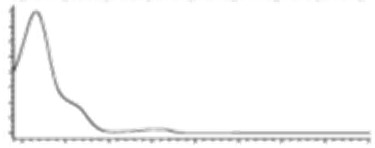	
THV	C_19_H_26_O_2_ 286.418	C_19_H_25_O_2,_ 285.1860 C_14_H_17_O_2,_ 217.1234 C_10_H_11_O_2,_ 163.0765 C_9_H_11_O_2,_ 151.0765	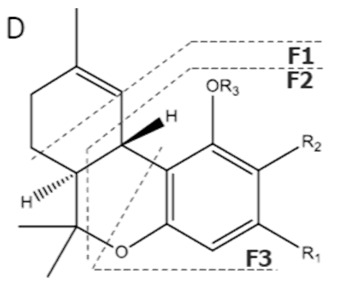 R_1_-C_3_H_17_, R_2_-H, R_3_-H	- -	
CBD	C_21_H_30_O_2_ 314.472	C_21_H_29_O_2,_ 313.2173 C_16_H_21_O_2,_ 245.1547 C_12_H_15_O_2,_ 191.1078 C_11_H_15_O_2,_ 179.1067	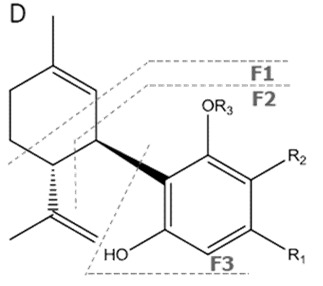 R_1_-C_5_H_11_, R_2_-H, R_3_-H	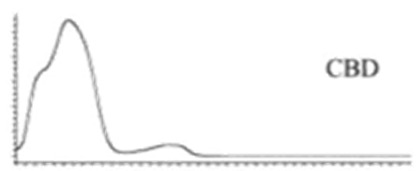 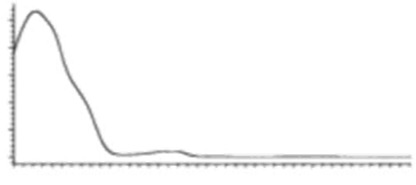	3.90 (1H, dm, 11.8Hz) 5.57 (1H, s) 2.21 (1H, m), 2.09 (1H, m) 1.84 (m) 2.40 (m) 1.79 (3H, s) 4.64 (trans, 1H, m), 4.54 (cis, 1H, m) 1.66 (3H, s) 6.26 (1H, brs) 6.16 (1H, brs) 2.43 (2H, t, 7.5Hz) 1.55 (2H, q, 7.6Hz) 1.29 (m) 1.29 (m) 0.88 (3H, t, 6.8Hz) 5.99 (1H, s) 5.02 (1H, s)
CBN	C_21_H_26_O_2_ 310.440	C_21_H_25_O_2,_ 309.1860 C_19_H_19_O_2,_ 279.1391 C_12_H_11_O_2,_ 171.0815	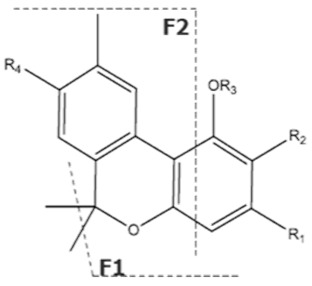 R_1_-C_5_H_11_, R_2_-H, R_3_-H, R_4_-H	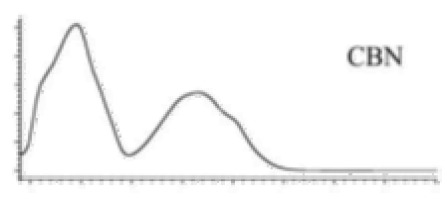 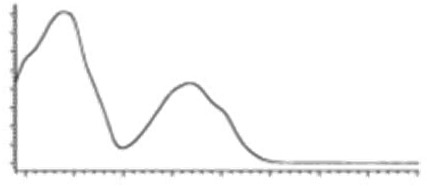	8.16 (1H, s) 2.38 (3H, s) 7.07 (1H, d, 7.9Hz) 7.14 (1H, d, 7.9Hz) 1.60 (6H, s) 1.60 (6H, s) 6.29 (1H, d, 1.1Hz) 6.44 (1H, d, 1.1Hz) 2.50 (2H, t, 7.5Hz) 1.63 (m) 1.32 (m) g 1.32 (m) g 0.89 (3H, t, 6.8Hz)5.13 (1H, s)
CBG	C_21_H_32_O_2_ 316.488	C_21_H_31_O_2,_ 315.2329 C_16_H_21_O_2,_ 245.1547 C_12_H_15_O_2,_ 191.1078 C_11_H_15_O_2,_ 179.1067	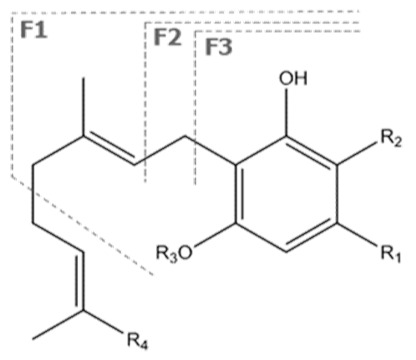 R_1_-C_5_H_11_, R_2_-H, R_3_-H, R_4_-H	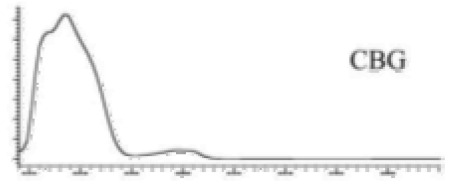 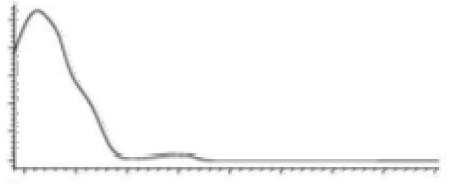	6.26 (2H, s) d 6. (2H, s) d 3.41 (2H, d, 7.0 Hz) 5.29 (1H, m) 1.82 (3H, s) 2.09 (4H, m) 2.09 (4H, m) 5.07 (1H, m) 1.60 (3H, s) 1.69 (3H, s) 2.45 (2H, t, 7.5 Hz) 1.56 (2H, q, 7.8 Hz) 1.33 (4H, m) 1.33 (4H, m) 0.90 (3H, t, 6.9 Hz) 5.36 (2H, s)
CBC	C_21_H_30_O_2_ 314.172	C_12_H_29_O_2,_ 313.2173 C_16_H_19_O_2,_ 243.1391 C_12_H_15_O_2,_ 191.1078 C_11_H_15_O_2,_ 179.1067	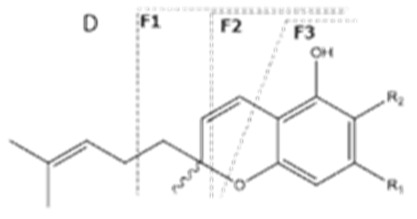 R_1_-C_5_H_11_, R_2_-H	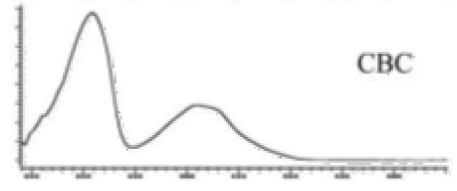 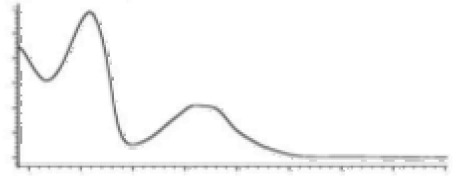	N/A
CBL	C_21_H_30_O_2_ 314.472	C_21_H_29_O_2,_ 313.2173 C_16_H_19_O_2,_ 243.1391 C_12_H_15_O_2,_ 191.1078 C_11_H_15_O_2,_ 179.1067	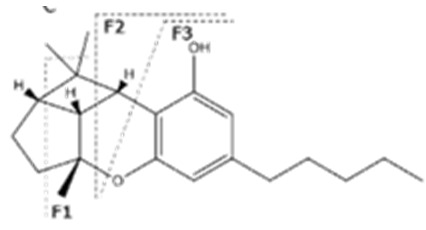	N/A	N/A
Δ^9^-THCA	C_22_H_30_O_24_ 358.482	C_22_H_29_O_4,_ 357.2071 C_21_H_30_O_2,_ 245.1547 C_12_H_15_O_2,_ 191.1078 C_11_H_15_O_22,_ 179.1067	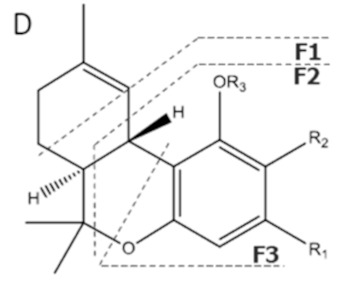 R_1_-C_5_H_11_, R_2_-COOH, R_3_-H	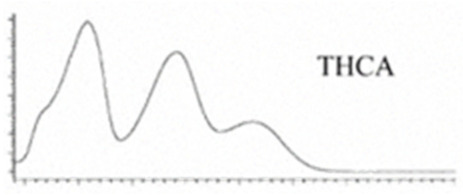 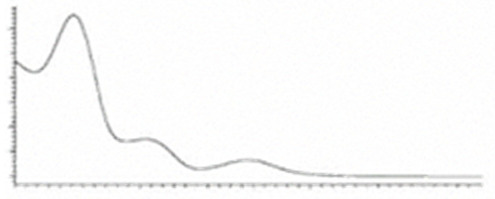	3.23 (1H, dm, 7.0 Hz), 6.39(1H, brs), 1.68 (3H, s), 2.17 (2H, m) 1.92 (1H, m) 1.35 (m) 1.67 (m) 1.44 (3H, s) 1.11 (3H, s) 6.26 (1H, s) 2.94 (1H, m) 2.78 (1H, m) 1.57 (2H, 1.35 (m) 1.35 (m) 0.90 (3H, t, 6.9 Hz) 12.19 (1H, s)
Δ^9^-THCA-C4	C_21_H_28_O_4_ 344.455	C_21_H_27_O_4,_ 343.1915 C_15_H_19_O_2,_ 231.1391 C_11_H_13_O_2,_ 177.0921 C_10_H_13_O_2,_ 165.0921	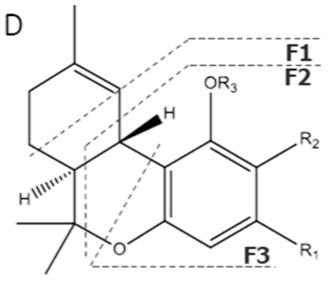 R_1_-C_4_H_9_, R_2_-COOH, R_3_-H	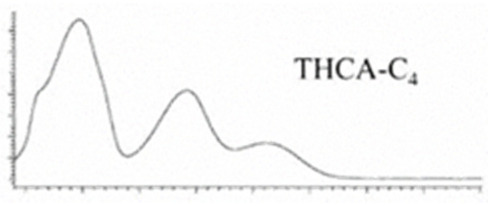 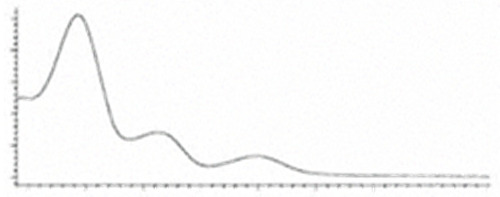	N/A
THVA	C_20_H_26_O_4_ 330.428	C_20_H_25_O_4,_ 329.1758 C_14_H_17_O_2,_ 217.1234 C_10_H_11_O_2,_ 163.0765 C_9_H_11_O_2,_ 151.0765	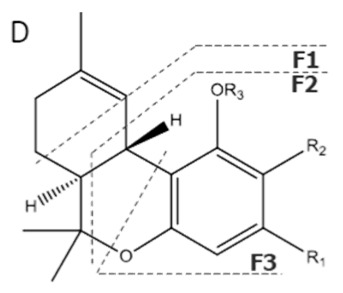 R_1_-C_3_H_7_, R_2_-COOH, R_3_-H	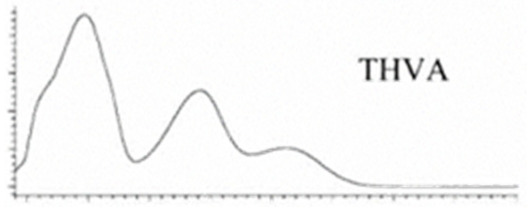 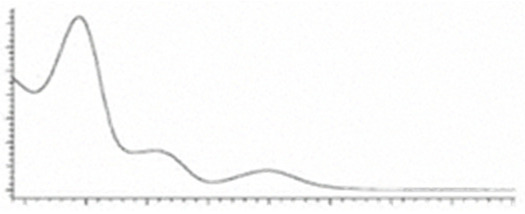	N/A
CBDA	C_22_H_30_O_4_ 358.482	C_22_H_29_O_4,_ 357.2071 C_16_H_21_0_2,_ 245.1547 C_12_H_15_0_2,_ 191.1078 C_11_H_15_0_2,_ 179.1067	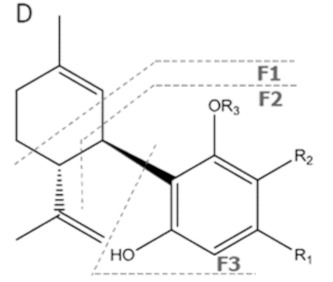 R_1_-C_5_H_11_, R_2_-COOH, R_3_-H	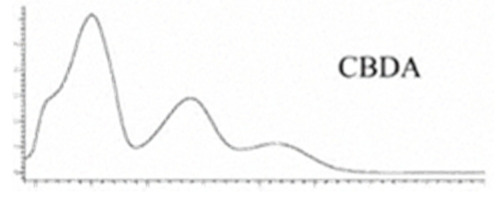 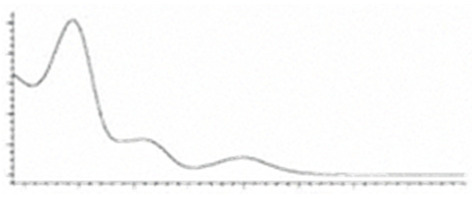	N/A
CBNA	C_22_H_26_O_4_ 354.450	C_22_H_25_O_4,_ 353.175 C_19_H_19_0_2,_ 279.1391 C_12_H_11_0_2,_ 171.0815 C_21_H_25_0_2,_ 309.1860	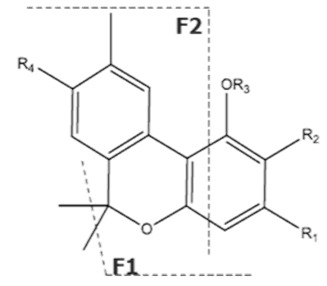 R_1_-C_5_H_11_, R_2_-COOH, R_3_-H, R_4_-H	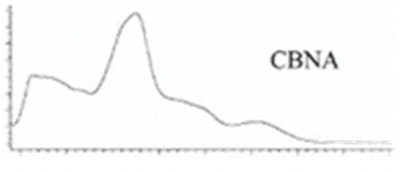 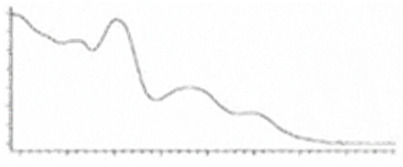	N/A
CBGA	C_22_H_32_O_4_ 360.498	C_22_H_31_O_4,_ 359.2228 C_16_H_21_O_2,_ 245.1547 C_12_H_15_O_2,_ 191.1078 C_11_H_15_O_2,_ 179.1067	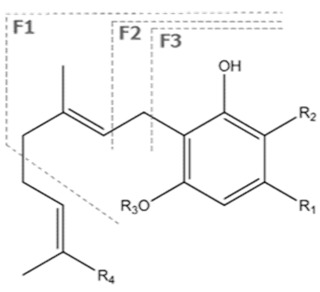 R_1_-C_5_H_11_, R_2_-COOH, R_3_-H, R_4_-H	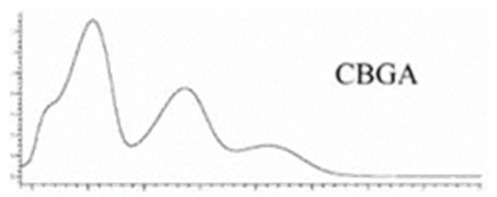 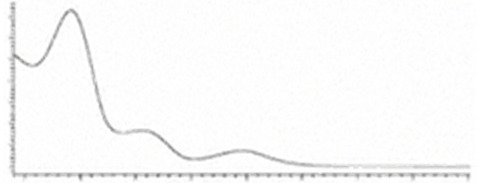	N/A
CBCA	C_22_H_30_O_4_ 358.482	C_22_H_29_O_4,_ 357.2071 C_16_H_19_O_2,_ 243.1391 C_12_H_15_O_2,_ 191.1078 C_11_H_15_O_2,_ 179.1067	R_1_-C_5_H_11_, R_2_-COOH, 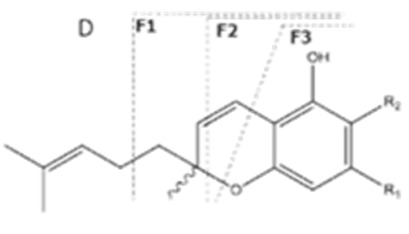	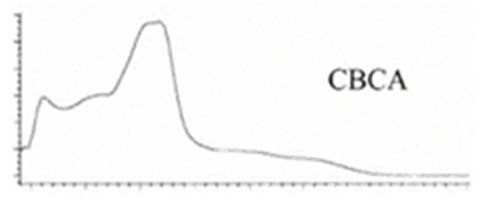 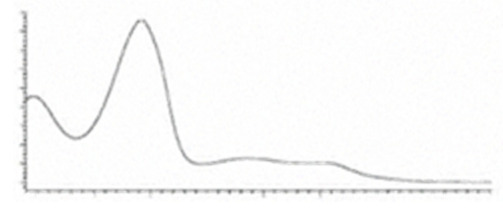	N/A
CBLA	C_22_H_30_O_4_ 358.482	- - - -	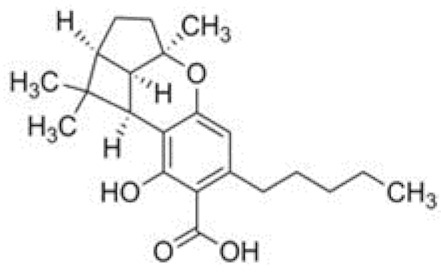	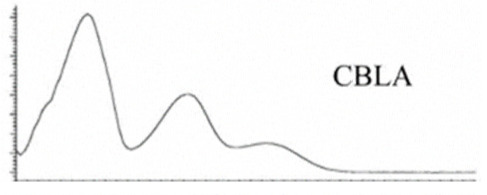 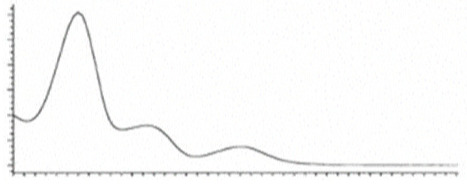	N/A

**Table 2:** Sample preparation techniques for phytocannabinoid profiling of cannabis and cannabis-based products.

Sample Preparation Technique	Advantages	Disadvantages
LLE	- variety of solvents and solvent mixtures with appropriate extraction efficiencies; - appropriate for all matrices - low price	- high solvent consumption
PLE	- possibility to perform decarboxylation in situ - greatreproducibility - low price	- miscelanous scientific finidings regarding the ability of PLE to extract thermolabile compounds
HS-SPME	- programmable automated operation; - improved chromatographic peak shape; - reduction of matrix interferences	- specific to GC-based methods only; - applicable mostly for simple matrices (herbal material)
SFE	- “green” extraction method; - ensures stability of thermolabile and light-sensitive phytocannabinoids; - high extraction yields; - ability to separate phytocannabinoids from terpenes	- rarely used - high price
FUSE, UAE	- low solvent and energy consumption	- applicable mostly for simple matrices (herbal material)
SPE	- most suitable for food matrices and extracts - “green” extraction technique	- laborious and time-consuming - high price
MHD	- simultaneous extraction of terpenes and phytocannabinoids - simultaneous decarboxylation	- more commonly used for extraction of essential oils - high price
CPE	- analyte extraction and preconcentration in a single, solvent-free step - avoidance of analyte loss during solvent evaporation - low price	- low extraction efficiency for phytocannabinoids - time consuming
CPC	- allows for large-scale extraction of phytocannabinoids with high efficiency	- high solvent consumption and waste generation - high price

**Table 3:** Most commonly used solvents and solvent mixtures in maceration and LLE of phytocannabinoids.

Solvent/Solvent Mixture	References
MeCN	[89,90]
MeCN + 1% acetic acid	[65]
MeCN saturated with n-hexane	[91]
MeOH	[38,42,50,54,56,68,78,79,85,89,92,93,94,95,96,97,98,99,100,101]
absolute ethanol (99.7%, *v/v*)	[10,49,51,53,66,90,102,103,104,105,106,107,108]
EtOH(96%, *v/v*)	[40,54,55,66,90,92,100,109,110,111,112,113]
isopropanol	[63,108]
cyclohexane	[82,114]
EtAc	[69,89,115,116,117]
CHCl_3_	[44,52,58,77,118,119,120]
*n*-hexane	[40,47,54,66,75,76,78,86,87,101,121,122,123,124,125]
light petroleum	[46]
petroleum ether	[126,127,128]
toluene	[129]
benzene	[130]
CCl_4_ (later evaporated and extracts reconstituted in chloroform)	[131]
MeCN/MeOH (8:2, *v/v*)	[132]
hexane/isopropanol (9:1, *v/v*)	[57,94,106,133]
hexane/EtAc (9:1, *v/v*), (7:3, *v/v*), (6:4, *v/v*)	[54,57,66,94,104]
hexane/CHCl_3_ (1:1, *v/v*)	[134,135]
MeOH/CHCl_3_ (4:1, *v/v*)	[48,136]
MeOH/CHCl_3_ (9:1, *v/v*), (99:1, *v/v*)	[57,67,86,106,137]
MeOH/hexane (9:1, *v/v*)	[138]
petroleum ether/MeOH (9:1, *v/v*)	[45]
EtOH/H_2_O (1:1, *v/v*)	[133]
KOH in MeOH and hexane/EtAc (9:1, *v/v*)	[139]
IS (tribenzylamine) in 96% EtOH	[57]
IS (tribenzylamine) in MeCN	[140]
IS (nonadecane) in EtOH	[138]
IS (diphenylhydramine) in EtOH	[74]
IS (4-androstene-3,17-dione) in EtOH	[9,137]
IS (docosane) in petroleum ether	[128,141]
IS (nonadecane) in MeOH/CHCl_3_ (9:1, *v/v*)	[67]
IS (squalane) in hexane	[76,89,142]
IS (chrysene-d_12_) in hexane	[71]
IS (ketamine hydrochloride) in MeCN	[124]
IS (4-androstene-3,17-dione) in MeOH/CHCl_3_ (9:1, *v/v*)	[9,75,86,87,143,144,145]

**Table 4:** Analytical techniques for phytocannabinoid profiling of cannabis and cannabis-based product.

Analytical Techniques	Advantages	Disadvantages	Note
GC-FID	- More accurate cannabinoid quantification than GCMS - Terpenes and residual solvents - High resolution	- Time-consuming derivatization for acidic cannabinoids	- Gold standard technique for forensic purposes
GC-MSD	- -Compound libraries to identify the parent analyte - Higher specificity - Sensitive	- Use of equivalent deuterated standards (expensive and not available for all cannabinoids)	/
GC-QQQ/QTOF	- -Simultaneous analysis of cannabinoids, terpenes and residues of pesticides - Highest sensitivity - Analysis of “Unknowns”	/	/
(HP)TLC	- Rapid screening of many samples to confirm the existence of cannabinoids, provide better resolution and generate reports for more convenient documentation for peer review of casework in crime labs	- Lower performance compared to other separation techniques - Reproducibility depends of humidity	- Compulsory method for identification
HPLC-UV/DAD	- Quantification of both acidic and neutral forms of phytocannabinoids	- The complex composition of the cannabis material leads to significant peak overlap of the phytocannabinoids - Only target analytes can be determined, not full spectrum - Limited use for analyses of biological samples the complex composition of the cannabis material leads to significant peak overlap of the phytocannabinoids - Only target analytes can be determined, not full spectrum - Limited use for analyses of biological samples	/
HPLC-QQQ	- Fingerprinting with excellent sensitivity and selectivity of complex matrices	- Set-up of QQQ instruments require careful tuning and optimization (require time and effort)	- Often are used for simultaneous pesticide and mycotoxins/aflatoxins analysis
HPLC-Q-Exactive Orbitrap^®^	- High selectivity of complex matrices - Confirm analyte structure - Analysis of “Un-knowns”		
SFC	- Green technique suitable for thermally labile compounds	- Availability of SFC equipment	/
NMR	- Not sensitive to ballast compounds (chlorophylls and lipids) reference standards are not required	- High cost of this analyser	/
RAMAN	- Rapid, versatile and non-invasive qualitative and quantitative profiling growth staging of cannabis plant and extracts	/	/
FTIR, NIR, MIR	- Chemically fingerprint substances - Analysis of heterogeneous substances like cannabis samples and to determine the potency of cannabis flower - Rapid on-site use for monitoring growth and curing processes of cannabis	- Should be combined with chemometrics - Less accurate for potency analyses	/

## Supplementary Materials

The following are available online. Table S1: GC-based analytical methods for cannabinoid profiling [5,9,10,17,18,43,44,45,46,47,48,49,51,52,53,54,55,56,57,58,59,66,67,70,71,72,73,74,75,77,78,79,81,82,84,85,86,87,88,89,90,91,93,94,95,96,97,98,99,101,102,103,104,105,106,107,108,114,115,116,118,119,121,122,123,124,128,132,136,137,138,139,140,141,148,150,152,153,154,155,156,157,158,160,161,163,166,216,222,223,224]; Table S2: LC-based analytical methods for cannabinoid profiling [11,19,27,40,42,50,55,60,61,62,63,64,65,68,69,78,80,83,92,100,107,109,111,113,133,134,142,144,149,165,173,176,177,178,179,180,182,183,185,186,187,188,190,191,192,194,200,215,217]; Table S3: Vibrational spectroscopy-based analytical methods in conjunction with multivariate data analysis for phytocannabinoid profiling and/or classification of cannabis plant material [21,189,203,204,205,206,207,211,212,218].

## Sample Availability

Samples of the compounds are available from the authors.
