# Supplementary material for: Analytical Techniques for Phytocannabinoid Profiling of Cannabis and Cannabis-Based Products—A Comprehensive Review
Source: Molecules. 2022 Feb 1;27(3):975. doi: 10.3390/molecules27030975 (PMC8838193; doi:10.3390/molecules27030975)
Supplement: Supplementary file 1 [file molecules-27-00975-s001.zip › molecules-1502098-supplementary.pdf]

Analytical Techniques for Phytocannabinoid Profiling of Cannabis and Cannabis-Based Products–A Comprehensive Review

Supplementary Material

Table S1. GC-based analytical methods for cannabinoid profiling, COTP - column oven temperature program, IV- injection volume, LOD - limit of detection, LOQ – limit of quantification.

**Matrix/amount**	**Derivatization conditions (derivatization agent amount, temperature, time)**	**instrument type /column**	**GC and detector conditions (COTP and other *t*)**	**compound identification**	**runtime (min)**	**LOD/*LOQ* (ngmL^{-1} or ng/g), * -LOD/*LOQ* expressed in % *(w/w)***	**Year, country**	**Ref.**
**grinded herbal cannabis / 0.1 g**	-	GC + FID	COTP: 260°C, isothermal<br>injector: 300°C<br>FID: 300°C<br>IV: 1 μL	THC (Δ^9-THC + Δ^9-THCA)	-	-	2017,-	[1]
	-	Shimadzu GC-2010 + FID<br>Rxi-5MS (30 m x 0.25 mm x 0.25 μm)	COTP: 260°C (10 min), 20°Cmin^{-1} to 300°C (2 min)<br>injector: 300°C<br>split mode (1:40)<br>FID: 300°C<br>IV: 1 μL	THC (Δ^9-THC + Δ^9-THCA)	14.00	-	2021, Italy	[2]
		Thermo Focus GC/DSQ II<br>Rxi-5MS (30 m x 0.25 mm x 0.25 μm)	COTP: 260°C (10 min), 20°Cmin^{-1} to 300°C (2 min)<br>injector: 300°C<br>split mode (1:40)<br>ion source: 270°C<br>interface: 250°C<br>IV: 1 μL	EI, 70 eV<br>full scan mode<br>(*m/z* 40-450)<br>THC (Δ^9-THC + Δ^9-THCA)				

Matrix/amount	Derivatization conditions (derivatization agent amount, temperature, time)	instrument type /column	GC and detector conditions (COTP and other t)	compound identification	runtime (min)	LOD/*LOQ* (ngmL^{-1} or ng/g), * -LOD/*LOQ* expressed in % *(w/w)*	Year, country	Ref.
**traditional cannabis / sinsemilla / cannabis resin / 0.25 g**	-	Agilent 7890 + FID<br>Zebron ZB-5HT Inferno (30 m x 0.32 mm x 0.25 μm)	COTP: 60°C (3 min), 35°Cmin^{-1} to 200°C, 5 °Cmin^{-1} to 250°C (5 min), 100°Cmin^{-1} to 320°C (3.3 min)	Δ^{9}-THC, CBD, CBN	25.00	-	2015, England	[3]
**herbal cannabis / cannabis resin / marijuana joint / 0.05-0.10 g**	-	HP 5890 + FID<br>CPsil8CB (25 m x 0.32 mm x 0.25 μm)	COTP: 250°C, isothermal<br>injector: 280°C<br>split mode (20 mLmin^{-1})<br>FID: 300°C	Δ^{9}-THC, CBD, CBN	8.00	Δ^{9}-THC 0.10<br>CBD 0.10<br>CBN 0.10	2000-2004<br>2013<br>2014<br>2005-2015	[4]*<br>[5]*<br>[6]*<br>[7]*
**dried / herbal cannabis / resin / other (mainly homemade cigarettes with cannabis material) / 0.05-0.1 g**	-	Agilent 7890 + FID<br>HP-5MS (12 m x 0.20 mm x 0.33 μm)	COTP: 220°C (1 min), 6°Cmin^{-1} to 250°C (2 min)<br>injector: 250°C<br>split mode (1:100)<br>FID: 300°C	Δ^{9}-THC, CBD, CBN	8.00	-	2010-2012, northern Italy<br>2013, northern Italy	[8]<br>[9]
**inflorescences of pistillated plants / 0.05 g**	-	HP 5710A GLC + FID<br>DB-5MS (30 m x 0.53 mm x 1.5 μm)	COTP: 200°C (8 min), 4°Cmin^{-1} to 300°C (4 min)<br>injector: 250°C<br>FID: 250°C	Δ^{9}-THC, CBG, CBGM, CBDV, THCV, CBD, CBC	37.00	-	2004, USA	[10]

Matrix/amount	Derivatization conditions (derivatization agent amount, temperature, time)	instrument type /column	GC and detector conditions (COTP and other t)	compound identification	runtime (min)	LOD/*LOQ* (ngmL^{-1} or ng/g), * -LOD/*LOQ* expressed in % *(w/w)*	Year, country	Ref.
**cannabis leaves/ cannabis oil / cannabis resins / 0.005 – 0.03 g**	-	GC + FID<br>DB-1 (10 m x 0.18 mm x 0.4 μm)	COTP: 200°C (4 min), 15°Cmin^{-1} to 280°C (2 min)<br>injector: 250°C<br>split mode (22.2 mLmin^{-1})<br>FID: 280°C	Δ^9-THC, CBD	11.33	-	1976-1995, New Zealand	[11]
**whole cannabis plants / 1.0 g**	dry extracts in 500 μL pyridine + 500 μL BSTFA + 1% TMCS<br>60°C, 5 min	Beckman GC-4 and GC-45 + FID<br>2% OV-17<br>100-200 mesh GasChrom Q	FID: 210°C	Δ^9-THC, Δ^9-THCA	52.00	-	1971, Mexico	[12]
**whole mature cannabis plants / cannabis resin / 1.0 g**	-	Pye 104 + FID<br>3% OV-17 100-200 mesh GasChrom Q (1.5 m x 4 mm)	COTP: 250°C, isothermal<br>IV: 5 μL<br>FID: 300°C	Δ^9-THC, Δ^9-THCA	-	-	1975-1978, United Kingdom	[13]
**whole mature cannabis plants / 2.0 g**	-	PU4500 + FID<br>3% OV-17 100-120 mesh Chromosorb WHP (1.5 m x 4 mm)	COTP: 245°C, isothermal	total THC (Δ^9-THC + THCA)	-	-	1984-1989, United Kingdom	[14]

Matrix/amount	Derivatization conditions (derivatization agent amount, temperature, time)	instrument type /column	GC and detector conditions (COTP and other t)	compound identification	runtime (min)	LOD/*LOQ* (ngmL^{-1} or ng/g), * -LOD/*LOQ* expressed in % *(w/w)*	Year, country	Ref.
**dried cannabis leaves and flowering/fruiting tops / 5.0 g**	-	HP 5590 + FID<br>SPB-1 (15 m x 0.53 μm x 1.5μm)	COTP: 60°C (1.5 min), 30°Cmin^{-1} to 300°C (8 min)<br>injector: 275°C<br>splitless mode<br>FID: 350°C	Δ^{8}-THC, Δ^{9}-THC, CBD, CBG, CBN	17.50	-	1988, Denmark	[15]
**dried and pulverised cannabis plant material / cannabis flowers / cannabis resin / 0.05 -0.10 g**	-	Carlo Erba GC8000 Top + FID<br>DB-5MS (15 m x 0.25 mm x 0.25 μm)	COTP: 120°C (2 min), 20C min^{-1} to 300°C (3 min)	Δ^{9}-THC	14.00	-	2004, Switzerland	[16]
**upper main stem of flowering cannabis plants, powedered / 0.02 g**	-	HP GCD + MSD<br>HP-5MS (30 m x 0.25 mm x 0.25 μm)	COTP: 100°C, 15°Cmin^{-1} to 300°C (8 min)<br>injector: 280°C<br>splitless mode<br>interface: 300°C	EI, 70 eV<br>SIM mode<br>CBD (**231**, 174, 314)<br>CBN (**295**, 238, 310)<br>Δ^{9}-THC (**299**, 314, 231)	21.33	-	1996, Greece<br>1999, Greece	[17]<br>[18]
**dried cannabis upper flowering plant parts, powdered / 0.1 g** [19]<br>**cannabis leaves / reproductive parts / calli / 0.1 g** [20]	-	GC 8000 Top + FID<br>fused silica capillary column (30 m x 0.32 mm x 0.5 μm)	COTP: 220°C-300°C<br>injector: 300°C<br>splitless mode<br>FID: 300°C	Δ^{9}-THC, CBD, CBG		-	2006, Italy<br>2008, Italy	[19]<br>[20]

Matrix/amount	Derivatization conditions (derivatization agent amount, temperature, time)	instrument type /column	GC and detector conditions (COTP and other t)	compound identification	runtime (min)	LOD/*LOQ* (ngmL^{-1} or ng/g), * -LOD/*LOQ* expressed in % *(w/w)*	Year, country	Ref.
**dried cannabis from illegal farm / dried seized herbal cannabis / dried cannabis from authorized trial fields / 1.0 g**<br><br>**cannabis leaves / 0.5 g**	-	Chrompack 9002 GC + FID<br>DB-1 (30 m x 0.32 mm x 0.25 μm)	COTP: 230°C (7 min), 10°Cmin^{-1} to 260°C (2 min)<br>IV: 1 μL<br>injector: 260°C<br>split mode (1:20)<br>FID: 270°C	Δ^9-THC, CBD, CBN	12.00	-	2008, Northern Thailand<br><br>2009, Northern Thailand	[21]<br><br>[22]
**herbal cannabis from coffeeshops**<br><br>**herbal cannabis from coffee shops/**<br>**flower tops of pharmaceutical-grade varieties/**<br>**hemp dried flowers / 1.0 g**	-	Agilent 6890 + FID<br>DB-5MS (30 m x 0.25 mm x 0.25 μm)	COTP: 60°C, 3°Cmin^{1} to 240°C (5 min)<br>IV: 4 μL<br>injector: 230°C<br>split mode (1:20)<br>FID: 250°C	28 [23]/44 [24] major cannabinoids and terpenes, including: THCV, CBD, CBC, CBDV, CBGM, THC, CBG, CBN	65.00	THCV 6 000 000<br>CBD 6 000 000<br>CBC 6 000 000<br>CBDV 6 000 000<br>CBGM 6 000 000<br>Δ^9-THC 6 000 000<br>CBG 6 000 000<br>CBN 6 000 000<br>[25]	2011, the Netherlands<br>2010, the Netherlands<br>2016, the Netherlands	[23]<br>[25]<br>[24]
**homogenized inflorescences/ 0.02 – 0.03 g**	-	Agilent 6890 + FID<br>HP-1 (25 m x 0.32 mm x 0.5 μm)	COPT: 250°C, isothermal (9 min)<br>IV:1 μL<br>injector<br>split mode (1:25)<br>FID: 300°C	Δ^9-THC, CBD, CBN	9.00	-	2010, Belgium	[26]
**herbal cannabis / 0.2 g**<br>**herbal cannabis (stems, leaves and inflorescence) / 0.1 g**<br>**seized cannabis resin/ 0.05 g**<br>**herbal cannabis**	-	Agilent 6890N + FID<br>HP-5MS (30 m x 0.2 mm x 0.2 μm)	COTP: 60°C (2 min), 15°Cmin^{-1} to 280°C (5 min)<br>IV:1 μL<br>injector: 270°C<br>splitless mode<br>FID: 280°C	Δ^9-THC	22.00	-	2005-2014, Morocco	[27]

Matrix/amount	Derivatization conditions (derivatization agent amount, temperature, time)	instrument type /column	GC and detector conditions (COTP and other t)	compound identification	runtime (min)	LOD/*LOQ* (ngmL^{-1} or ng/g), * -LOD/*LOQ* expressed in % *(w/w)*	Year, country	Ref.
**herbal cannabis (apical segments) / 1.0 g**	-	Becker 3810 + FID<br>2% OV-17 (2 m x 4 mm)	COTP: 210°C, isothermal<br>IV: 2 μL<br>injector: 260°C<br>detector: 260°C	Δ^9-THC, CBDV, THCV, CBL, CBC, CBV, CBD, CME, Δ^8-THC, CBG, CBN	-	-	1979, South Africa	[28]
		Finnigan 3200/6100<br>2% OV-1 (20 m x 0.3 mm)	COTP: 100°C (2 min), 6°Cmin^{-1} to 230°C<br>injector: 230°C<br>split mode (1:10)<br>interface: 230°C<br>transfer line: 230°C					
**cannabis fruiting tops / cannabis resin / 0.5 g hash oil / 1.0 g**	-	HP 5730 + FID<br>2% OV-17 100-120 mesh Chromosorb WHP	COTP: 250°C, isothermal	Δ^9-THC, CBN, CBC, CBG, THCA	-	-	1979, Colombia	[29]
**herbal cannabis, powdered / cannabis resin, powdered / 0.01 g**	-	Agilent 6890 + FID<br>HP-5 (30 m x 0.32 mm x 0.25 μm)	COTP: 150°C (1 min), 15°Cmin^{-1} to 250°C (13 min)<br>IV: 1 μL<br>injector: 270°C<br>split mode (1:20)<br>FID: 280°C	Δ^9-THC, CBN, CBD	20.67	Δ^9-THC 0.03/*0.05*<br>CBN 0.03/*0.05*<br>CBD 0.03/*0.05*	2006-2007, Brazil	[30]*
**cannabis resin / 0.05 g**	-	Agilent 6890 + FID<br>HP-5 (15 m x 0.32 mm x 0.25 μm)	COTP: 180°C, 40°Cmin^{-1} to 220°C, 5°Cmin^{-1} to 240°C, 25°Cmin^{-1} to 280°C<br>injector: 280°C<br>split mode (1:30)<br>FID: 300°C	Δ^9-THC, CBD, CBN	8.00	Δ^9-THC 0.015/*0.030*<br>CBD 0.011/*0.041*<br>CBN 0.007/*0.026*	2002, Italy	[31]
***C. sativa* cultured by micropropagation (buds, leaves, roots and stems) / 0. 1 g**	dried extract + 100 μL BSTFA + 10 μL 2 % DMAP 70ºC, 30min	Agilent 6890N + FID<br>HP-5 (15 m x 0.25 mm x 0.25 μm)	COTP:190°C (1 min), 30°Cmin^{-1} to 230°C (2 min), 5°Cmin^{-1} to 250°C (1 min), 20°Cmin^{-1} to 300°C (2.75 min)	Δ^8-THC, Δ^9-THC, THCV, CBD, CBC, CBG. CBN, CBGA, THCA, CBDA	17.50	THCV 120/*380*<br>CBD 120/*35*<br>CBC 150/*460*<br>Δ^8-THC 140/*430*<br>Δ^9-THC 150/*450*<br>CBG 150/*47*	2017, USA	[32]

Matrix/amount	Derivatization conditions (derivatization agent amount, temperature, time)	instrument type /column	GC and detector conditions (COTP and other t)	compound identification	runtime (min)	LOD/*LOQ* (ngmL^{-1} or ng/g), * -LOD/*LOQ* expressed in % *(w/w)*	Year, country	Ref.
			injector: 275°C<br>split mode (1:20)<br>FID: 300°C			CBN 130/*410*<br>CBDA 140/*430*<br>THCA 190/*560*<br>CBGA 110/*340*<br>CBDV 100/*500*		
**dried cannabis flower buds/ 0.1 g**	-	Agilent 6890 GC + FID<br>ZB-5 (15 m x 0.25 mm x 0.25 µm)	COTP: 200°C (2 min), 10 ºCmin^{-1} to 240°C (2 min)<br>IV: 1.5 µL<br>injector: 280°C<br>split mode (1:20)<br>FID: 300°C	Δ^9-THC, CBD	8.00	-	2018, Czech Republic	[33]*
**dried cannabis flowers (with brachts), upper leaves, lower (large) leaves, stems**	-	Varian 204 or 2100 GC + FID<br>5% SE-30 100/120 mesh Gas Chrom P, 5% XE-60 80/100 mesh Chromosorb W 3% JXR100/120 mesh Gas Chrom Q<br>(all 1.8m x 2-3 mm)<br>LKB 9000 GC-MS	injector: 250-260°C<br>FID: 250-260°C	Δ^9-THC, CBD	-	-	1971, UNODC	[34]
**dried cannabis plant material, cannabis resin, reefer / 0.05 g**	-	Pye 104 FID<br>2% OV17 80-100 mesh Chromosorb W (1.524 m x 4 mm)	injector: 235-240°C	CBD, CBN, Δ^9-THC	-	-	1973, United Kingdom	[35]
**cannabis plant material/ 0.2 g cannabis resin / 0.1 g**	1.5 mL $CHCl_3$ + 100 µL MSTFA 70°C, 30 min	GC-FID<br>5% diphenyl 95% dimethylpolysilox	COTP: 200°C (2 min), 10°Cmin^{-1} to 240°C (2 min)<br>injector: 280°C<br>splitless mode/split mode (1:20)	CBD, Δ^9-THC, CBN	8.00	-	2009, UNODC	[36]

Matrix/amount	Derivatization conditions (derivatization agent amount, temperature, time)	instrument type /column	GC and detector conditions (COTP and other *t*)	compound identification	runtime (min)	LOD/*LOQ* (ngmL^{-1} or ng/g), * -LOD/*LOQ* expressed in % (*w/w*)	Year, country	Ref.
**cannabis oil / 0.05 g**		ane (15 m x 0.25 mm x 0.25 µm)	FID: 300°C					
**cannabis inflorescenses / 0.05 g** [37]<br><br>**cannabis leaves** [38]<br><br>**herbal cannabis / cannabis resin / sinsemilla / cannabis powder / 0.05 g**	-	HP 6890 + FID<br>**quantitative analysis:**<br>HP-5 (30 m x 0.32 mm x 0.25 µm)<br>**separation of CBC and CBD:**<br>HP-1 (40 m x 0.1 mm x 0.25 µm)	-	-	-	-	2003, Italy	[37]
						Δ^9-THC 1.06/*2.44*<br>CBN 1.06/*1.76*<br>CBD 1.02/*1.68*	2012, Colombia	[38]
						-	2005, England	[39]
**cannabis plant parts (bracts, floral leaves)**	-	HP 5710A GC + hydrogen FID<br>3% OV-1 80/100 mesh Supelcoport (2.43 m x 2 mm)	COTP: 180°C, 4°Cmin^{-1} to 240°C (8 min)<br>IV: 0.5 µL<br>inlet: 250°C<br>FID: 300°C	CBD, CBC, Δ^9-THC, CBN	24.00	-	1977, USA	[40]
**herbal cannabis / cannabis resin / hashish oil / 0.1 g** [41]<br><br>**herbal cannabis / sinsemilla / kilobrick / cannabis resin /**	-	Varian CP-3380 + dual FID<br>DB-5 MS (15 m x 0.25 mm x 0.25 µm)	COTP: 170°C (1 min), 10°Cmin^{-1} to 250°C (3 min)<br>IV: 1 µL<br>injector 240°C<br>split mode (100 mL/min, 1:50)<br>FID: 260°C	Δ^8-THC, Δ^9-THC, CBD, CBN, CBC, CBG, THCV	12.00	-	1993-2008, USA<br>1995-2014, USA<br>2008 - 2018, USA	[41]<br>[42]<br>[43]

Matrix/amount	Derivatization conditions (derivatization agent amount, temperature, time)	instrument type /column	GC and detector conditions (COTP and other *t*)	compound identification	runtime (min)	LOD/*LOQ* (ngmL^{-1} or ng/g), * -LOD/*LOQ* expressed in % *(w/w)*	Year, country	Ref.
**Thai sticks / hashish oil / ditchweed / 0.1 g [42]**<br><br>**herbal cannabis (marijuana, sinsemilla, ditchweed) / cannabis resin / hashish oil / 0.01 g [43]**<br><br>**herbal cannabis (indoor, outdoor) / 0.1 g [44]**		Varian CP-3380 + dual FID<br><br>DB-1 (15 m x 0.25 mm x 0.25 µm)					2009, USA	[44]
**dried cannabis plant material/ 0.05 g**	**Step1:** dry extract + 0.1 mL pyridine + 0.1 mL benzene + 1.0 mg alkylboronic acid room ºC, 30min<br><br>**Step2:** 0.1mL pyridine + 0.1 mL benzene + 0.5 mg methylboronic acid room ºC, 30 min<br><br>**Step 3:** 0.1 mL ACN + 0.1 mL BSTFA + 0.05 mL TMCS room ºC, 30 min	Varian 2400 + dual FID<br><br>glass column 3% SE-30 100-200 mesh Gas Chrom Q	COTP: 100°C, 4°Cmin^{-1} to 330°C<br><br>IV: 1 µL	-	57.50	-	1977, United Kingdom	[45]

Matrix/amount	Derivatization conditions (derivatization agent amount, temperature, time)	instrument type /column	GC and detector conditions (COTP and other t)	compound identification	runtime (min)	LOD/*LOQ* (ngmL^{-1} or ng/g), * -LOD/*LOQ* expressed in % *(w/w)*	Year, country	Ref.
	dried extract + 0.1 mL ACN + 0.1 mL BSTFA + 0.05 mL TMCS<br>room ºC, 30 min	Varian 2400 + VG Micromass 12B	COTP: 170-280°C, 2°Cmin^{-1}<br>injector: 280°C<br>inlet: 230°C<br>separator: 230°C<br>ion source: 260°C	EI, 25 eV<br>full scan mode<br>(*m/z* 40-680)<br>Δ^9-THCA, CBNA, CBDA	55.00			
**herbal cannabis / cannabis resin / 0.0025 g**	-	Trace Ultra™ (GC x GC) + FID<br>100% polysiloxane (30 m x 0.25 mm x 0.25 μm) + Carbowax (0.5 m x 0.1 mm x 0.1 μm)	COPT: 40°C (1 min), 10°Cmin^{-1} to 200°C, 2°Cmin^{-1} to 260°C (10 min)<br>IV: 0.5 μL<br>injector: 250°C<br>splitless mode<br>FID: 280°C	Δ^9-THC, CBD, CBN	57.00	-	2008, Germany	[46]
**hemp fruits / "seeded" flowers / hash oil / 1.0 g**	TMSH	HP 5890 + FID or HP 5870 MSD<br>OV-1 column	-	EI, 70 eV<br>Δ^8-THC, Δ^9-THC, CBN, CBD	-	-	1997, Germany	[47]
**dried cannabis leaves / 0.01 g**	-	Varian CP-3800 + FID<br>OV-1 (30 m x 0.53 mm x 0.50 μm)	COTP: 240°C isothermal<br>injector:260°C<br>IV: 1 μL<br>injector<br>split mode (1:5)<br>ion source: 250°C<br>quadrupole: 150°C<br>interface: 280°C	Δ^9-THC, CBN, CBD	-	-	2009, Colombia	[48]
		Agilent HP 6890 + HP 5973 MSD<br>DB-1 (30 m x 0.25 mm x 1.00 μm)	COTP: 240°C, isothermal<br>IV: 1 μL<br>injector: 260°C<br>split mode (1:5)<br>ion source: 250°C<br>quadrupole: 150°C<br>interface: 280°C	EI, 70 eV<br>full scan mode<br>(*m/z* 40-550)<br>Δ^9-THC, CBN, CBD		Δ^9-THC 1.06/*2.44*<br>CBN 1.06/*1.76*<br>CBD 1.02/*1.68*		

Matrix/amount	Derivatization conditions (derivatization agent amount, temperature, time)	instrument type /column	GC and detector conditions (COTP and other t)	compound identification	runtime (min)	LOD/*LOQ* (ngmL^{-1} or ng/g), * -LOD/*LOQ* expressed in % *(w/w)*	Year, country	Ref.
**seized cannabis female inflorescences, grounded / cannabis oil in olive oil / 0.05 g**	50 µL BSTFA + 1% TMCS + 50 µL toluene<br>70ºC, 30 min	Trace 2000 + FID<br>DB-5MS IU (30 m x 0.25 mm x 0.25 µm)	COTP: 200°C, 10°Cmin^{-1} to 300°C (2 min)<br>injector: 280°C<br>split mode (39 mL/min, 1:30)<br>FID: 300°C	Δ^{9}-THC, CBD, CBN, THCA, CBDA	12.00	-	2017, Italy	[49]
		HP 5973 + MSD<br>Rxi-5MS (30 m x 0.25 mm x 0.25 µm)	COTP: 70°C, 40°Cmin^{-1} to 180°C, 10°Cmin^{-1} to 300°C (6.25 min)<br>injector: 280°C<br>splitless mode<br>transfer line: 300°C<br>ion source: 230°C	EI, 70 eV<br>SIM mode<br>(*m/z* 50-600)<br>CBD-2TMS (390, 337, 301)<br>Δ^{9}-THC-TMS (386, 371, 315)<br>CBN-TMS (382, 368, 367)<br>CBDA-3TMS (559, 491, 453)<br>Δ^{9}-THCA-2TMS (487, 502)	21.00			
**dried, homogenized and grinded herbal cannabis / 0.2 g**	-	Agilent 7890N + FID<br>HP-5MS (30 m x 0.32 mm x 0.25 µm)	COPT: 150°C (1 min, at 10°Cmin^{-1} to 280°C, (5 min)<br>injector: 250°C<br>split mode (1:20)<br>interface: 300°C	EI, 70 eV<br>full scan mode<br>(*m/z* 40-450)<br>Δ^{8}-THC, Δ^{9}-THC, CBD, CBN, CBC, CBG, THCV	19.00	-	2012, Romania	[50]
		Agilent 6890N + 5973N MSD<br>HP-5MS (30 m x 0.25 mm x 0.25 µm)	COPT: 150°C (1 min), 10°Cmin^{-1} to 280°C (5 min)<br>injector: 290°C<br>splitless mode<br>interface: 300°C	Δ^{8}-THC, Δ^{9}-THC, CBD, CBN, CBC, CBG, THCV	19.00			
**cannabis mature floral clusters / 0.05 g**	-	HP 5890 + FID<br>ZB-624 (30 m x 0.32 mm x 0.25 µm)	COTP: 40°C (5 min), 10°Cmin^{-1} to 250°C (40 min)<br>injector<br>split mode (1:10)	Δ^{9}-THC, CBD, CBC, CBG, CBGM, CBDV, CBCV, THCV	47.00	-	2009, Italy	[51]

Matrix/amount	Derivatization conditions (derivatization agent amount, temperature, time)	instrument type /column	GC and detector conditions (COTP and other t)	compound identification	runtime (min)	LOD/*LOQ* (ngmL^{-1} or ng/g), * -LOD/*LOQ* expressed in % *(w/w)*	Year, country	Ref.
		HP 6890 + VG Trio MSD<br>ZB-5 (30 m x 0.32 mm x 0.25 μm)	COTP: 70°C, 5°Cmin^{-1} to 305°C<br>injector<br>split mode (1:5)	EI, 70 eV<br>Δ^9-THC, CBD, CBC, CBG, CBGM, CBDV, CBCV, THCV	66.00			
**sieved powder from whole cannabis plant / herbal cannabis / cannabis resin / 0.2 g**	-	Agilent 7890 or Shimadzu GC2010 + FID (for discrimination of CBD and CBC: GCMS-QP2010 Plus)<br>HP-5MS (30 m x 0.25 mm x 0.25 μm)	COTP: 200°C (2 min), 10C°min^{-1} to 240°C (15 min)<br>IV: 1 μL<br>inlet: 250°C<br>split mode (1:20)<br>FID: 300°C<br>for CBC and CBD:<br>COTP: 200°C (2 min), 10°Cmin^{-1} to 240°C (15 min)<br>IV: 1 μL<br>injector<br>split mode (1:20)	Δ^9-THC, CBN<br>EI, 70 eV<br>full scan mode<br>(*m/z* 40-400)<br>CBC, CBD	21.00	-	2010, Japan	[52]
**cannabis buds, milled / 0.05 g**	-	Agilent 7890A + FID + 5975C MSD<br>D1: HP-5MS (30 m x 0.25 mm x 0.25 μm)<br>D2: DB-17MS (5 m x 0.25 mm x 0.25 μm) + fused silica restrictors	COTP: 60°C, 4°Cmin^{-1} to 102°C, 12°Cmin^{-1} to 165°C, 6°Cmin^{-1} to 300°C (5 min)<br>IV: 2 μL<br>injector<br>splitless mode<br>FID: 300°C<br>quadrupole: 150°C<br>ion source: 230°C	EI, 70 eV<br>full scan mode<br>(*m/z* 50-350)	43.25	-	2014, Spain	[53]
	-	Clarus 680 GC + FID + Clarus Q 8T<br>SLB-5MS (30 m x 0.25 m x 0.25 μm)	COTP: 50˚C to 350˚C at 3˚Cmin^{-1}<br>injector: 280˚C<br>splitless mode	EI, 70 eV<br>full scan mode<br>(*m/z* 40-550)<br>CBDV, CBT, CBL, CBD, CBC, Δ^8-THC, Δ^9-THC	100.00	-	2021, Italy	[54]

Matrix/amount	Derivatization conditions (derivatization agent amount, temperature, time)	instrument type /column	GC and detector conditions (COTP and other *t*)	compound identification	runtime (min)	LOD/*LOQ* (ngmL^{-1} or ng/g), * -LOD/*LOQ* expressed in % (*w/w*)	Year, country	Ref.
			ion source: 220˚C<br>interface: 250˚C<br>FID: 300˚C					
**dried herbal cannabis/ 2.0g or 5.0 g**	-	Agilent 7820 + FID<br>HP-5 (30 m x 0.32 mm x 0.25 μm)	COTP: 60°C, 3°Cmin^{-1} to 240°C (10 min)<br>inlet: 250°C<br>split mode (1:20)<br>IV: 5 μL	CBD, CBN, Δ^8-THC, Δ^9-THC, THCA, CBDA, CBD-d$_3$, CBN-d$_3$, Δ^9-THC-d$_3$	70.00	CBDA 7.5/*25.1*<br>CBD 6.1/*20.4*<br>CBN 9.2/*30.7*<br>Δ^9-THC 9.4/*31.3*<br>Δ^8-THC 6.8/*22.6*<br>THCA 12.4/*41.2*	2021, Czech Republic	[55]
	dry extract + 50 μL dry EtAc + 50 μL BSTFA + 1% TMCS 70°C, 30 min		COTP: 180°C (2 min), 20°Cmin^{-1} to 310°C (1.5 min)		10.00			
		Agilent 7890A + HP 5975C MSD<br>HP-5MS (30 m x 0.25 mm x 0.25 μm)	COTP: 180°C (2 min), 20°Cmin^{-1} to 310°C (1.5 min)<br>injector: 260°C<br>split mode (1:9)<br>IV: 5 μL<br>ion source: 230°C	EI, 70 eV<br>SIM mode<br>CBDA-3TMS (73, **491,** 559)<br>CBD-2TMS (73, 337, **390**)<br>CBN-2TMS (73, **367**, 382)<br>Δ^9-THC-TMS (73, **371**, 386)<br>Δ^8-THC-TMS (73, **303**, 386)<br>THCA-2TMS (73, **487**)<br>CBD-d$_3$-2TMS (73, 340, **393**)<br>CBN-d$_3$-TMS (73, **370**, 385)<br>Δ^9-THC-d$_3$-TMS (73, **374**, 389)	10.00	CBDA 3.6/*12.0*<br>CBD 4.6/*15.4*<br>CBN 6.1/*20.4*<br>Δ9-THC 2.6/*8.7*<br>Δ8-THC 4.1/*13.5*<br>THCA 5.9/*19.6*		
**herbal cannabis ("loose marijuana", buds, kilobricks and "domestic marijuana", all stemless and seedless) / cannabis resin / 0.1 g**	-	Varian CP3880 + FID<br>DB-5 (30 m x 0.25 mm x 0.25 μm)	COTP: 150°C (1 min), 10°Cmin^{-1} to 290°C (2 min)<br>injector: 200°C<br>split mode (50 mL/min, 1:30)<br>FID: 300°C	Δ^9-THC	17.00	-	1997-2004, Modena, Italy	[56]
		Varian 3400 + Saturn 2000 IT<br>DB-5MS (30 m x 0.25 mm x 0.25 μm)	COTP: 120°C (1 min), 10°Cmin^{-1} to 290°C (10 min)<br>injector: 250°C<br>split mode (50 mL/min, 1:30)	EI, 70 eV<br>full scan mode<br>(*m/z* 43-500)<br>Δ^9-THC	28.00			

Matrix/amount	Derivatization conditions (derivatization agent amount, temperature, time)	instrument type /column	GC and detector conditions (COTP and other t)	compound identification	runtime (min)	LOD/*LOQ* (ngmL^{-1} or ng/g), * -LOD/*LOQ* expressed in % *(w/w)*	Year, country	Ref.
			transfer line: 280°C					
**beer, liquor, cannabis oil, pastilles, seeds, scented grass / 1.0 g (solid samples) / 1.0 mL (liquid samples)**	dry extract + 100 µL MSTFA + 2% TMCS<br>70°C, 30 min	Agilent 6890 + 5973 N MSD<br>HP-5MS (30 m x 0.25 mm x 0.25 µm)	COTP: 120°C (2 min), 20°Cmin^{-1} to 290°C (10 min)<br>injector: 260°C<br>split mode (1:15)	EI, 70 eV<br>SIM mode<br>Δ^9-THC-2TMS (386, **371**, 303)<br>CBD-2TMS (458, 390, 337)<br>CBN-2TMS (382, 367, **310**)	21.00	Δ^9-THC 0.30/*1.00*<br>CBD 0.30/*1.00*<br>CBN 0.60/*2.00*	2003, Italy	[57]
**cannabis tea / 0.05g / chocolate/snack bar / 0.4 g flour, seeds, fruit bar, nibbles / 0.1 g pastilles / 1.0 g oil / 0.1 mL lemonade, beer / 0.5 mL**<br>**tea infusion, shampoo / 1.0 mL**	HS-SPME: 25 µL MSTFA<br>90°C, 8 min<br>(on-coating derivatization)<br>LLE: dry extract + 20 µL pyridine + 50 µL MSTFA + 130 µL isooctane<br>90ºC, 15 min	Agilent 6890N + 5973 MSD<br>HP-5MS (30 m x 0.25 mm x 0.25 µm)	COTP: 160°C (1 min), 15°Cmin^{-1} to 190°C (1 min), 5°Cmin^{-1} to 250°C (1 min), 20° min^{-1} to 300°C (2 min)<br>injector: 250°C<br>splitless mode<br>ion source: 230°C<br>quadrupole: 150°C<br>interface: 280°C	EI, 70 eV<br>SIM mode<br>Δ^9-THC-2TMS (303, **371**, 386)<br>CBD-2TMS (301, 337, **390)**<br>CBN-2TMS (**367**, 368, 392)	21.50	**tea leaves**<br>Δ^9-THC 0.01/0.08<br>CBD 0.12/0.32<br>CBN 0.01/0.09<br>**chocolates**<br>Δ^9-THC 0.03/0.06<br>CBD 0.17/0.35<br>CBN: 0.03/0.07<br>**oils**<br>Δ^9-THC 0.05/0.03<br>CBD 0.09/ 0.34<br>CBN 0.15/0.43	2002, Germany	[58]

Matrix/amount	Derivatization conditions (derivatization agent amount, temperature, time)	instrument type /column	GC and detector conditions (COTP and other t)	compound identification	runtime (min)	LOD/*LOQ* (ngmL^{-1} or ng/g), * -LOD/*LOQ* expressed in % *(w/w)*	Year, country	Ref.
**dried herbal cannabis (stems, leaves and inflorescence) / 0.1 g**	-	Agilent 6890N + 5973 MSD<br>HP-5MS (30m x 0.25mm x 0.25 μm)	COTP: 100°C (1 min), 10°Cmin^{-1} to 260°C<br>injector: 280°C<br>split mode (1:20)<br>transfer line: 250°C<br>ion source: 150°C<br>quadrupole: 150°C	EI, 70 eV<br>full scan mode<br>(*m/z* 50-500)<br>Δ^{9}-THC, CBD, CBN, CBC, CBG	17.00	-	2016, Brazil	[59]
**cannabis plants (recreational, medical, hemp) / consumer products (oral supplements, foods, candies, beverages, vapes, liquids, topicals) / medical products / illicit products (kief, hash oil)/ 0.03 – 3.0 g**	dry extract + 200 μL pyridine + 200 μL BSTFA 80ºC, 30 min	Agilent 6890N + 5973 MSD<br>Rxi-35Sil MS (30 m x 0.25 mm x 0.25 μm)	COTP: 60°C (0.5 min), 25°Cmin^{-1} to 220°C (10 min), 10°Cmin^{-1} to 300°C (15 min)<br>IV: 1 μL<br>injector: 250°C<br>splitless mode<br>transfer line: 280°C	EI, 70 eV<br>full scan mode<br>(*m/z* 40-600)<br>CBD, CBDA, Δ^{9}-THCA, Δ^{9}-THC	39.90	CBD 1000<br>CBDA 1000<br>Δ^{9}-THC 1000<br>THCA 1000<br>CBN 1000	2017, USA	[60]

Matrix/amount	Derivatization conditions (derivatization agent amount, temperature, time)	instrument type /column	GC and detector conditions (COTP and other t)	compound identification	runtime (min)	LOD/*LOQ* (ngmL^{-1} or ng/g), * -LOD/*LOQ* expressed in % *(w/w)*	Year, country	Ref.
**cannabis inflorescence (AK-47, amnesia, somango, critical), cryogenically miled / 0.05g for FUSE, 0.1g for SFE**	-	Agilent 6890 N + 5973 N MSD<br>HP-5MS (30 m x 0.25 mm x 0.25 µm)	COTP: 60°C, 8°Cmin^{-1} to 90°C, 70°Cmin^{-1} to 192°C, 3°Cmin^{-1} to 195°C, 70°Cmin^{-1} to 285°C, 10°Cmin^{-1} to 300°C (2.5 min)<br>IV: 2 µL<br>injector: 300°C<br>splitless mode<br>transfer line: 310°C<br>ion source: 230°C<br>quadrupole: 150°C	EI, 70 eV<br>full scan mode<br>(*m/z* 50-350)<br>Δ^9-THC, CBD, CBN	11.50	-	2013, Spain	[61]
**dry cannabis female flowering head material / 2.0 g**	-	Agilent 6890 + MSD<br>HP-5MS (30 m x 0.25 mm x 0.25 µm)	COTP: 80°C (1 min), 50°Cmin^{-1} to 300°C (9.6 min)<br>inlet: 280°C<br>detector: 325°C	EI, 70 eV<br>full scan mode<br>total THC (Δ^9-THC + Δ^9-THCA)	15.00	-	2010, New Zealand	[62]
**no real samples**	-	Agilent 5975 + 5973 MSD<br>JW Ultra 1 (12 m x 0.2 mm x 0.33 µm)<br>**DB-35MS (30 m x 0.25 mm x 0.25 µm)**<br>DB-1701 (30 m x 0.25 mm x 0.25 µm)	COTP: 90°C (0.5 min), 5°Cmin^{-1} to 300°C (15 min)<br>IV: 1 µL<br>injector<br>split mode (50 mLmin^{-1})<br>detector: 280°C	EI, 70 eV<br>full scan mode<br>(*m/z* 30-550)<br>CBC, CBD, CBG, CBN, CBDV, THCV, Δ^8-THC, Δ^9-THC	57.50	CBDV 1000/2850<br>THCV 710/710<br>CBD 2850/2850<br>CBC 2850/2850<br>Δ^8-THC 710/710<br>Δ^9-THC 710/710<br>CBG 10000/12500<br>CBN 1420/2850	2017, USA	[63]
**cannabis leaves / 0.1 g**	-	Agilent 7890A + 5975C MSD	COTP: 100°C, 10°Cmin^{-1} to 260°C (10 min)<br>IV: 2 µL<br>injector: 280°C	EI, 70 eV<br>SIM mode<br>(*m/z* 30-450)<br>THCV (**271**, 286, 243, 203)	26.00	-	2010, Switzerland	[64]

Matrix/amount	Derivatization conditions (derivatization agent amount, temperature, time)	instrument type /column	GC and detector conditions (COTP and other t)	compound identification	runtime (min)	LOD/*LOQ* (ngmL^{-1} or ng/g), * -LOD/*LOQ* expressed in % *(w/w)*	Year, country	Ref.
		HP-5MS (30 m x 0.25 mm x 0.25 µm)	split mode (1:10)<br>transfer line: 250°C<br>ion source: 230°C<br>quadrupole: 150°C	CBL (**231**, 232, 314, 174)<br>CBD (**231**, 174, 314, 299)<br>Δ^9-THC (**299**, 314, 231, 271)<br>CBG (**193**, 231, 123, 316)<br>CBN (**295**, 238, 310, 223)				
**fresh cannabis flowers/ 0.6 g**	-	Agilent 7890B GC + 5977A MSD<br>HP-5MS (30 m x 0.25 mm x 0.25 µm)	COTP: 50°C (2 min), 6°Cmin^{-1} to 300°C, 300°C (4 min)<br>IV: 1 µL<br>split mode (1:10)<br>interface: 280°C	EI: 70 eV<br>(*m/z* 40–500)<br>CBG, CBD, CBN, Δ^9-THC, CBC	51.00	-	2018, Israel	[65]
**cold pressed hemp seed oil/ 3.0 g**<br>**hemp seeds/ 3.0 g**<br>**hemp proteins/ 3.0 g**<br>**hemp teas/ 0.25 g**<br>**confectioneries/alcoholic beverages / 5.0 g**	-	Agilent 7890B GC + 5977A MSD<br>-	COTP: 50°C (2 min), 40°Cmin^{-1} to 270°C (7 min), 30°Cmin^{-1} to 280°C (3 min)<br>injector: 260°C<br>split mode (1:10)<br>transfer line: 280°C<br>ion source: 230°C<br>quadrupole: 150°C	EI: 70 eV<br>full scan mode<br>(*m/z* 150-330)<br>SIM mode<br>CBD (**231**, 246, 209)<br>CBNN (**299**, 231, 314)<br>Δ^9-THC (**295**, 238, 310)	17.83	CBD<br>oil 0.0005/*0.001*<br>tea 0.01/*0.02*<br>protein 0.001/*0.002*<br>seed 0.001/*0.002*<br>chocolate0.001/*0.002*<br>CBN<br>oil 0.0001/*0.0005*<br>tea 0.002/*0.01*<br>protein 0.0005/*0.001*<br>seed 0.0005/*0.001*<br>chocolate0.0005/*0.001*<br>THC<br>oil 0.0005/*0.001*<br>tea 0.003/*0.010*<br>protein0.0002/*0.0005*<br>seed 0.0005/*0.001*<br>chocolate<br>0.0002/*0.0005*	2018-2019, Europe	[66]
**cannabis resin / 0.05 g**	-	Agilent 6890 + 5975C MSD	COTP: 100°C, 10°Cmin^{-1} to 260°C (10 min)<br>IV: 2 µL	EI, 70 eV<br>SIM mode<br>THCV (**271**, 286, 243, 203)	26.00	-	2013, Switzerland	[67]

Matrix/amount	Derivatization conditions (derivatization agent amount, temperature, time)	instrument type /column	GC and detector conditions (COTP and other *t*)	compound identification	runtime (min)	LOD/*LOQ* (ngmL^{-1} or ng/g), * -LOD/*LOQ* expressed in % *(w/w)*	Year, country	Ref.
		HP-5MS (30 m x 0.25 mm x 0.25 µm)	injector: 280°C<br>split mode (1:10)<br>transfer line: 250°C<br>ion source: 230°C<br>quadrupole: 150°C	CBL (**231**, 232, 314, 174)<br>CBD (**231**, 174, 314, 299)<br>Δ^9-THC (**299**, 314, 231, 271)<br>CBG (**193**, 231, 123, 316)<br>CBN (**295,** 238, 310, 223)				
**Hempseeds / 1.0 g hempseed oil/ 0<br>2 mL**	-	Agilent 6890 N GC + 5975 MSD<br>HP-5MS (30 m x 0.25 mm x 2.5 µm)	COTP: 80°C (1 min), 20°Cmin^{-1} to 240°C, 5°Cmin^{-1} to 260°C, 20°Cmin^{-1} to 300°C (10 min)<br>IV: 1 µL	EI, 70 eV<br>full scan mode<br>THC **299**, 314<br>CBD **231**, 246<br>CBN **295**, 296, 310<br>THC-d_3 **302**, 317<br>CBD-d_3 **234**, 249<br>CBN-d_3 **298**, 299, 313	25.00	<u>THC</u><br>sunflower seeds 10.0/*50.0*<br>olive oil 20.0/*50.0*<br><u>CBD</u><br>sunflower seeds 5.0/*10.0*<br>olive oil 5.0/*10.0*<br><u>CBN</u><br>sunflower seeds 5.0/*10.0*<br>olive oil 5.0/*10.0*	2020, South Korea	[68]
**seized herbal cannabis (flower and leaf) / cannabis resin / 0.01 g**	-	Agilent 7890 + MSD<br>DB-5 (30 m x 0.25 mm x 025 µm)	COTP: 80°C (2 min), 10°C min^{-1} to 290°C (5 min)<br>injector: 280°C<br>split mode (10:1)	EI, 70 eV<br>full scan mode<br>(*m/z* 50-400)<br>Δ^9-THC, CBD, CBC, CBG, CBN	28.00	-	2019, Brazil	[69]
		GCMS QP 2010 Shimadzu Ultra (GC x GC)<br>D1: DB-5 (30 m x 0.25 mm x 0.25 µm)<br>D2: D-17 (1.8 m x 0.1 mm x 0.1 µm)	COTP: 80°C (5 min), at 7°Cmin^{-1} to 300°C (10 min)<br>IV: 1 µL<br>injector: 280°C<br>splitless mode<br>interface: 300°C<br>ion source: 300°C	EI, 70 eV<br>full scan mode<br>(*m/z* 50-550)<br>Δ^9-THC, CBD, CBC, CBG. CBN, THCA, CBDA	46.45			
**cannabis seeds / roots/ leaves / stems / flowers / 0.01 g**	-	Shimadzu GC-2010 + Shimadzu QP 2010 S	COTP: 100°C (1 min), 20°Cmin^{-1} to 290°C (10 min)	EI, 70 eV<br>SIM mode<br>(*m/z* 40-600)	20.50	Δ^9-THC 0.005/*0.01*<br>CBD 0.005/*0.01*<br>CBN 0.005/*0.01*	2008-2011, Albania	[70]*

Matrix/amount	Derivatization conditions (derivatization agent amount, temperature, time)	instrument type /column	GC and detector conditions (COTP and other t)	compound identification	runtime (min)	LOD/*LOQ* (ngmL^{-1} or ng/g), * -LOD/*LOQ* expressed in % *(w/w)*	Year, country	Ref.
**crushed and riddled plant**		HP-5MS (30 m x 0.25 mm x 0.25 µm)	IV: 1 µL<br>injector<br>split mode (1:10)	Δ^9-THC (**299**, 314, 231)<br>CBD (**231**, 174, 314)<br>CBN (**295**, 238, 310)				
***C.indica* resin, grounded/ 10.0 g**	-	Shimadzu GC-QP 2010 Plus<br>DB-5MS (3 0m x 0.25 mm x 0.25 µm)	COTP: 100°C (2 min), 10°Cmin^{-1} to 300°C (10 min)<br>IV: 1 µL<br>injector: 300°C<br>split mode<br>ion source: 280°C	EI, 70 eV<br>full scan mode<br>(*m/z* 85-380)<br>Δ^9-THC, CBD, CBN, CBC, CBD, CBG, THCV, CBP, CBV, HHCBN	42.00	-	2012, Pakistan	[71]
**dried *C. indica* leaves and stems, powdered / 10.0 g**	-	Shimadzu GC- QP 2010 Plus<br>DB-5MS (30 m x 0.25 mm x 0.25 µm)	IV: 1 µL<br>injector: 300°C<br>split mode<br>ion source: 280°C<br>interface: 280°C	EI, 70 eV<br>full scan mode<br>THCV, CBV, CBD, CBC, CBG, Δ^9-THC, CBN	-	-	2015, Pakistan	[72]
**dried hemp inflorescence (*C.sativa L.* futura, 75 variety), powdered / 0.025 g**	50 µL pyridine + 150 µL MSTFA + 1% TMCS	Shimadzu QP 2010 Plus<br>Restek RTX-5 (10 m x 0.1 mm x 0.1 µm)	COTP: 180°C (0.5 min), 10°Cmin^{-1} to 250°C, 60°Cmin^{-1} to 350°C (5 min)<br>IV: 1 µL<br>injector: 300°C<br>split mode (1:30)<br>interface: 330°C<br>ion source: 200°C	EI, 70 eV<br>SIM mode<br>THCV-TMS (**343**, 358, 315, 278)<br>CBD-2TMS (**390**, 458, 301, 337)<br>CBC-TMS (**303**, 371, 386, 246)<br>Δ^8-THC-TMS (**386**, 303, 265, 330)<br>Δ^9-THC-TMS (**371**, 386, 315, 303)<br>CBG-2TMS (**337**, 321, 460, 391)<br>CBN-TMS (**367**, 310, 382, 295)<br>CBDA-2TMS (**491**, 453, 559, 492)<br>THCA-2TMS (**487**, 488, 550, 413)<br>CBGA-3TMS (**561**, 5662, 417, 453)	8.33	THCV 3.97/*12.00*<br>CBD 4.29/*6.63*<br>CBC 4.62/*8.91*<br>Δ^8-THC 2.91/*8.82*<br>Δ^9-THC 9.08/*18.40*<br>CBG 4.07/*12.30*<br>CBN 4.12/*12.50*<br>CBDA 7.66/*23.20*<br>THCA 7.75/23.50<br>CBGA 9.40/25.50	2018, Italy	[73]

Matrix/amount	Derivatization conditions (derivatization agent amount, temperature, time)	instrument type /column	GC and detector conditions (COTP and other t)	compound identification	runtime (min)	LOD/*LOQ* (ngmL^{-1} or ng/g), * -LOD/*LOQ* expressed in % *(w/w)*	Year, country	Ref.
**fresh cannabis seeds, whole / 0.5 g**	-	HP 5890A + 5970A MSD<br>DB-1 (15 m x 0.25 mm x 0.25 μm)	COTP: 170°C (1 min), 10°Cmin^{-1} to 250°C (10 min)<br>splitless mode	EI, 70 eV<br>SIM mode<br>Δ^9-THC (314, 299, 231)	19.00	-	2000, USA	[74]
**cannabis resin / 0.1 g**	-	HP 5890 + 5971 MSD<br>SE-52 (30 m x 0.25 mm x 0.25 μm)	COPT: 80°C (3 min), 20°Cmin^{-1} to 260°C<br>IV: 1 μL<br>injector: 280°C<br>split mode (1:20)<br>detector: 285°C<br>ion source: 180°C	EI, 70 eV<br>full scan mode<br>(*m/z* 50-500)<br>Δ^9-THC, CBD, CBN	12.00	-	2006, Italy	[75]
**dried herbal cannabis, grounded / 0.06 g**	-	HP 5890 + HP 5972 MSD<br>HP-5MS (30 m x 0.25 mm x 0.25 μm)	COTP: 150°C (2 min), 30°Cmin^{-1} to 210°C, 5°Cmin^{-1} to 250°C, 10°Cmin^{-1} to 280°C<br>injector: 280°C<br>splitless mode<br>transfer line: 280°C	EI, 70 eV<br>full scan mode<br>(*m/z* 30-400)<br>Δ^9-THC, CBD, CBN, THCV, CBG, CBV, CBC, CBCL	15.00	-	2003, Switzerland	[76]
**dried herbal cannabis, grinded / 0.1 g**	-	HP 5980 II$^+$ + 5989B MSD<br>HP-5MS (30 m x 0.25 mm x 0.25 μm)	COTP: 200-280°C<br>IV: 2 μL<br>injector: 280°C<br>split mode (1:50)<br>transfer line: 260°C<br>ion source: 250°C<br>quadrupole: 120°C	EI, 70 eV<br>SIM mode<br>THC (299, 314)<br>CBD (231, 314)	-	-	1999, Austria	[77]
**cannabis resin bars / 0.025 g**	-	HP 5890 II + HP 5972 MSD<br>BP-5 (30 m x 0.25 mm x 0.25 μm)	COTP: 100°C (2 min), 10°Cmin^{-1} to 300°C (15 min)<br>IV: 1 μL<br>ion source: 280°C	EI, 70 eV<br>full scan mode<br>(*m/z* 100-600)<br>Δ^9-THC, CBD, CBN	37.00	-	2005, United Kingdom	[78]

Matrix/amount	Derivatization conditions (derivatization agent amount, temperature, time)	instrument type /column	GC and detector conditions (COTP and other t)	compound identification	runtime (min)	LOD/*LOQ* (ngmL^{-1} or ng/g), * -LOD/*LOQ* expressed in % *(w/w)*	Year, country	Ref.
**herbal cannabis, powdered / 0.06 g**	-	HP 5890 + HP 5972 MSD<br>HP-5MS (30 m x 0.25 mm x 0.25 μm)	COTP: 150°C (2 min), 30°Cmin^{-1} to 210°C, 5°Cmin^{-1} to 250°C, 10°Cmin^{-1} to 280°C (3 min)<br>injector: 280°C<br>splitless mode<br>transfer line: 280°C	EI, 70 eV<br>full scan mode<br>(*m/z* 30-400)<br>Δ^9-THC, CBD, CBN	18.00	-	2005, Switzerland	[79]
**commercial-grade hempseed oil / 1.0 mL**	-	Trace GC + DSQ II MSD<br>ZB-5MS (30 m x 0.25 mm, 0.25 μm)	COTP: 60°C (2 min), 15°Cmin^{-1} to 260°C (5 min)<br>IV: 1 μL<br>injector: 260°C<br>splitless mode<br>transfer line: 270°C<br>ion source: 200°C	EI, 70 eV<br>SIM mode<br>Δ^9-THC (231, 299, 314)<br>CBD (231, 246, 314)<br>CBN (295, 296, 310)	21.00	-	2014, Croatia	[80]
**cannabis inflorescences, cannabis-based drugs, cannabis extracts / 0.5-1.0 mg**	-	Agilent 7890 GC + 5977 MSD (+ Aviv Analytical SMB interface and its dual cage flight-through ion source)<br>DB-1HT (15 m x 0.32 mm x 0.1 μm)	COTP: 50°C (0 min), 20°Cmin^{-1} to 330°C (3 min)<br>split mode (1:10)	cold EI, 70 eV<br>full scan mode	17.00	-	2021, Israel	[81]
**cannabis oil / 1.0 g**	-	Perkin Elmer GC AutoSystem XL + MS Turbo Mass<br>MDN-5S (30 m x 0.25 mm x 0.25 μm)	COTP: 50°C (2 min), 10°Cmin^{-1} to 300°C, 300°C (3 min)<br>IV: 1 μL<br>injector: 250°C<br>splitless mode<br>ion source: 280°C<br>interface: 280°C	EI, 70 eV<br>SIM mode<br>(*m/z* 40-400)<br>Δ^9-THC (231), CBD (231), CBC (231), CBN (295)	30.00	Δ^9-THC 5 000 000<br>CBD 5 000 000<br>CBC 5 000 000<br>CBN 4 000 000	2005, Japan	[82]

Matrix/amount	Derivatization conditions (derivatization agent amount, temperature, time)	instrument type /column	GC and detector conditions (COTP and other *t*)	compound identification	runtime (min)	LOD/*LOQ* (ngmL^{-1} or ng/g), * -LOD/*LOQ* expressed in % *(w/w)*	Year, country	Ref.
**cannabis inflorescences (fiber-type), powdered / 0.4 g**	-	Varian 3400 GC + Finnigan SSQ 710 single-stage quadrupole MSD	COTP: 120°C (1 min), 30°Cmin^{-1} to 295°C, 295°C (13 min)<br>injector: 295ºC<br>splitless mode	THC (299, 314)	20.00	THC 0.003	2005, Australia	[83]*
**no real samples**	-	Varian 380 GC + Saturn 2000 IT<br>DB-1MS (30 m x 0.25 mm x 0.1 μm)<br>HP-50+ (30 m x 0.25 mm x 0.15 μm)	COTP: 100°C, 10°C min^{-1} to 280°C (12 min)<br>injector: 280°C<br>split mode (1:50)<br>detector: 290°C	EI, 70 eV<br>full scan mode<br>Δ^9-THC, Δ^8-THC, THCV, CBL, CBD, CBC, CBG, CBN	30.00	-	2005, Switzerland	[84]
**fresh herbal cannabis (inflorescences, lower-/upper-part leaves (1:1))/ dried herbal cannabis / 0.1 g / powdered cannabis / 0.01 g**	-	Varian CP-3800 + Saturn 2000 IT<br>HP-5 (25 m x 0.2 mm x 0.11 μm)	COTP: 60°C (2 min), 15°Cmin^{-1} to 280°C (5 min)<br>IV: 1 μL<br>injector: 270°C splitless mode	Δ^9-THC, CBD, CBN, Δ^9-THCA, CBDA, CBNA	22.00	-	2004, Morocco	[85]
**dried *C. ruderalis* female inflorescences, pulverized / 0.0005-0.002 g**	125 μL pyridine + 225 μL HMDS + 25 μL TFA<br>100ºC, 90 min	Varian 240 GC-IT<br>HP-5MS (30 m x 0.25 mm x 0.25 μm)	COTP: 100°C, 20°Cmin^{-1} to 300°C (10 min for TBDMS derivatives)<br>IV: 1 μL<br>injector: 300°C (4 min)<br>transfer line: 300°C<br>IT: 210°C<br>manifold: 80°C	EI, 70 eV<br>full scan mode<br>Δ^9-THC, CBC, CBD, CBG, CBN	13.00<br>20.00 (TBDMS derivati ves)	20-80 ng/mL injected sample<br>THC *-/0.03*<br>CBN *-/0.02*	2018, Hungary	[86]

Matrix/amount	Derivatization conditions (derivatization agent amount, temperature, time)	instrument type /column	GC and detector conditions (COTP and other t)	compound identification	runtime (min)	LOD/*LOQ* (ngmL^{-1} or ng/g), * -LOD/*LOQ* expressed in % *(w/w)*	Year, country	Ref.
**no real samples**	dried standard solutions + 50 µL EtAc + 50 µL BSTFA + 1% TMCS 70˚C, 30 min (for Δ^9-THCA)	Shimadzu GC-MS 8030 + QQQ<br>Rxi-5MS (20 m x 0.18 mm x 0.18 µm)	COTP: 40°C (1 min), 20°Cmin^{-1} to 200°C, 3°Cmin^{-1} to 300°C (3 min)<br>IV: 1 µL injector splitless mode (THCV, CBD, CBC, Δ^8-THC, CBG, CBN) split mode (1:80 Δ^9-THC, Δ^9-THCA, in some cases CBD) ion source: 230°C interface: 250°C	EI, 70 eV SIM mode<br>THCV (41, 43, 271, 203) CBD (231, 232, 174, 246) CBC (231, 232, 174, 41) Δ^8-THC (231, 314, 258, 271) CBG (193, 123, 231, 41) CBN (295, 296, 238, 310) Δ^9-THC (299, 314, 231, 271) Δ^9-THCA-2TMS (487, 73, 365, 147)	46.00	THCV 20.00<br>CBD 10.00<br>CBC 10.00<br>Δ^8-THC 20.00<br>CBG 9.00<br>CBN 10.00<br>CBD 1300.00<br>Δ^9-THC 1300.00<br>Δ^9-THC-TMS (BSTFA + 1% TMCS) 850.00<br>Δ^9-THC-TMS (MSTFA) 1210.00<br>Δ^9-THCA-2TMS (BSTFA + 1% TMCS) 3800.00<br>Δ^9-THCA-2TMS (MSTFA) 6000.00	2016, USA	[87]
**dried cannabis leaves and inflorescences (17 outdoor + 5 indoor cultivars) / 0.1 g**	dried extracts + 270 µL BSTFA + 2% TMCS + 30 µL pyridine 37°C, 60 min	Agilent 7890A GC + Agilent 7200 UHD Accurate-Mass QTOF<br>DB-5MS-UI (30 m × 0.25 mm × 0.25 µm)	COTP: 50°C, 2°Cmin^{-1} to 104°C (27 min), 20°Cmin^{-1} to 120°C (0.8 min), 4°Cmin^{-1} to 160°C (10 min), 25°Cmin^{-1} to 232°C (2.9 min), 1.5°Cmin^{-1} to 242°C (6.7 min), 2°Cmin^{-1} to 250°C (4 min), 25°Cmin-^{1} to 300°C (12 min)<br>IV: 1 µL injector: 250°C splitless mode (derivatized samples, split mode (1:10)) transfer line: 305°C ion source: 305°C quadrupole: 200°C	EI, 70 eV (*m/z* 50-750)<br>- (untargeted analysis)	89.40	-	2019, Spain	[88]

Matrix/amount	Derivatization conditions (derivatization agent amount, temperature, time)	instrument type /column	GC and detector conditions (COTP and other t)	compound identification	runtime (min)	LOD/*LOQ* (ngmL^{-1} or ng/g), * -LOD/*LOQ* expressed in % *(w/w)*	Year, country	Ref.
**no real samples**	dried solution + 500 µL EtAc + 500 µL BSTFA + 1% TMCS 70ºC, 30 min	GC 2010 + VGA 100 (VUV)<br>RTX-5 (30 m x 0.25 mm x 0.25 µm)	COTP: 40°C (1 min), 30°Cmin^{-1} to 220°C, 10°Cmin^{-1} to 260°C (5 min)<br>IV: 1 µL<br>injector: 250°C<br>splitless mode<br>transfer line: 250°C	THCV, CBD, CBC, Δ^8-THC, Δ^9-THC, CBG, CBN, Δ^9-THCA	16.00	THCV 3*/5**/-<br>CBD 3*/5**/ -<br>CBC 3*5**/-<br>Δ^8-THC 3* / 5**/-<br>Δ^9-THC 5*/10**/-<br>CBG 3*/5**/-<br>CBN 3*/5**/-<br>Δ^9-THCA 3*/5**/-<br>11-nor-9-carboxy- Δ^9-THC 3*/5**/-<br>11-OH-Δ^9-THC 3*/5**/-<br>*derivatized<br>**underivatized	2018, USA	[89]
**fresh cannabis female flower tops / 0.3 g (PLE), 2.0 g (LLE)**	dried extract + 100 µL BSTFA + 1% TMCS<br>room °C, 15 min	Shimadzu GC-17A + FID<br>ZB-5 (30 m x 0.32 mm x 0.50 µm)	COTP: 170°C, 15°Cmin^{-1} to 260°C (10 min), 5°Cmin^{-1} to 280°C<br>IV: 1 µL	Δ^9-THC, CBN, Δ^9-THCA	18.00	-	2015, Poland	[90]
**cannabis herbal material, crude / 25.0 g**	-	GC-MSD<br>DB-5 (15 m x 0.25 mm x 0.25 µm)	COTP: 200°C (2 min), 10°Cmin^{-1} to 240°C (2 min)<br>IV: 1.5 µL<br>injector: 280°C<br>split mode (1:20)<br>detector: 300°C	Δ^9-THC, CBD, CBN	8.00	-	2014, Pakistan	[91]
**dried female flower tops / 0.05 g, 0.1 g**	-	Chromapack CP9000 GC-FID<br>DB-1 (30 m x 0.25 mm x 0.1 µm)	COTP: 100°C, 10°Cmin^{-1} to 280°C<br>injector: 280°C<br>split mode (1:50)<br>FID: 290°C	Δ^9-THC, CBD, CBG, CBN, Δ^9-THCA, CBDA	30.00	-	2004, the Netherlands	[92]
		Varian 3800 GC + Saturn 2000 IT		EI, 70 eV<br>Δ^9-THC, CBD, CBG, CBN, Δ^9-THCA, CBDA,				

Matrix/amount	Derivatization conditions (derivatization agent amount, temperature, time)	instrument type /column	GC and detector conditions (COTP and other t)	compound identification	runtime (min)	LOD/*LOQ* (ngmL^{-1} or ng/g), * -LOD/*LOQ* expressed in % *(w/w)*	Year, country	Ref.
		VA-5MS (30 m x 0.25 mm x 0.25 μm)						

Table S2. LC-based analytical methods for cannabinoid profiling. APCI - atmospheric pressure-chemical ionization, CT - column temperature, CV - capillary voltage, ESI - electrospray ionization, FR - flow rate, IV - injection volume, MP - mobile phase, MPGP – mobile phases gradient program, MRM - Multiple Reaction Monitoring, SC-CO_2 – supercritical carbon dioxide, WL – wavelength.

Sample type/matrix/ amount (in grams or concentration)	Instrument type and column	LC conditions: mobile phases, MPGP (A%/B%/C%), FR, IV, CT	compounds MS data (quantification ion/MRM), WL	Runtime (min)	LOD/LOQ (ng/mL or ng/g), * - LOD/*LOQ* expressed in % (w/w)	Year, country	Reference
**dried cannabis inflorescence / 0.2 g**	Agilent 1100 HPLC + Waters 2996 DAD<br>XTerra® MS C_{18} (250 mm x 2.1 mm x 5 µm) + XTerra® MS C_{18} (10 mm x 2.1mm x 5µm)	MP A: H_2O + 50 mM NH_4COOH<br>MP B: MeOH + 50 mM NH_4COOH<br>0 min (32/68), 25 min (9.5/90.5), 26 min (5/95), 29 min (5/95),<br>30 min (32/68), 36 min (32/68)<br>FR: 0.3 mLmin^{-1}<br>IV: 30 µL<br>CT: 30°C	200-400 nm	36.00	Δ^9-THCA0.05/*0.025*<br>Δ^9-THC 0.05/*0.025*<br>CBDA 0.05/*0.05*<br>CBD 0.075/*0.075*<br>CBGA 0.05/*0.05*<br>CBG 0.15/*0.10*<br>CBN 0.05/*0.025*	2009, Belgium	[93]
	Shimadzu HPLC + DAD<br>Waters X-Bridge RP-C_{18} (150 mm x 4.6 mm, 3.5 µm) + Opti-Guard C_{18} (2.1 mm x 1 mm)	MP A: 50 mM NH_4COOH + 10% ACN<br>MP B: 90% ACN<br>0 min (30/70), 15 min (10/90), 30 min (10/ 90), 31 min (30/70), 40 min (30/70)<br>FR: 1 mLmin^{-1}<br>IV: 5 uL<br>CT: 25°C	272 nm	40.00	Δ^9-THC, CBD, CBG, CBC), CBN, THCV, Δ^9-THCA, CBDA, CBGA	2013, Australia	[94]
**dried and homogenized herbal cannabis (only flowers and leaves) / 0.5 g**	HPLC + DAD<br>LiChrospher® 60 RP-select B (250 mm x 4.0 mm, 5µm) + LiChrospher® 60 RP-select B (4.0 mm x 4.0 mm x 5.0 µm)	MP A: HPLC-grade H_2O<br>MP B: ACN<br>isocratic, 20:80, *v/v*<br>FR: 1 mLmin^{-1}<br>IV: 10 µL<br>CT: 30°C	220 nm, 240 nm	8.00	CBD, CBN, Δ^9-THC, Δ^9-THCA	2009, UNODC	[36]
**fresh cannabis inflorescences / 0.5 g**	HPLC + DAD<br>Poroshell 120 EC-C_{18} (150 mm x 3.0 mm x 2.7 µm) or equivalent + C_{18} (5 mm x 3 mm x 2.7 µm)	MP A: 85% o-H_3PO_4 in H_2O<br>MP B: ACN<br>0 min (36/64), 16 min (18/82), 17 min (36/64), 20 min (36/64)<br>FR: 1 mLmin^{-1}<br>IV: 10 µL<br>CT: 40°C	225 nm, 306 nm	20.00	Δ^9-THC, CBD, CBN, CBDA, Δ^9-THCA	2018, DAB<br>2018, Ph.Helv.	[95,96]

Sample type/matrix/ amount (in grams or concentration)	Instrument type and column	LC conditions: mobile phases, MPGP (A%/B%/C%), FR, IV, CT	compounds MS data (quantification ion/MRM), WL	Runtime (min)	LOD/LOQ (ng/mL or ng/g), * - LOD/*LOQ* expressed in % (w/w)	Year, country	Reference
**cannabis SC-CO_2 extracts**	Prominmence LC-2030c 3D UHPLC + DAD<br>Shim-pack XR-ODSII RP C_{18} (2.2 µm)	MP A: H_2O + 0.07% H_3PO_4<br>MP B: MeOH + 0.07% H_3PO_4<br>0-1 min (35/65) to (28/72) in 25 min, to (5/95) in 5 min<br>FR: 1 mLmin^{-1}<br>IV: 10 µL<br>CT: 50˚C	-	32.00	Δ^9-THC 370/*1260*<br>Δ^8-THC 510/*1710*<br>CBD 340/*1130*<br>CBN 330/*1102*<br>CBDA 320/*1080*<br>Δ^9-THCA 270/*920*<br>CBC 290/*990*<br>CBG 310/*1030*<br>CBGA 320/*1060*<br>THCV 330/*1110*<br>CBDV 420/*1410*	2021, Australia	[97]
**dried cannabis herbal material / 20.0 g**	Waters HPLC 900 + 996 PDA<br>Ace® 5 Phenyl (250 mm x 4.6 mm x 5 µm) + Nova-Pak® C_8 (20 mm x 3.9 mm)	MP A: H_2O + 0.1% TFA<br>MP B: H_2O/ACN (65:45, *v/v*) + 0.1% TFA<br>MP C: ACN<br>0 min (70/30/0), 10 min (60/40/0), 38 min (40/60/0), 40 min (5/95/0), 55 min (0/100/ 0), 74 min (70/30/0) + post-phase (63-71 min, 0/0/100)<br>FR 0.9 mL/min<br>IV: 10 µL/30 µL<br>CT: 25°C	214 nm	80.00	-<br>(fingerprinting method)	2017, United Kingdom	[98]
	Waters HPLC 900 + 996 PDA<br>Zorbax RX-C_{18} (250 mm x 4.6 mm x 5 µm)	MP A: H_2O/ACN (65:45, *v/v*) + 0.1% TFA<br>MP B: ACN<br>0 min (70/30), 30 min (35/65), 48 min (70/30), 40 min (5/95)	/	55.00			
**no real samples**	HPLC Agilent 1260 + QTRAP<br>Agilent Eclipse Plus 95A C_{18} (100 mm x 4.6 mm x 3.5 µm) with guard column	MF A: H_2O + 0.1% HCOOH<br>MF B: ACN + 0.1% HCOOH<br>isocratic, 10:90, *v/v*<br>FR: 0.5 mLmin^{-1}<br>IV: 20 µL<br>CT: 40°C	ESI (-)<br>CBDA 357.0 → 339.0<br>357.0 → 179.0<br>Δ^9-THCA 357.0 → 313.0<br>357.0 → 245.0<br>ESI (+)<br>CBD, Δ^9-THC	11.00	CBD 0.048<br>Δ^9-THC 0.048<br>CBDA 0.024<br>Δ^9-THCA 0.024	2018, Canada	[99]

Sample type/matrix/ amount (in grams or concentration)	Instrument type and column	LC conditions: mobile phases, MPGP (A%/B%/C%), FR, IV, CT	compounds MS data (quantification ion/MRM), WL	Runtime (min)	LOD/LOQ (ng/mL or ng/g), * - LOD/*LOQ* expressed in % (w/w)	Year, country	Reference
			315.0 → 193.0<br>315.0 → 259.0				
**dried cannabis female flowers / 0.1 g**	Thermo Scientific UHPLC + Q Exactive™ Orbitrap<br>Kinetex C_{18} (150 mm × 2.1 mm x 2.6 µm) + guard column (0.5 µm depth filter × 0.1 mm)	MP A: Milli Q + 0.1% CH_3COOH<br>MP B: CAN + 0.1% CH_3COOH<br>MP C: MeOH<br>0 min (45/50/5), 2 min (28/67/5), 6 min (28/67/5), 10 min (5/90/5), 14 min (5/90/5),<br>15 min (45/50/5), 20 min (45/50/5)<br>FR: 0.3 mLmin^{-1}<br>IV: 1 µL<br>CT: 30°C	ESI (-)<br>full scan mode (150-500 *m/z*)<br>MS/MS mode	20.00	Δ^9-THCA *5.0*<br>CBDA *1.0*<br>Δ^9-THC *2.0*<br>CBDVA *0.25*<br>CBD *1.0*<br>CBGA *2.5*<br>CBG *2.0*<br>CBDV *1.25*<br>CBC *0.25*<br>THCV *0.25*<br>CBN *1.25*<br>Δ^8-THC *1.25*<br>CBL *1.25*	2018, Israel	[100]
**cannabis extract microdepots**	Thermo Scientific UHPLC + Q-Exactive™ Orbitrap<br>Halo C_{18} (150 mm × 2.1 mm x 2.7 µm) + guard (5 mm x 2.1 mm)	MP A: Milli Q + 0.1% CH_3COOH<br>MP B: ACN + 0.1% CH_3COOH<br>MP C: MeOH<br>0 min (45/50/5), 2 min (28/67/5), 6 min (28/67/5), 10 min (5/90/5), 14 min (5/90/5),<br>15 min (45/50/5), 20 min (45/50/5)<br>FR: 0.25 mLmin^{-1}<br>IV: 5 µL<br>CT: 30°C	ESI (-)<br>full scan mode (150-500 *m/z)*	20.00	Δ^9-THC, Δ^9-THCA, CBDA, CBD, CBG, CBGA, CBC, CBN, CBDV	2020, Israel	[101]
	Thermo Scientific UHPLC + Q<br>Kinetex C_{18} (150 mm × 2.1 mm x 2.6 µm) + SecurityGuard Ultra (2 mm × 2.1 mm)	MP A: Milli Q + 0.1% CH_3COOH<br>MP B: ACN + 0.1% CH_3COOH<br>MP C: MeOH<br>0 min, at 0.8 mLmin^{-1} (45/50/5), 4 min, at 0.8 mLmin^{-1} (45/50/5), 6 min, 0.3 mL/min (28/67/5), 10 min, at 0.3 mLmin^{-1} (28/67/5), 14 min at 0.3 mLmin^{-1} (5/90/5), 18 min at 0.3 mLmin^{-1} (5/90/5), 18 min at 0.8	ESI (-)<br>SIM mode	24.00			

Sample type/matrix/ amount (in grams or concentration)	Instrument type and column	LC conditions: mobile phases, MPGP (A%/B%/C%), FR, IV, CT	compounds MS data (quantification ion/MRM), WL	Runtime (min)	LOD/LOQ (ng/mL or ng/g), * - LOD/*LOQ* expressed in % (w/w)	Year, country	Reference
		mLmin^{-1} (45/50/5), 24 min at 0.8 mLmin^{-1} (45/50/5)<br>FR: 0.25 mLmin^{-1}<br>CT: 30°C<br>IV: 5 µL					
**dried cannabis leaves and female inflorescences / 0.1 g**	Agilent 1200 LC+ 6540 UHD QTOF<br>RP-C_{18} (250 mm x 4.6 mm x 3 µm)	MP A: H_2O + 5% ACN + 0.1% HCOOH<br>MP B: ACN + 5% H_2O + 0.1 % HCCOH<br>0 min (96/4), 1 min (96/4), 5 min (80/20), 10 min (30/70), 20 min (10/90), 32 min (0/100), 42 min (0/100) + post-time re-equilibration, 10 min (96/4)<br>FR: 0.7 mL/min<br>IV: 5 µL<br>CT: 34ºC	ESI (+/-)<br>full scan mode (60-1100 *m/z)*<br>MS/MS mode<br>CBDVA 329.1758<br>CBDV 287.1992<br>CBDA 357.2072<br>CBG 317.2480<br>CBGA 359.2227<br>CBD 315.2331<br>THCV 287.1992<br>CBN 309.1863<br>Δ^9-THC 315.2300<br>Δ^8-THC 315.2316<br>CBC 315.2303<br>CBL 313.2173<br>Δ^9-THCA 357.2071	52.00	-	2019, Spain	[88]
**dried cannabis leaves and flowers (mixed) / 0.1 g**	Waters 1515® HPLC + DAD<br>Nucleodur® C_{18} Gravity (250 mm x 4.6 mm x 5 µm)	MPA: 50mM o-H_3PO_4 in H_2O<br>MP B: ACN<br>isocratic, 15:85 v/v<br>FR: 1-3 mLmin^{-1}<br>CT: 35°C	Δ^9-THC 211 nm<br>Δ^9-THCA 220 nm	5.00	Δ^9-THC 4540/*15130*	2019, Switzerland	[102]
**dried cannabis inflorescences / 0.025g**	HPLC Prominence-i LC2030C + UV<br>RP-C_{18} Nex-Leaf CBX Potency (150 mm x 4.6 mm x 2.7 µm) + NexLeaf	MP A: H_2O + 0.085% o-H_3PO_4<br>MP B: ACN + 0.085% o- H_3PO_4<br>0 min (30/70), 3 min (30/70), 7 min (15/85), 7.01 min (15/85), 8 min (5/95), 10 min (30/70)<br>FR: 1.6 mLmin^{-1}	220 nm	8.00	CBDA 340/*1050*<br>CBGA 320/*980*<br>CBG 620/*1870*<br>CBD 630/*1910*<br>THCV 950/*2870*<br>CBN 280/*840*<br>Δ^9-THC 1250/*3790*	2019, Italy	[103]

**Sample type/matrix/ amount (in grams or concentration)**	**Instrument type and column**	**LC conditions: mobile phases, MPGP (A%/B%/C%), FR, IV, CT**	**compounds MS data (quantification ion/MRM), WL**	**Runtime (min)**	**LOD/LOQ (ng/mL or ng/g), * - LOD/*LOQ* expressed in % (w/w)**	**Year, country**	**Reference**
	CBX (5 mm x 4.6 mm x 2.7 µm)	IV: 5 µL<br>CT: 35°C			Δ^8-THC 1020/*3100*<br>CBC 290/*880*<br>Δ^9-THCA 430/1290		
**dried cannabis inflorescences / 2.5 g**	Agilent 1100 HPLC + MSD trap (SL)<br>Poroshell 120 SB-C_{18} (75 mm x 3.0 mm x 2.7 µm)	MP A: 25 mM NH4CH2COOH<br>MP B: MeOH<br>0 min (32/68), 9 min (15/85), 10 min (32/68)<br>FR: 0.7 mLmin^{-1}<br>IV: 10 µL<br>CT: 30°C	235 nm	10.00	CBDA 62.5/*250*<br>CBGA 62.5/*250*<br>CBG 62.5/*250*<br>CBD 62.5/*250*<br>THCV 62.5/*250*<br>CBN 62.5/*250*<br>Δ^9-THC 62.5/*250*<br>Δ^8-THC 62.5/*250*<br>CBC 62.5/*250*<br>Δ^9-THCA 62.5/*250*	2017, USA	[104]
**fresh cannabis female inflorescences / 0.25 g**	Agilent 1100 HPLC + UV/DAD Ascentis Express C_{18} (150 mm x 3.0 mm x 2.7 µm)	MP A: 0.1% HCOOH in $H_2$0<br>MP B: 0.1% HCOOH in ACN<br>0 min (40/60), 13 min (40/60), 17 min (20/80), 22 min (10/90)<br>FR: 0.4 mLmin^{-1}<br>IV: 3 µL<br>CT: 30°C	210 nm, 220 nm	37.00	CBDA, CBGA, CBD, CBG	2018, Italy	[105]
	Agilent 1100 HPLC + IT Ascentis Express C_{18} (150 mm x 3.0 mm x 2.7 µm)		ESI (+/-)<br>full scan mode (200-1200 *m/z*)<br>MS/MS mode (50-1500 *m/z*)<br>CBDA 359, 341/ 357*<br>CBGA 361, 343 / 359*<br>CBG 317/315*<br>CBD 315/313*<br>*(-) mode	37.00	-		
**dried cannabis plant material (recreational, medical, hemp) / consumer products (oral supplements, foods, candies, beverages, vapes, liquids, topicals) /**	Agilent 1100, 1200, or 1260 HPLC + DAD<br>MacMod Ace® 5 C_{18}-AR (250 mm x 4.6 mm x 5 µm)	MP A: 0.5% CH_3COOH<br>MP B: ACN<br>isocratic, 34:66, *v/v*<br>FR: 1 mLmin^{-1}<br>IV: 25 µL	220 nm<br>240 nm<br>270 nm<br>307 nm	50.00	CBD, CBDA, Δ^9-THC, THCA, CBN, Δ^8-THC, CBG, CBGA, CBDV, THCV, CBC<br>concentration-based 200/*500*<br>method-based *10000*	2018, USA	[106]

Sample type/matrix/ amount (in grams or concentration)	Instrument type and column	LC conditions: mobile phases, MPGP (A%/B%/C%), FR, IV, CT	compounds MS data (quantification ion/MRM), WL	Runtime (min)	LOD/LOQ (ng/mL or ng/g), * - LOD/*LOQ* expressed in % (w/w)	Year, country	Reference
**medical products / illicit products (kief, hash oil) / 0.03 – 3.0 g**							
**hexane (+0.1% HCOOH) cannabis inflorescence extracts**	Agilent 1100 HPLC + G1315 DAD + 6320 IT<br>Luna Omega PS C_{18} (150 mm x 2.1 mm x 5 μm)	MPA: H_2O + 0.1% HCOOH<br>MPB: ACN<br>0 min (50/580) + 6 min, 12 min (43/57), 23 min (50/50) + 2 min<br>FR: 0.4 mLmin^{-1}<br>IV: 5 μL<br>CT: 28°C	(190-6020 nm for UV-Vis spectra acquisition)<br>220 nm<br>ESI (+) and (-)<br>SIM mode<br>ESI (-)<br>CBDA **357**, 339, 245<br>CBGA **359**, 341<br>CBNA **353**, 309, 279<br>THCAA **357**, 313, 245<br>ESI (+)<br>CBG **317**, 207, 233<br>CBD **315,** 259, 233<br>CBN **311**, 223, 43<br>Δ^9-THC **315**, 245, 193	25.00	-	2021, Italy	[107]
**cannabis tinctures/oils / 0.5 mL<br>beverages / 1.0 mL<br>powders, edibles. gummies and candies / 1.0 g**	Agilent 1290 HPLC + DAD/FLD<br>ACE Excel 3 C_{18} (150 mm x 2.1 mm x 3.0 μm)	MP A: 0.5% CH_3COOH<br>MP B: ACN<br>0 min (33/67), 17 min (5/95) + re-equilibration 18 min (33/67)<br>FR: 0.3 mL<br>IV: 2 μL<br>CT: 25°C	DAD:<br>220 nm<br>240 nm<br>270 nm<br>307 nm<br>FLD:<br>234/311 nm (0-8.5 min)<br>261/378 nm (8.5-11.0 min)<br>234/315 nm (11.0-13.5 min)<br>272/346 nm (13.5-24.0 min)	24.00	CBD, CBDA, Δ^9-THC, THCA, CBN, Δ^8-THC, CBG, CBGA, CBDV, THCV, CBC	2021, USA	[108]
**CBD e-liquids**	Shimadzu HPLC + Applied	MP A: DI H_2O<br>MP B: MeOH<br>isocratic, 90:10, *v/v*	CBD 315>193, 315>259	8.00	CBD	2016, USA	[109]

Sample type/matrix/ amount (in grams or concentration)	Instrument type and column	LC conditions: mobile phases, MPGP (A%/B%/C%), FR, IV, CT	compounds MS data (quantification ion/MRM), WL	Runtime (min)	LOD/LOQ (ng/mL or ng/g), * - LOD/*LOQ* expressed in % (w/w)	Year, country	Reference
	Bioscience 3200 QTRAP<br>Zorbax Eclipse XDBC$_{18}$ (75 mm x 4.6 mm x 3.5 μm)	FR: 0.5 mLmin^{-1}<br>IV: 10 μL					
**fresh cannabis plant material / 0.05 g**	Agilent HPLC Infinity + Agilent 6430 QQQ<br>Kinetex C$_{18}$ (150 mm x 3 mm x 2.6 μm) + guard column (0.5 μm depth filter x 0.1 mm)	MP A: H_2O + 0.1% HCOOH<br>MF B: MeOH + 0.1% HCOOH<br>0 min (50/50), 1 min (20/80), 11 min (20/80), 13 min 5 (5/95), 16 min (5/95), 18 min (50/50), 28 min (50/50)<br>FR: 0.25 mLmin^{-1}<br>IV: 10 μL<br>CT: 30°C	APCI (+)<br>MRM mode<br>CBD 315.1 → 192.8<br>315.1 → 259.0<br>THCV 287.1 → 165.0<br>287.1 → 231.0<br>CBG 317.2 → 193.2<br>317.2 → 123.0<br>CBN 311.0 → 222.9<br>311.0 → 293.0<br>Δ^9-THC 315.0 → 193.0<br>315.0 → 259.0<br>Δ^9-THCA 315.1 → 193.0<br>315.1 → 259.1	28.00	CBD 0.2<br>THCV 0.05<br>CBG 0.02<br>CBN 0.05<br>Δ^9-THC 0.05<br>Δ^9-THCA 0.02	2014, Spain	[110]
	Waters ACQUITY UPLC + SYNAPT G2 QTOF<br>Kinetex C$_{18}$ (150 mm x 3 mm x 2.6 μm) + guard column (0.5 μm depth filter x 0.1 mm)		APCI (+)<br>untargeted analysis	22.00	CBD, THCV, CBG, CBN, Δ^9-THC, Δ^9-THCA		
	Waters Thar SFC s	MP: SC-CO_2 + MeOH (15%)<br>FR: 1.5 mLmin^{-1}<br>IV: 5 μL<br>CT 40°C	220 nm	7.00			

Sample type/matrix/ amount (in grams or concentration)	Instrument type and column	LC conditions: mobile phases, MPGP (A%/B%/C%), FR, IV, CT	compounds MS data (quantification ion/MRM), WL	Runtime (min)	LOD/LOQ (ng/mL or ng/g), * - LOD/*LOQ* expressed in % (w/w)	Year, country	Reference
	Kromasil NP-DIOL (250 mm x 4.6 mm x 5 µm)						
**fresh cannabis extracts / 0.05 g**	Waters UHPSFC + PDA + Q<br>ACQUITY UPC² BEH 2-EP (150 mm x 3.0 mm x 1.7 µm)	MP A: SC-CO_2<br>MP B: isopropanol/ACN (80:20, *v/v*) + 1% H_2O<br>0 min (96/4), 4.5 min (91/9), 7.0 min (70/30), 10 min (70/30)<br>FR: 1.4 mLmin^{-1}<br>IV: 1.0 µL<br>CT: 30°C	scan mode (19-400 nm)<br>220 nm	16.50	CBD, THCV, CBG, CBN, Δ^9-THC, Δ^9-THCA, CBDA, CBGA	2016, USA	[111]
**cannabis seed oil / 0.1 mL**	Agilent HPLC 1200 + DAD Poroshell 120 EC-C_{18} (100 mm x 3.0 mm x 2.7 µm)	MP A: H_2O + 0.1% HCOOH<br>MP B: ACN + 0.1% HCCOH<br>0 min (30/70), 10 min (20/80), 10.1 min (5/95), 11.0 min (5/95), 11.1 min (30/70), 15 min (30/70)<br>FR: 0.4 mLmin^{-1}<br>IV: 5 µL<br>CT: 25°C	228 nm	15.00	CBDV, CBDA, CBG, CBD, CBN, Δ^9-THC, Δ^9-THCA	2018, Italy	[112]
	Agilent HPLC 1200 + 6540 QTOF Poroshell 120 EC-C_{18} (100 mm x 3.0 mm x 2.7 µm)		ESI (+/-)<br>full scan mode (50-700 *m/z*)<br>MS/MS mode, (+)<br>CBDA 359.2217<br>Δ^9-THCA 359.2217<br>CBD 315.2300<br>Δ^9-THC 315.2300<br>CBDV 287.1998<br>CBG 317.2468<br>CBN 311.2024<br>MS/MS mode, (-)<br>CBDA 357.2164<br>Δ^9-THCA 357.2164<br>CBD 313.2012<br>Δ^9-THC 313.2012<br>CBDV 285.1830<br>CBG 315.2385	15.00			

Sample type/matrix/ amount (in grams or concentration)	Instrument type and column	LC conditions: mobile phases, MPGP (A%/B%/C%), FR, IV, CT	compounds MS data (quantification ion/MRM), WL	Runtime (min)	LOD/LOQ (ng/mL or ng/g), * - LOD/*LOQ* expressed in % (w/w)	Year, country	Reference
			CBN 309.1902				
**fresh cannabis female inflorescences / 0.25 g cannabis oil / 50 µL cannabis balm/ 0.25 g cannabis extract / 0.02 g**	Agilent HPLC 1100 + UV Ascentis Express C_{18} (150 mm x 3.0 mm x 2.7µm)	MP A: H_2O + 0.1% HCOOH<br>MP B: ACN + 0.1% HCOOH<br>0 min (40/60), 13 min (40/60), 17 min (20/80), 22 min (10/90), 30 min (10/90)<br>FR: 0.4 mLmin^{-1}<br>IV: 3 µL<br>CT: 30°C	210 nm<br>220 nm	45.00	CBDA 800/*2500*<br>CBGA 800/*2500*<br>CBG 500/*1800*<br>CBD 700/*1300*	2017, Italy	[113]
	Agilent HPLC 1200 + 6310A IT<br>Ascentis Express C_{18} (150 mm x 3.0 mm x 2.7µm)		ESI (+/-)<br>full scan mode (200-1200 *m/z*)<br>MS/MS mode (50-1500 *m/z*)		-		
**multi-floral / dandelion / chestnut honey / 20.0 g fresh cannabis male inflorescences and their pollen / 0.25 g**	Agilent 1100 HPLC + UV/DAD Ascentis Express C_{18} (150 mm x 3 mm x 2.7 µm)		210 nm<br>220 nm	32.00	CBDA 0.3/*0.5*<br>CBGA 0.3/*0.5*<br>Δ^9-THCA 0.3/*0.5*<br>CBG 0.3/*0.5*<br>CBD 0.3/*0.5*<br>Δ^9-THC 0.3/*0.5*	2019, Italy	[114]
	Agilent 1200 HPLC + AB SCIEX API 4000 QTRAP<br>Kinetex EVO C_{18} (100 mm x 2.1 mm x 5 µm)	MP A: 2 mM $CH_3COOHNH_4$ in H_2O<br>MF B: 2 mM $CH_3COOHNH_4$ in ACN<br>0 min (70/30), 10 min (10/90), 15 min (10/90), 18 min (70/30)<br>FR: 0.35 mLmin^{-1}<br>IV: 25 µL<br>CT: 40°C	ESI (-)<br>MRM mode<br>CBDA 357 → **245**, 179, 271<br>CBGA 359 → **341**, 315, 217<br>Δ^9-THCA 357 → **191**, 245<br>CBG 315 → **136**, 191, 177<br>CBD 313 → **245**, 107<br>Δ^9-THC 313 → **245**, 191, 203				
**foods, beverages and feeds / 1.0 g**	Agilent 1200 HPLC + AB SCIEX API 4000 QTRAP<br>Ascentis Express RP-amide (50 mm	MPA: H_2O + 0.1% HCOOH<br>MPB: ACN + 0.1% HCOOH<br>0-10.0 min (60/40), 19.0 min (5/95), 22.0 (60/40) + re-equilibration 7 min<br>FR: 0.8 mLmin^{-1}	ESI (+) and (–)<br>MRM mode<br>(+)<br>Δ^9-THC 315.4 → 193.3<br>315.4 → 259.4<br>Δ^8-THC 315.4 → 193.3<br>315.4 → 259.4	22.00	CBD, CBN, CBG, Δ^8-THC, Δ^9-THC, THCV, CBDA, CBGA, Δ^9-THCA<br>honey, coffee and eggs: 6.0/*20.0*<br>beverages: 0.6/*2.0*	2021, Italy	[115]

Sample type/matrix/ amount (in grams or concentration)	Instrument type and column	LC conditions: mobile phases, MPGP (A%/B%/C%), FR, IV, CT	compounds MS data (quantification ion/MRM), WL	Runtime (min)	LOD/LOQ (ng/mL or ng/g), * - LOD/*LOQ* expressed in % (w/w)	Year, country	Reference
	x 4.6 mm x 2.7 µm)	IV: 100 µL<br>CT: 25°C	CBD 315.5 → 193.2<br>315.5 → 259.1<br>CBN 315.5 → 223.2<br>315.5 → 241.2<br>CBG 317.3 → 193.4<br>317.3 → 123.4<br>THCV 287.4 → 165.3<br>287.4 → 135.3<br>(-)<br>THCA 357.5 → 313.5<br>357.5 → 245.2<br>CBDA 357.5 → 245.4<br>357.5 → 339.5<br>CBGA 359.4 → 341.5<br>359.4 → 315.5		feed: 30.0/*100.0*		
**cannabis extracts / 2.0 g, 5.0 g, 20.0 g (depending on extraction methodology)**	Agilent HPLC 1200 + UV Poroshell 120 SB-C_{18} (100 mm x 2.1 mm x 2.7 µm)	MP A: H_2O + 0.1% HCOOH<br>MP B: ACN + 0.1% HCOOH<br>isocratic<br>FR: 0.5 mL/min<br>IV: 5 µL<br>CT: 25°C	228 nm	10.00	CBDA<br>CBD, CBN, Δ⁹-THC, Δ⁹-THCA	2016, Italy	[116]
	Agilent HPLC 1200 + 6540 QTOF Poroshell 120 SB-C_{18} (100 mm x 2.1 mm x 2.7 µm)		ESI (+)<br>full scan mode (50-500 *m/z*)<br>MS/MS mode (50-1700 *m/z*)<br>CBDA 359.2224<br>CBD 315.2314<br>CBN 311.2000<br>Δ⁹-THC 315.2311<br>Δ⁹-THCA 359.2216	10.00	CBDA, CBD, CBN, Δ⁹-THC, Δ⁹-THCA		
**dried cannabis plant material / 0.3 g marihuana / 0.5 g**	HPLC Waters 2695 + LiChrospher 60, RP-Select B LiChroCart (125 mm x 4 mm x 5 µm) +	MP A: 1M triethylammoniumphosphate in Milli-Q<br>MP B: ACN<br>isocratic, 36:64, *v/v*<br>FR: 1 $mLmin^{-1}$	210 nm	~16.00	Δ⁹-THC 1000/*6000*<br>Δ⁹-THCA 4000/*16000*<br>CBD 1000/*40000*<br>CBN 1000/*40000*	2014, Switzerland	[117]

Sample type/matrix/ amount (in grams or concentration)	Instrument type and column	LC conditions: mobile phases, MPGP (A%/B%/C%), FR, IV, CT	compounds MS data (quantification ion/MRM), WL	Runtime (min)	LOD/LOQ (ng/mL or ng/g), * - LOD/*LOQ* expressed in % (w/w)	Year, country	Reference
	LiChrospher 60, RP- Select B (5 μm)	IV: 10 μL					
**capsule, oil tincture, soft chew or powder / 0.02 g, 0.1 g or 1.0 g**	Acquity UPLC + DAD	-	190-500 nm	-	CBGA, CBG, CBDA, CBD, Δ9-THCA, Δ9-THC, CBN, exo-THC, Δ8-THC, CBC, THCV, CBDV	2020, USA	[118]
**fresh cannabis inflorescences, cannabis tea, cannabis oil / 0.5 g**	Waters Acquity UPLC + QQQ<br>Acquity UPLC HSS C_{18} (150 mm x 2.1 mm x 1.8 μm)	MP A: H_2O + 0.1% HCOOH<br>MP B: ACN<br>0 min (40/60), 0.5 min (40/60), 4.5 min (10/90), 6.5 min (10/90), 7.0 min (40/60), 10 min (40/60)<br>FR: 0.4 mLmin^{-1}<br>IV: 30 μL<br>CT 30°C	ESI (+)<br>CBDA 359.4 → **219.3**, 261.3<br>CBG 317.5 → **193.3**, 123.2<br>CBD 315.4 → **193.3**, 123.2<br>CBN 311.4 → **223.3**, 293.4<br>Δ9-THC 315.4 → **123.2**, 193.3<br>CBC 315.4 → **193.3**, 123.2<br>Δ9-THCA 359.4 → **219.3**, 261.3	10.00	CBDA, CBG, CBD, CBN, Δ9-THC, CBC, Δ9-THCA	2017, Italy	[119]
**dried cannabis plant material / 0.1 g**	HPLC + DAD<br>Luna C_{18} (150 mm x 4.60 mm x 3 μm) + C_{18} guard column cartridge	MP A: H_2O + 0.1% HCOOH<br>MP B: ACN + 0.1% HCCOH<br>0 min (30/70), 6 min (30/70), 12 min (23/77), 22 min (23/77), 22.2 min (30/70)<br>FR: 1.2 mLmin^{-1}<br>IV: 10 μL<br>CT: 28°C	220 nm	22.50	CBDA 100/*18400*<br>CBGA 1300/*3900*<br>CBG 2500/*7700*<br>CBD 1600/*4900*<br>THCV 700/*2100*<br>CBN 300/*1000*<br>Δ9-THC 2300/*6900*<br>Δ8-THC 1400/*4200*<br>CBL 1100/*3400*<br>CBC 1100/*3400*<br>Δ9-THCA 1100/*3400*	2015, Egypt	[120]

Sample type/matrix/ amount (in grams or concentration)	Instrument type and column	LC conditions: mobile phases, MPGP (A%/B%/C%), FR, IV, CT	compounds MS data (quantification ion/MRM), WL	Runtime (min)	LOD/LOQ (ng/mL or ng/g), * - LOD/*LOQ* expressed in % (w/w)	Year, country	Reference
**cannabis inflorescences / 0.1 g** [121]<br><br>**cannabis plant material, cannabis oil / 0.1 g** [122]<br><br>**dried cannabis inflorescences / 1.0 g** [123]	Thermo Fisher HPLC + Q-Exactive Orbitrap®<br>Synergi Hydro RP (150 mm x 2 mm x 4.0 µm) + C_{18} (4 mm x 3 mm)	MP A: H_2O + 0.1% HCOOH<br>MP B: ACN + 0.1% HCOOH [121,123]<br>0 min (95/5), 35 min (5/95) [121,123]<br>MP A: H_2O + 0.1% HCOOH<br>MP B: ACN<br>0 min (60/40), 10 min (95/5), 14 min (95/5) [122,124]<br>FR: 0.3 $mLmin^{-1}$<br>IV: 2 µL<br>CT: 30°C	ESI (+/-)<br>full scan mode (100-900 *m/z*)<br>MS/MS mode [121,123]<br>full scan mode (215-500 *m/z*)<br>MS/MS mode [122,124]	35.00 [121,123]<br><br>20 min [122,124]	CBD, Δ⁹-THC, CBN, CBG, CBC, CBDV, THCV, CBDA, Δ⁹-THCA, CBNA, CBGA, CBCA, CBDVA, THCVA [121,123]<br>CBD *0.1*<br>Δ⁹-THC *0.1*<br>CBN *0.1*<br>CBG *0.1*<br>CBDA *0.05*<br>THCA *0.05*<br>CBGA *0.05*<br>[122]<br>CBD, ΔTHC, CBN, CBG, CBDA, Δ⁹-THCA, CBGA [124]	2019, Italy<br>2018, Italy<br>2020, Italy<br>2018, Italy	[121]<br>[122]<br>[123]<br>[124]
**dried cannabis inflorescences, leaves, stem barks and roots / 2.0-4.0 g**	Agilent 1260 Infinity II + Q<br>Zorbax RX-C_{18} (150 mm x 4.6 mm x 3.5 µm)	MP A: H_2O + 0.2% HCOOH<br>MP B: MeOH<br>0 min (25/75), 13 min (10/90), 26 min (10/90)<br>FR: 0.6 $mLmin^{-1}$<br>IV: 5 µL<br>CT: 30°C	ESI (+)<br>CBDV 287.2<br>CBDVA 331.2<br>CBG 317.3<br>CBD 315.3<br>CBDA 359.2<br>THCV 287.2<br>CBGA 343.3 CBN 311.2<br>Δ⁹ -THC 315.3<br>Δ⁸ -THC 315.3<br>THCVA 331.2<br>CBC 315.2<br>Δ⁹-THCA 359.3<br>CBCA 359.3	30.00	CBDV 1.0/*3.0*<br>CBDVA 2.0/*5.0*<br>CBG 2.0/*5.0*<br>CBDA 1.0/*3.0*<br>THCV 0.4/*1.0*<br>CBGA 3.0/*8.0*<br>CBN 1.0/*2.0*<br>Δ⁹-THC 1.0/*3.0*<br>Δ⁸-THC 2.0/*5.0*<br>THCVA 3.0/*10.0*<br>CBC 2.0/*7.0*<br>Δ⁹-THCA 3.0/*9.0*<br>CBCA 4.0/*12.0*	2020, Canada	[125]

Sample type/matrix/ amount (in grams or concentration)	Instrument type and column	LC conditions: mobile phases, MPGP (A%/B%/C%), FR, IV, CT	compounds MS data (quantification ion/MRM), WL	Runtime (min)	LOD/LOQ (ng/mL or ng/g), * - LOD/*LOQ* expressed in % (w/w)	Year, country	Reference
**galenic cannabis oils/ 10 µL or 50 µL**	Acquity® UPLC + TQD<br>AcquityHSS-T3 (30 mm x 2.1 mm x 1.8 µm)	MPA: H_2O/ACN (30/70) + 0.05% HCOH<br>MPB: isopropanol/ACN (80/20) + 0.05% HCOH<br>0 min (100/0), 4.6 min (0/100) + 1.5 min + reconditioning at (0/100)<br>FR: 0.4 mLmin^{-1}<br>IV: 4 µL	ESI (+)<br>SRM mode<br>THC 315.2 → 193.1<br>CBD 315.2 → 259.2<br>CBDA 359.15 → 261.1<br>THCA 341.15 → 219.15<br>THC-d_3 318.2 → 196.1<br>CBD-d_3 318.2 → 296.5	7.00	-	2021, Italy	[126]
	Agilent 1200 HPLC + 6430 QQQ<br>Zorbax Eclipse Plus C_{18} (50 mm x 2.1 mm x 1.8 µm)	MPA: H_2O + 0.1% HCOOH in ACN<br>MPB: 0.1% HCOOH in ACN<br>0 min (50/50), 2.8 min (0/100) + 2 min + reconditioning at (50/50)<br>FR: 0.3 mLmin^{-1}<br>IV: 5 µL	ESI (+)<br>SRM mode<br>THC 315.2 → 123.0<br>CBD 315.2 → 193.1<br>CBDA 357.2 → 245.1<br>THCA 357.2 → 245.1<br>THC-d_3 318.2 → 196.1<br>CBD-d_3 262.0 → 196.1	8.50			
**dried cannabis plant material / 0.1 g**	Agilent Infinity 1290 UHPLC + 6430 QQQ<br>Zorbax Eclipse Plus C_{18} Rapid Resolution	MPA: H_2O + 5 mM ammonium formate<br>MPB: ACN + 0.1% HCOOH<br>0 min (35/65), 4.5 min (20/80), 7.5 min (19.1/80.90), 9.5 min (0/100) + re-equilibration<br>FR: 0.6 mLmin^{-1}<br>IV: 5 µL<br>CT: 30°C	ESI (+) and (-)<br>MRM mode<br>ESI (+)<br>CBG-d_9 **326.3 → 202.2**<br>326.3 → 123.0<br>CBG **317.3 → 193.1**<br>317.3 → 123.0<br>CBD-d_3 **318.3 → 196.1**<br>318.3 → 123.0<br>CBD **315.2 → 193.1**<br>315.2 → 123.0<br>THCV **287.2 → 123.0**<br>287.2 → 231.1<br>THC-d_3 **318.3 → 196.1**<br>318.3 → 123.0<br>THC **315.2 → 193.1**<br>315.2 → 123.0<br>CBL **315.2 → 235.2**	14.00	THC 0.014/*10.0*<br>THCA 0.01/*10.0*<br>CBD 0.2/*0.5*<br>CBDA 0.04/*0.4*<br>CBN 0.25/*0.25*<br>CBNA 0.005/*1.0*<br>CBG 1.0/*1.0*<br>CBGA 0.1/*1.0*<br>CBC 0.5/*0.5*<br>CBCA 0.1/*10.0*<br>CBL 0.2/*0.2*<br>CBLA 0.013/*1.0*<br>THCV 0.25/*0.5*<br>THCVA 0.005/*1.0*<br>CBDV 0.5/*1.25*<br>CBDVA 0.02/*0.1*	2021, Germany	[127]

Sample type/matrix/ amount (in grams or concentration)	Instrument type and column	LC conditions: mobile phases, MPGP (A%/B%/C%), FR, IV, CT	compounds MS data (quantification ion/MRM), WL	Runtime (min)	LOD/LOQ (ng/mL or ng/g), * - LOD/*LOQ* expressed in % (w/w)	Year, country	Reference
			315.2 → 165.1<br>CBC-d9 **324.3 → 202.2**<br>324.3 → 268.2<br>CBC **315.2 → 193.1**<br>315.2 → 259.2<br><br>ESI (-)<br>CBDA **329.2 → 311.2**<br>329.2 → 217.1<br>THC-COOH-d9<br>**352.3 → 308.3**<br>352.3 → 254.3<br>CBDV **285.2 → 217.1**<br>285.2 → 107.0<br>CBDA **357.2 → 339.2**<br>357.2 → 245.2<br>CBGA **359.2 → 341.2**<br>359.2 → 315.2<br>THCVA **329.2 → 285.2**<br>329.2 → 217.1<br>CBN-d3 **312.2 → 282.1**<br>312.2 → 222.1<br>CBN **309.2 → 279.1**<br>309.2 → 222.1<br>CBNA **353.2 → 309.2**<br>353.2 → 279.1<br>CBC-d9 **324.3 → 200.2**<br>324.3 → 268.2<br>THCA **357.2 → 313.2**<br>357.2 → 245.2<br>CBCA **357.2 → 313.2**<br>357.2 → 191.1<br>CBLA **357.2 → 313.2**<br>357.2 → 191.1				
**dried cannabis plant material**	ThermoFisher UltiMate® 3000	MPA: H_2O + 0.1% HCOOH<br>MPB: ACN + 0.1% HCOOH	ESI (+)<br>MRM mode	15.00	CBD 0.20/*0.61*<br>CBN 0.03/*0.09*<br>THC 0.06/*0.17*	2021, South Africa	[128]

Sample type/matrix/ amount (in grams or concentration)	Instrument type and column	LC conditions: mobile phases, MPGP (A%/B%/C%), FR, IV, CT	compounds MS data (quantification ion/MRM), WL	Runtime (min)	LOD/LOQ (ng/mL or ng/g), * - LOD/*LOQ* expressed in % (w/w)	Year, country	Reference
	HPLC + Bruker Compact QTOF<br>C_{18} Wate (100 mm x 4.6 mm x 3.5 μm) + guard column	-<br>FR: 0.3 $mLmin^{-1}$<br>IV: 10 μL<br>CT: 30°C					
**lyophilized non-drug-type cannabis plant material**	HP HPLC 1050 + G1315B DAD<br>Luna C_{18} (150 mm x 2 mm x 3 μm)	MPA: 5% ACN in H_2O + 0.1% o-H_3PO_4<br>MPB: 80% CAN in H_2O + 0.1% o-H_3PO_4<br>isocratic (17/83, *v/v*)<br>FR: 0.25 $mLmin^{-1}$<br>IV: 5 μL<br>CT: 35°C	220 nm	15.00	CBD, CBDA, CBG 13.0/*44.0*	2021, Czech Republic	[129]
	Thermo Fischer LCQ Accela Fleet + IT<br>Luna C_{18} (150 mm x 2 mm x 3 μm)	MPA: 5% ACN in H_2O + 0.1% HCOOH<br>MPB: 80% ACN in H_2O + 0.1% HCOOH<br>isocratic (17/83, v/v)<br>FR: 0.25 $mLmin^{-1}$<br>IV: 5 μL<br>CT: 35°C	APCI (-)		-		
**cannabis plant material, cannabis resins / 0.5 g**	Acquity UPLC + PDA<br>Poroshell 120 EC C_{18} (150 mm x 2.1 mm x 2.7 μm) + Poroshell 120 EC-C_{18} guard (5 mm x 2.1 mm x 2.7 μm)	MPA: H_2O + 0.1% HCOOH<br>MPB: ACN + 0.1% HCOOH<br>0 min (32/68), 2.8 min (27/73), 7.0 min (0/95) +1.0 min + re-equilibration (4.5 min)<br>FR: 0.5 $mLmin^{-1}$<br>IV: 1 μL<br>CT: 30°C	214 nm	18.00	CBD, CBN, THC, CBDA, THCA	2020, Belgium<br>2021, Belgium	[130]

Sample type/matrix/ amount (in grams or concentration)	Instrument type and column	LC conditions: mobile phases, MPGP (A%/B%/C%), FR, IV, CT	compounds MS data (quantification ion/MRM), WL	Runtime (min)	LOD/LOQ (ng/mL or ng/g), * - LOD/*LOQ* expressed in % (w/w)	Year, country	Reference
**no real samples**	Agilent 1260 Infinity + DAD<br>ACE 3 C_{18}-PFP (150 mm x 3.0 mm x 3.0 μm)	MPA: H_2O<br>MPB: MeOH<br>isocratic, 17/83 *v/v*<br>FR: 0.4 $mLmin^{-1}$<br>IV: 5 μL<br>CT: 25°C	222 nm	20.00	CBD 25.0/*100.0*<br>CBN 25.0/*100.0*<br>Δ^9-THC50.0/*100.0*	2021, Thailand	[131]
**lyophilized cannabis flowers**	Agilent HPLC 1100 + DAD<br>Kinetex® C_{18} (150 mm x 2.1 mm x 2.6 μm)	MPA: H_2O + 0.1% TFA<br>MPB: MeOH + 0.1% TFA<br>0 min (32/68), 13 min (15/85) + 7 min<br>FR: 0.25 $mLmin^{-1}$<br>CT: 60°C	230 nm	20.00	CBG, CBGA, CBD, CBDA, CBN, Δ^9-THC, CBC, CBCA, Δ^9-THCA, THCV, CBDV, CBGVA	2021, Canada	[132]
**upper cannabis leaves / 1.5 g**	Nexera XR LC-20AD + DAD<br>Atlantis T3 C_{18} (150 mm x 4.6 mm x 3.0 μm)	MPA: ACN<br>MPB: H_2O + 0.85% phosphoric acid<br>0 min (53/47), 15 min (20/80) + 11 min, 28 min (0/100) + 5 min + re-equilibration 5 min	-	38.00	THC 810.0/*2680.0*	2021, Austria	[133]
**cannabis inflorescence / 0.05 g**<br>**cannabis oil**	Shimadzu HPLC + SPD-20A UV<br>Raptor ARC-18 (150 mm x 4.6 mm x 2.7 μm)	MPA: 5 Mm ammonium formate + 0.1% HCOOH<br>MPB: ACN + 0.1% HCOOH<br>isocratic, 25/75 *v/v*	228 nm	11.00	THCA, CBDA<br>-/ ≤ *1050*<br>Δ^9-THC, CBD -/ ≤ *5250*<br>CBDV, CBDVA, CBGA, CBG, THCV, THCVA, CBN, CBNA, Δ^8-THC, CBC, CBL, CBLA, CBCA<br>-/ ≤ *500*	2021, Australia	[134]
**dried cannabis inflorescences and inflorescence leaves / 0.05 g**	Jasco 2000 Plus HPLC + PDA	MPA: ACN<br>MPB: H_2O + 0.1% HCOOH<br>isocratic, 75/255 *v/v*<br>FR: 1.0 $mLmin^{-1}$	200-650 nm<br>CBC, CBCA, CBCV, CBG, CBGA, CBN, CBNA, CBD, CBDA, CBL, CBDV, CBDVA,	-	-	2021, Israel	[135]

Sample type/matrix/ amount (in grams or concentration)	Instrument type and column	LC conditions: mobile phases, MPGP (A%/B%/C%), FR, IV, CT	compounds MS data (quantification ion/MRM), WL	Runtime (min)	LOD/LOQ (ng/mL or ng/g), * - LOD/*LOQ* expressed in % (w/w)	Year, country	Reference
	Luna Omega Polar C_{18} (150 mm x 2.1 mm x 3 μm)		THCVA, THCA, Δ^9-THC, CBT				
**hemp pollen from industrial cannabis inflorescences**	Nexera UHPLC + Ab SCIEX TripleTOF 4600 hybrid system<br>Luna® Omega C_{18} (50 mm x 2.1 mm x 1.6 μm)	MPA: H_2O + 0.1% HCOOH<br>MPB: ACN + 0.1% HCOOH<br>0 min (75/25), 1 min (45/55), 8.5 min (5/95) + 1 min + re-equilibration<br>FR: 0.5 mLmin^{-1}<br>IV: 2 μL	ESI (-)<br>CBDA, CBCA, THCA	11.50	-	2021, Italy	[136]
**CBD cosmetic products / 1.0 g**	Agilent 1100 HPLC + Agilent 6410B QQQ<br>Zorbax SB-C_{18} (50 mm x 2.1 mm x 1.8 μm)	MPA: MeOH + 0.1% HCOOJH<br>MPB: H_2O + 0.1% HCOOH<br>isocratic, 80/20 *v/v*<br>FR: 0.2 mLmin^{-1}<br>IV: 10 μL<br>CT: 35°C	ESI (+)<br>MRM mode<br>CBD **315 → 193**<br>315 → 41<br>315 → 123<br>CBD-d_3 **318 → 196**<br>318 → 41<br>318 → 123	4.00	CBD 0.22/*0.74*	2021, Spain	[137]
**hempseed oil / 0.5 g raw milk/ 10.0 g hemp seeds, hemp proteins, tea, raw milk, skimmed powder milk, coffee and chocolate / 2.0 g**	Waters UPLC + Sciex QTRAP 6500<br>Acquity BEH Shield RP18 (100 mm x 2.1 mm x 1.7 μm)	MPA: H_2O + 0.1% HCOOH<br>MPB: ACN<br>0 min (50/50), 9.0 min (0/100), 2 min + 2.0 min re-equilibration<br>FR: 0.5 mLmin^{-1}<br>IV: 5 μL<br>CT: 40°C	ESI (+) and (-)<br>MRM mode<br>CBD **315.3 → 193.1**<br>315.3 → 259.2<br>315.3 → 135.0<br>CBDA **357.3 → 245.1**<br>357.3 → 339.2<br>357.3 → 226.9<br>CBN **311.2 → 223.1**<br>311.2 → 293.2<br>311.2 → 195.1<br>Δ^9-THC **315.3 → 193.1**<br>315.3 → 259.1<br>315.3 → 195.1<br>THCA-A **357.2 → 213.1**<br>357.2 → 245.0<br>357.2 → 191.0	13.00	hemp seeds<br>hemp protein */150.0*<br>hemp seed oil -<br>/600.0<br>raw milk<br>skimmed milk<br>-/5.0	2020, Switzerland	[138]

Sample type/matrix/ amount (in grams or concentration)	Instrument type and column	LC conditions: mobile phases, MPGP (A%/B%/C%), FR, IV, CT	compounds MS data (quantification ion/MRM), WL	Runtime (min)	LOD/LOQ (ng/mL or ng/g), * - LOD/*LOQ* expressed in % (w/w)	Year, country	Reference
			CBC **315.2 → 193.0**<br>315.2 → 259.2<br>315.2 → 123.1<br>CBCA **357.2 → 191.0**<br>357.2 → 313.1<br>357.2 → 339.2<br>CBDV **287.2 → 165.1**<br>287.2 → 123.0<br>287.2 → 231.0<br>CBDVA **329.2 → 217.0**<br>329.2 → 283.1<br>329.2 → 311.1<br>CBG **317.2 → 193.0**<br>317.2 → 123.1<br>317.2 → 207.0<br>CBGA **359.2 → 341.1**<br>359.2 → 315.2<br>359.2 → 297.0<br>THCV **287.2 → 165.1**<br>287.2 → 135.1<br>287.2 → 123.0<br>THCVA **329.2 → 285.1**<br>329.2 → 217.0<br>329.2 → 163.1<br>Δ^8-THC **315.3 → 193.1**<br>315.3 → 259.1<br>315.3 → 123.0<br>THC-COOH-d_3<br>**346.2 → 302.2**<br>346.2 → 248.1<br>346.2 → 194.1				
**spiked cannabis-infused chocolate / 0.5 g**	Nexera Thermo trace 1310 UHPLC + Shimadzu 8060 QQQ	MPA: H_2O + 5 mM ammonium formate + 0.1% HCOOH<br>MPB: ACN + 0.1% HCOOH<br>isocratic, (25/75, *v*/*v*)	ESI (+) and (-)<br>MRM mode<br>CBD 315.3 → 193.0<br>315.3 → 123.1<br>CBN 311.3 → 223.3<br>311.3 → 293.3	10.00	-	2020, USA	[139]

Sample type/matrix/ amount (in grams or concentration)	Instrument type and column	LC conditions: mobile phases, MPGP (A%/B%/C%), FR, IV, CT	compounds MS data (quantification ion/MRM), WL	Runtime (min)	LOD/LOQ (ng/mL or ng/g), * - LOD/*LOQ* expressed in % (w/w)	Year, country	Reference
	Raptor ARC-18 (100 mm x 2.1 mm x 2.7 µm) + Raptor ARC-18 EXP guard column (5 mm x 2.1 mm x 2.7 µm)	FR: 0.4 $mLmin^{-1}$<br>IV: 1 µL<br>CT: 30°C	Δ^9-THC 315.3 → 193.0<br>315.3 → 123.1				
	Waters Acquity HPLC + PDA		CBD, CBN, CBG, CBDA, Δ^9-THC, THCA<br>228 nm		-		
**cannabis plant material/ 2.0 g or 5.0 g**	Thermo Fischer UlitMate® 3000 + TSQ Quantum Access Max QQQ<br>Luna Omega Polar C_{18} (100 mm x 2.1 mm x 1.6 µm)	MPA: H_2O + 0.1% HCOOH<br>MPB: ACN + 0.1% HCOOH<br>0 min (40/60), 11 min (20/80), 12.5 min (0/100) + re-equilibration 4.5 min<br>FR: 0.3 $mLmin^{-1}$<br>IV: 2 µL<br>CT: 40°C	MRM mode<br>ESI (+):<br>CBD 315 → 259<br>315 → 193<br>CBN 311 → 293<br>311 → 233<br>Δ^9-THC 315 → 259<br>315 → 193<br>Δ^8-THC 315 → 259<br>315 → 193<br>CBD-d_3 318 → 262<br>318 → 196<br>CBN-d_3 314 → 296<br>314 → 223<br>Δ^9-THC-d_3 318 → 262<br>318 → 196<br>ESI (-):<br>CBDA 357 → 339<br>357 → 311<br>THCA 357 → 339<br>357 → 245	17.00	CBDA 1.9/*5.9*<br>CBD 6.4/*19.3*<br>CBN 6.9/*20.9*<br>Δ9-THC 8.5/*25.8*<br>Δ8-THC 5.6/*17.1*<br>THCA 5.7/*17.3*	2021, Czech Republic	[55]
**fresh cannabis inflorescences / 0.05 g**					-	2021, Czech Republic	[140]

Table S3. Vibrational spectroscopy-based analytical methods in conjunction with multivariate data analysis for phytocannabinoid profiling and/or classification of cannabis plant material. ATR-MIR – attenuated total reflection mid infrared spectroscopy; HCA - hierarchical cluster analysis; OPLS-DA, orthogonal projections to latent structures- discriminatory analysis; PCA – principal component analysis; PLS – partial least square; SEE – standard error of estimation; SVM-DA - support vector machine-discriminatory analysis.

**Sample type**	**Vibrational spectroscopy technique**	**Quantified phytocannabinoids**	**Spectral region (cm^{-1})**	**Statistical model used**	**Number of main model components**	**Statistical accuracy descriptors**					**Referent analytical technique**	**Ref.**
						**Calibration set**			**Prediction set**			
						**R^2**	**SEE (%)**	**SEEcv (%)**	**R^2**	**SEE (%)**		
		CBDV	2500 - 800		12	0.95	0.10	0.15	0.92	0.16		
		Δ⁹-THCV	2500 - 1100		12	0.92	0.02	0.02	0.87	0.03		
		CBD	2500 - 1100		10	0.99	0.35	0.42	0.98	0.58		
		CBC	2500 - 800		9	0.97	0.03	0.04	0.93	0.05		
	NIR (dispersive)	Δ⁸-THC	2500 - 1100		9	0.97	0.03	0.03	0.85	0.07		
		Δ⁹-THC	2500 - 1100		11	0.99	0.58	0.77	0.90	1.72		
**dried cannabis leaves and inflorescences / 30.0 g**		CBG	2500 - 800	PLS	8	0.94	0.25	0.28	0.54	0.79	GC-FID	[141]
		CBN	2500 - 800		7	0.95	0.02	0.03	0.76	0.05		
		CBDV	9403.7-8447.2; 6102-4242.9		11	0.89	0.13	0.17	0.93	0.21		
		Δ⁹-THCV	9403.7-5446.3		10	0.89	0.02	0.03	0.86	0.04		
	FT-NIR	CBD	7506-5446.3; 4428-4242.9		12	0.99	0.29	0.38	0.99	0.62		
		CBC	9403.7-4597.7		10	0.96	0.04	0.05	0.96	0.05		

Sample type	Vibrational spectroscopy technique	Quantified phytocannabinoids	Spectral region (cm^{-1})	Statistical model used	Number of main model components	Statistical accuracy descriptors: Calibration set R^2	Calibration set SEE (%)	Calibration set SEEcv (%)	Prediction set R^2	Prediction set SEE (%)	Referent analytical technique	Ref.
		Δ8-THC	9403.7-7498.3; 6102-4242.9		10	0.98	0.02	0.03	0.91	0.07		
		Δ9-THC	9403.7-5446.3		12	0.99	0.49	0.62	0.95	1.79		
		CBG	7506-6796.3; 4428-4242.9		12	0.96	0.18	0.22	0.78	0.68		
		CBN	6102-5446.3; 4605.4-4242.9		10	0.96	0.02	0.02	0.83	0.06		
**medium-chain triglyceride based formulations, PG-based formulations**	FT NIR	CBD	9020-4000	PLS	4	0.99	0.43	-	0.99	0.37	HPLC-DAD	[142]
			9000-4000		6	0.99	0.30	-	0.99	0.32		
**dried cannabis leaves, stems and inflorescences**	NIR (integrating sphere)	-	4375-4000	PCA HCA PLS-DA SVM-DA	3	-	-	-	-	-	-	[143]
**dried cannabis plant material**	FT-NIR (dispersive)	-	6000-4000	PCA HCA	3	0.97	-	-	-	-	GC-FID	[144]
				SIMCA	3	-	-	-	-	-		

Sample type	Vibrational spectroscopy technique	Quantified phytocannabinoids	Spectral region (cm^{-1})	Statistical model used	Number of main model components	Statistical accuracy descriptors					Referent analytical technique	Ref.
						Calibration set			Prediction set			
						R^2	SEE (%)	SEEcv (%)	R^2	SEE (%)		
	NIR handheld			PCA<br>HCA	3	0.99	-	-	-	-		
				SIMCA	2	-	-	-	-	-		
**veterinary feed spiked with CBD, THC, CBG**	NIR handheld	Δ^9-THC	1700-900	PLS	3	-	0.002	0.006	-	0.009	GC-MS	[145]
		CBD			3	-	0.004	0.004	-	0.005		
		CBG			3	-	0.001	0.002	-	0.005		
**seized cannabis inflorescneces**<br>entire	NIR handheld NIR-S-G1	Δ^9-THC	950-1650	PLS and ensemble regression models	-	0.93	-	-	0.73	-	UHPLC -UV	[146]
ground						0.96	-	-	0.74	-		
sieved						0.98	-	-	0.93	-		
**cannabis resins**						0.72	-	-	0.02	-		
entire	NIR handheld MicroNIR		900-1700		-	0.98	-	-	0.93	-		
ground						0.94	-	-	0.76	-		
sieved						0.98	-	-	0.77	-		
**cannabis resins**						0.87	-	-	0.67	-		
**dried cannabis inflorescences**	ATR-MIR	Δ^9-THCA	4000-400	PLS	-	0.95	-	-	-	0.86	HPLC - DAD	[147]
		Δ^9-THC				0.95	-	-	-	0.13		
		CBDA				0.93	-	-	-	0.80		
		CBD				0.90	-	-	-	0.08		
		CBGA				0.90	-	-	-	0.12		
		CBG				0.78	-	-	-	0.02		
		THCVA				0.70	-	-	-	0.01		
**decarboxylated cannabis flowers**	ATR-MIR	Δ^9-THC	1800-400	PLS	5	0.99	0.43	1.53	-	2.32	HPLC-UV	[148]
					5	0.99	0.21	1.41	-	1.31		
**cannabis extracts**		CBD	1800-400	PLS	3	0.95	4.67	5.25	-	3.79		
					3	0.99	1.21	2.62	-	1.44		
**cannabis extracts**	handheld Raman spectrometer	-	1700-701	OPLS-DA	1+2	-	-	-	-	-	CoA from the plant	[149,150]

Sample type	Vibrational spectroscopy technique	Quantified phytocannabinoids	Spectral region (cm^{-1})	Statistical model used	Number of main model components	Statistical accuracy descriptors					Referent analytical technique	Ref.
						Calibration set			Prediction set			
						R^2	SEE (%)	SEEcv (%)	R^2	SEE (%)		
											producer	

References

1. E.C. Union Method for the Quantitative Determination of the Δ9-Tetrahydrocannabinol Content in Hemp Varieties. Delegated Regulation (EU) No 639/2014, Annex III as Amended by Regulation (EU) 2017/1155. *Off. J. Eur. Union* **2017**, *167*, 1–15.
2. Sgrò, S. Delta9-THC Determination by the EU Official Method: Evaluation of Measurement Uncertainty and Compliance Assessment of Hemp Samples. 12.
3. Potter, D.J.; Hammond, K.; Tuffnell, S.; Walker, C.; Di Forti, M. Potency of Δ9-Tetrahydrocannabinol and Other Cannabinoids in Cannabis in England in 2016: Implications for Public Health and Pharmacology. *Drug Test. Anal.* **2018**, *10*, 628–635, doi:10.1002/dta.2368.
4. Pijlman, F.; Rigter, S.; Hoek, J.; Goldschmidt, H.; Niesink, R. Strong Increase in Total Delta-THC in Cannabis Preparations Sold in Dutch Coffee Shops. *Addict. Biol.* **2005**, *10*, 171–180, doi:10.1080/13556210500123217.
5. van der Pol, P.; Liebregts, N.; de Graaf, R.; Korf, D.J.; van den Brink, W.; van Laar, M. Validation of Self-Reported Cannabis Dose and Potency: An Ecological Study: Self-Reported Cannabis Dose and Potency. *Addiction* **2013**, *108*, 1801–1808, doi:10.1111/add.12226.
6. van der Pol, P.; Liebregts, N.; Brunt, T.; van Amsterdam, J.; de Graaf, R.; Korf, D.J.; van den Brink, W.; van Laar, M. Cross-Sectional and Prospective Relation of Cannabis Potency, Dosing and Smoking Behaviour with Cannabis Dependence: An Ecological Study: Cannabis Potency, Titration and Dependence. *Addiction* **2014**, *109*, 1101–1109, doi:10.1111/add.12508.
7. Niesink, R.J.M.; Rigter, S.; Koeter, M.W.; Brunt, T.M. Potency Trends of Δ9-Tetrahydrocannabinol, Cannabidiol and Cannabinol in Cannabis in the Netherlands: 2005-15: Potency Trends of Dutch Cannabis. *Addiction* **2015**, *110*, 1941–1950, doi:10.1111/add.13082.
8. Zamengo, L.; Frison, G.; Bettin, C.; Sciarrone, R. Variability of Cannabis Potency in the Venice Area (Italy): A Survey over the Period 2010-2012: Variability of Cannabis Potency in the Venice Area (Italy): A Survey over the Period 2010-2012. *Drug Test. Anal.* **2014**, *6*, 46–51, doi:10.1002/dta.1515.
9. Zamengo, L.; Frison, G.; Bettin, C.; Sciarrone, R. Cannabis Potency in the Venice Area (Italy): Update 2013: Cannabis Potency in the Venice Area (Italy): Update 2013. *Drug Test. Anal.* **2015**, *7*, 255–258, doi:10.1002/dta.1690.
10. Hillig, K.W.; Mahlberg, P.G. A Chemotaxonomic Analysis of Cannabinoid Variation in Cannabis (Cannabaceae). *Am. J. Bot.* **2004**, *91*, 966–975, doi:10.3732/ajb.91.6.966.
11. Poulsen, H.A.; Sutherland, G.J. The Potency of Cannabis in New Zealand from 1976 to 1996. *Sci. Justice* **2000**, *40*, 171–176, doi:10.1016/S1355-0306(00)71972-1.
12. P. S. Fetterman, N. J. Doorenbos, A Simple Gas Liquid Chromatography Procedure for Determination of Cannabinoldlc Acids In Cannabis Sativa L. *Experimentia* **1971**, *27*, 988–990.
13. Baker, P.B.; Taylor, B.J.; Gough, T.A. The Tetrahydrocannabinol and Tetrahydrocannabinolic Acid Content of Cannabis Products. *J. Pharm. Pharmacol.* **1981**, *33*, 369–372, doi:10.1111/j.2042-7158.1981.tb13806.x.
14. Pitts, J.E.; O'Neil, P.J.; Leggo, K.P. Variation in the THC Content of Illicitly Imported Cannabis* Products-1984-1989. *J. Pharm. Pharmacol.* **1990**, *42*, 817–820, doi:10.1111/j.2042-7158.1990.tb07032.x.
15. Kaa, E. Cannabis Plants Illicitly Grown in Jutland (Denmark). *Z Rechtsmed* **1989**, *102*, 367–375, doi:https://doi.org/10.1007/BF00200245.
16. Dussy, F.E.; Hamberg, C.; Luginbühl, M.; Schwerzmann, T.; Briellmann, T.A. Isolation of Δ9-THCA-A from Hemp and Analytical Aspects Concerning the Determination of Δ9-THC in Cannabis Products. *Forensic Sci. Int.* **2005**, *149*, 3–10, doi:10.1016/j.forsciint.2004.05.015.
17. Stefanidou, M.; Dona, A.; Athanaselis, S.; Papoutsis, I.; Koutselinis, A. The Cannabinoid Content of Marihuana Samples Seized in Greece and Its Forensic Application. *Forensic Sci. Int.* **1998**, *95*, 153–162, doi:10.1016/S0379-0738(98)00083-8.
18. Stefanidou, M.; Athanaselis, S.; Alevisopolous, G.; Papoutsis, I.; Koutselinis, A. Delta9-Tetrahydrocannabinol Content in Cannabis Plants of Greek Origin. *Chem. Pharm. Bull. (Tokyo)* **2000**, *48*, 743–745, doi:oi: 10.1248/cpb.48.743.
19. Pacifico, D.; Miselli, F.; Micheler, M.; Carboni, A.; Ranalli, P.; Mandolino, G. Genetics and Marker-Assisted Selection of the Chemotype in Cannabis Sativa L. *Mol. Breed.* **2006**, *17*, 257–268, doi:10.1007/s11032-005-5681-x.

20. Pacifico, D.; Miselli, F.; Carboni, A.; Moschella, A.; Mandolino, G. Time Course of Cannabinoid Accumulation and Chemotype Development during the Growth of Cannabis Sativa L. *Euphytica* **2008**, *160*, 231–240, doi:10.1007/s10681-007-9543-y.
21. Tipparat, P.; Natakankitkul, S.; Chamnivikaipong, P.; Chutiwat, S. Characteristics of Cannabinoids Composition of Cannabis Plants Grown in Northern Thailand and Its Forensic Application. *Forensic Sci. Int.* **2012**, *215*, 164–170, doi:10.1016/j.forsciint.2011.05.006.
22. Tipparat, P.; Kunkaew, W.; Julsrigival, S.; Pinmanee, S.; Natakankitkul, S. Classification of Cannabis Plants Grown in Northern Thailand Using Physico-Chemical Properties. **2014**, 10.
23. Hazekamp, A.; Fischedick, J.T. Cannabis - from Cultivar to Chemovar: Towards a Better Definition of Cannabis Potency. *Drug Test. Anal.* **2012**, *4*, 660–667, doi:10.1002/dta.407.
24. Hazekamp, A.; Tejkalová, K.; Papadimitriou, S. Cannabis: From Cultivar to Chemovar II—A Metabolomics Approach to Cannabis Classification. *Cannabis Cannabinoid Res.* **2016**, *1*, 202–215, doi:10.1089/can.2016.0017.
25. Fischedick, J.T.; Hazekamp, A.; Erkelens, T.; Choi, Y.H.; Verpoorte, R. Metabolic Fingerprinting of Cannabis Sativa L., Cannabinoids and Terpenoids for Chemotaxonomic and Drug Standardization Purposes. *Phytochemistry* **2010**, *71*, 2058–2073, doi:10.1016/j.phytochem.2010.10.001.
26. Vanhove, W.; Van Damme, P.; Meert, N. Factors Determining Yield and Quality of Illicit Indoor Cannabis (Cannabis Spp.) Production. *Forensic Sci. Int.* **2011**, *212*, 158–163, doi:10.1016/j.forsciint.2011.06.006.
27. Stambouli, H.; El Bouri, A.; Bouayoun, T. Évolution de la teneur en Δ9-THC dans les saisies de résines de cannabis au Maroc de 2005 à 2014. *Toxicol. Anal. Clin.* **2016**, *28*, 146–152, doi:10.1016/j.toxac.2015.11.001.
28. Field, B.I.; Arndt, R.R. Cannabinoid Compounds in South African Cannabis Sativa L. *J. Pharm. Pharmacol.* **1980**, *32*, 21–24, doi:10.1111/j.2042-7158.1980.tb12838.x.
29. Tucker, R.B.; Graham, B.F. Cannabinoid Content of Colombian Cannabis. *Can. Soc. Forensic Sci. J.* **1981**, *14*, 41–45, doi:10.1080/00085030.1981.10756878.
30. de Oliveira, G.L.; Voloch, M.H.; Sztulman, G.B.; Neto, O.N.; Yonamine, M. Cannabinoid Contents in Cannabis Products Seized in São Paulo, Brazil, 2006–2007. *Forensic Toxicol.* **2008**, *26*, 31–35, doi:10.1007/s11419-008-0046-x.
31. Gambaro, V.; Dell'Acqua, L.; Farè, F.; Froldi, R.; Saligari, E.; Tassoni, G. Determination of Primary Active Constituents in Cannabis Preparations by High-Resolution Gas Chromatography/FLame Ionization Detection and High-Performance Liquid Chromatography/UV Detection. *Anal. Chim. Acta* **2002**, *468*, 245–254.
32. Ibrahim, E.; Gul, W.; Gul, S.; Stamper, B.; Hadad, G.; Abdel Salam, R.; Ibrahim, A.; Ahmed, S.; Chandra, S.; Lata, H.; et al. Determination of Acid and Neutral Cannabinoids in Extracts of Different Strains of Cannabis Sativa Using GC-FID. *Planta Med.* **2018**, *84*, 250–259, doi:10.1055/s-0043-124088.
33. Janatová, A.; Fraňková, A.; Tlustoš, P.; Hamouz, K.; Božik, M.; Klouček, P. Yield and Cannabinoids Contents in Different Cannabis (Cannabis Sativa L.) Genotypes for Medical Use. *Ind. Crops Prod.* **2018**, *112*, 363–367, doi:10.1016/j.indcrop.2017.12.006.
34. *UNODC - Bulletin on Narcotics - 1971 Issue 1 - 005*; United Nations, Office on Drugs and Crime: Vienna, AU, 1971;
35. Fairbairn, J.W.; Liebmann, J.A. The Extraction and Estimation of the Cannabinoids in Cannabis Sativa L. and Its Products. *J. Pharm. Pharmacol.* **1973**, *25*, 150–155, doi:10.1111/j.2042-7158.1973.tb10609.x.
36. United Nations Office on Drugs and Crime Recommended Methods for the Identification and Analysis of Cannabis and Cannabis Products Manual for Use by National Drug Analysis Laboratories 2009.
37. de Meijer, E.P.M.; Bagatta, M.; Carboni, A.; Crucitiit, P.; Moliterni, C.V.M.; Ranalli, P.; Mandolino, G. The Inheritance of Chemical Phenotype in Cannabis Sativa L. *Genetics* **2003**, *163*, 335–346.
38. Florian-Ramirez, N.M.; Garzon-Mendez, W.F.; Parada-Alfonso, F. Gas Chromatography in Forensic Chemistry: Cannabinoids Content in Marijuana Leaves (Cannabis Sativa L.) from Colombia. In *Gas Chromatography - Biochemicals, Narcotics and Essential Oils*; Salih, B., Ed.; InTech, 2012 ISBN 978-953-51-0295-3.
39. Potter, D.J.; Clark, P.; Brown, M.B. Potency of Δ9–THC and Other Cannabinoids in Cannabis in England in 2005: Implications for Psychoactivity and Pharmacology*. *J. Forensic Sci.* **2008**, *53*, 90–94, doi:10.1111/j.1556-4029.2007.00603.x.
40. Turner, J.C.; Hemphill, J.K.; Mahlberg, P.G. Gland Distribution and Cannabinoid Content in Clones of Cannabis Sativa L. *Am. J. Bot.* **1977**, *64*, 687–693, doi:10.1002/j.1537-2197.1977.tb11910.x.

41. Mehmedic, Z.; Chandra, S.; Slade, D.; Denham, H.; Foster, S.; Patel, A.S.; Ross, S.A.; Khan, I.A.; ElSohly, M.A. Potency Trends of Δ9-THC and Other Cannabinoids in Confiscated Cannabis Preparations from 1993 to 2008*. *J. Forensic Sci.* **2010**, *55*, 1209–1217, doi:10.1111/j.1556-4029.2010.01441.x.
42. ElSohly, M.A.; Mehmedic, Z.; Foster, S.; Gon, C.; Chandra, S.; Church, J.C. Changes in Cannabis Potency Over the Last 2 Decades (1995–2014): Analysis of Current Data in the United States. *Biol. Psychiatry* **2016**, *79*, 613–619, doi:10.1016/j.biopsych.2016.01.004.
43. Chandra, S.; Radwan, M.M.; Majumdar, C.G.; Church, J.C.; Freeman, T.P.; ElSohly, M.A. New Trends in Cannabis Potency in USA and Europe during the Last Decade (2008–2017). *Eur. Arch. Psychiatry Clin. Neurosci.* **2019**, *269*, 5–15, doi:10.1007/s00406-019-00983-5.
44. Chandra, S.; Lata, H.; Mehmedic, Z.; Khan, I.; ElSohly, M. Assessment of Cannabinoids Content in Micropropagated Plants of Cannabis Sativa and Their Comparison with Conventionally Propagated Plants and Mother Plant during Developmental Stages of Growth. *Planta Med.* **2010**, *76*, 743–750, doi:10.1055/s-0029-1240628.
45. Harvey, D.J. Cyclic Alkylboronates as Derivatives for the Characterization of Cannabinolic Acids by Combined Gas Chromatography and Mass Spectrometry. *Biol. Mass Spectrom.* **1977**, *4*, 88–93, doi:10.1016/j.jpba.2017.11.073.
46. Gröger, Th.; Schäffer, M.; Pütz, M.; Ahrens, B.; Drew, K.; Eschner, M.; Zimmermann, R. Application of Two-Dimensional Gas Chromatography Combined with Pixel-Based Chemometric Processing for the Chemical Profiling of Illicit Drug Samples. *J. Chromatogr. A* **2008**, *1200*, 8–16, doi:10.1016/j.chroma.2008.05.028.
47. Mölleken, H.; Husmann, H. Cannabinoids in Seed Extracts of Cannabis Sativa Cultivars. *J. Int. Hemp Assoc.* **1997**, *4*, 76–79.
48. Florian-Ramirez, N.M.; Parada-Alfonso, F.; Garzon-Mendez, W.F. Estudio del contenido de cannabinoids en muestras de marihuana (Cannabis sativa L.) cultivadas en varies regiones de Colombia. *Vitae Rev. Faculdad Quimica Farm.* **2009**, *16*, 237–244.
49. Casiraghi, A.; Roda, G.; Casagni, E.; Cristina, C.; Musazzi, U.; Franzè, S.; Rocco, P.; Giuliani, C.; Fico, G.; Minghetti, P.; et al. Extraction Method and Analysis of Cannabinoids in Cannabis Olive Oil Preparations. *Planta Med.* **2018**, *84*, 242–249, doi:10.1055/s-0043-123074.
50. Trofin, I.G.; Vlad, C.C.; Noja, V.V.; Dabija, G. Identification and Characterization of Special Types of Herbal Cannabis. *U.P.B.Sciu.Bull.* **2012**, *74*, 13.
51. de Meijer, E.P.M.; Hammond, K.M.; Sutton, A. The Inheritance of Chemical Phenotype in Cannabis Sativa L. (IV): Cannabinoid-Free Plants. *Euphytica* **2009**, *168*, 95–112, doi:10.1007/s10681-009-9894-7.
52. Tsumura, Y.; Aoki, R.; Tokieda, Y.; Akutsu, M.; Kawase, Y.; Kataoka, T.; Takagi, T.; Mizuno, T.; Fukada, M.; Fujii, H.; et al. A Survey of the Potency of Japanese Illicit Cannabis in Fiscal Year 2010. *Forensic Sci. Int.* **2012**, *221*, 77–83, doi:10.1016/j.forsciint.2012.04.005.
53. Omar, J.; Olivares, M.; Amigo, J.M.; Etxebarria, N. Resolution of Co-Eluting Compounds of Cannabis Sativa in Comprehensive Two-Dimensional Gas Chromatography/Mass Spectrometry Detection with Multivariate Curve Resolution-Alternating Least Squares. *Talanta* **2014**, *121*, 273–280, doi:10.1016/j.talanta.2013.12.044.
54. Micalizzi, G.; Alibrando, F.; Vento, F.; Trovato, E.; Zoccali, M.; Guarnaccia, P.; Dugo, P.; Mondello, L. Development of a Novel Microwave Distillation Technique for the Isolation of Cannabis Sativa L. Essential Oil and Gas Chromatography Analyses for the Comprehensive Characterization of Terpenes and Terpenoids, Including Their Enantio-Distribution. *Molecules* **2021**, *26*, 1588, doi:10.3390/molecules26061588.
55. Béres, T.; Černochová, L.; Ćavar Zeljković, S.; Benická, S.; Gucký, T.; Berčák, M.; Tarkowski, P. Intralaboratory Comparison of Analytical Methods for Quantification of Major Phytocannabinoids. *Anal. Bioanal. Chem.* **2019**, *411*, 3069–3079, doi:10.1007/s00216-019-01760-y.
56. Licata, M.; Verri, P.; Beduschi, G. Δ9 THC Content in Illicit Cannabis Products. *Ann Ist Super Sanita* **2005**, *41*, 483–485.
57. Pellegrini, M.; Marchei, E.; Pacifici, R.; Pichini, S. A Rapid and Simple Procedure for the Determination of Cannabinoids in Hemp Food Products by Gas Chromatography-Mass Spectrometry. *J. Pharm. Biomed. Anal.* **2005**, *36*, 939–946, doi:10.1016/j.jpba.2004.07.035.
58. Lachenmeier, D.W.; Kroener, L.; Musshoff, F.; Madea, B. Determination of Cannabinoids in Hemp Food Products by Use of Headspace Solid-Phase Microextraction and Gas Chromatography-Mass Spectrometry. *Anal. Bioanal. Chem.* **2004**, *378*, 183–189, doi:10.1007/s00216-003-2268-4.

59. Mariotti, K. de C.; Marcelo, M.C.A.; Ortiz, R.S.; Borille, B.T.; dos Reis, M.; Fett, M.S.; Ferrão, M.F.; Limberger, R.P. Seized Cannabis Seeds Cultivated in Greenhouse: A Chemical Study by Gas Chromatography–Mass Spectrometry and Chemometric Analysis. *Sci. Justice* **2016**, *56*, 35–41, doi:10.1016/j.scijus.2015.09.002.
60. Ciolino, L.A.; Ranieri, T.L.; Taylor, A.M. Commercial Cannabis Consumer Products Part 1: GC–MS Qualitative Analysis of Cannabis Cannabinoids. *Forensic Sci. Int.* **2018**, *289*, 429–437, doi:10.1016/j.forsciint.2018.05.032.
61. Omar, J.; Olivares, M.; Alzaga, M.; Etxebarria, N. Optimisation and Characterisation of Marihuana Extracts Obtained by Supercritical Fluid Extraction and Focused Ultrasound Extraction and Retention Time Locking GC-MS: Gas Chromatography. *J. Sep. Sci.* **2013**, *36*, 1397–1404, doi:10.1002/jssc.201201103.
62. Knight, G.; Hansen, S.; Connor, M.; Poulsen, H.; McGovern, C.; Stacey, J. The Results of an Experimental Indoor Hydroponic Cannabis Growing Study, Using the 'Screen of Green' (ScrOG) Method—Yield, Tetrahydrocannabinol (THC) and DNA Analysis. *Forensic Sci. Int.* **2010**, *202*, 36–44, doi:10.1016/j.forsciint.2010.04.022.
63. Trigg, S. Development of Gas and Liquid Chromatographic Methods for the Separation and Quantification of 11 Cannabinoids. Bachelor Thesis, School of Veterinary and Life Sciences, of Murdoch University, 2017.
64. Broséus, J.; Anglada, F.; Esseiva, P. The Differentiation of Fibre- and Drug Type Cannabis Seedlings by Gas Chromatography/Mass Spectrometry and Chemometric Tools. *Forensic Sci. Int.* **2010**, *200*, 87–92, doi:10.1016/j.forsciint.2010.03.034.
65. Namdar, D.; Mazuz, M.; Ion, A.; Koltai, H. Variation in the Compositions of Cannabinoid and Terpenoids in Cannabis Sativa Derived from Inflorescence Position along the Stem and Extraction Methods. *Ind. Crops Prod.* **2018**, *113*, 376–382, doi:10.1016/j.indcrop.2018.01.060.
66. Kladar, N.; Čonić, B.S.; Božin, B.; Torović, L. European Hemp-Based Food Products – Health Concerning Cannabinoids Exposure Assessment. *Food Control* **2021**, *129*, 108233, doi:10.1016/j.foodcont.2021.108233.
67. Cadola, L.; Broséus, J.; Esseiva, P. Chemical Profiling of Different Hashish Seizures by Gas Chromatography–Mass Spectrometry and Statistical Methodology: A Case Report. *Forensic Sci. Int.* **2013**, *232*, e24–e27, doi:10.1016/j.forsciint.2013.08.014.
68. Jang, E.; Kim, H.; Jang, S.; Lee, J.; Baeck, S.; In, S.; Kim, E.; Kim, Y.; Han, E. Concentrations of THC, CBD, and CBN in Commercial Hemp Seeds and Hempseed Oil Sold in Korea | Elsevier Enhanced Reader. *Forensic Sci. Int.* **2020**, *306*, 110064, doi:10.1016/j.forsciint.2019.110064.
69. dos Santos, N.; Tose, L.; da Silva, S.; Murgu, M.; Kuster, R.; Ortiz, R.; Camargo, F.; Vaz, B.; Lacerda Jr., V.; Romão, W. Analysis of Isomeric Cannabinoid Standards and Cannabis Products by UPLC-ESI-TWIM-MS: A Comparison with GC-MS and GC × GC-QMS. *J. Braz. Chem. Soc.* **2019**, *30*, 60–70, doi:10.21577/0103-5053.20180152.
70. Bruci, Z.; Papoutsis, I.; Athanaselis, S.; Nikolaou, P.; Pazari, E.; Spiliopoulou, C.; Vyshka, G. First Systematic Evaluation of the Potency of Cannabis Sativa Plants Grown in Albania. *Forensic Sci. Int.* **2012**, *222*, 40–46, doi:10.1016/j.forsciint.2012.04.032.
71. Qureshi, M.N.; Kanwal, F.; Siddique, M.; Akram, M. Estimation of Biologically Active Cannabinoids in Cannabis Indica by Gas Chromatography-Mass Spectrometry (GC-MS). **2012**, 7.
72. Isahq, M.S.; Afridi, M.S.; Ali, J.; Hussain, M.M.; Ahmad, S.; Kanwal, F. Proximate Composition, Phytochemical Screening, GC-MS Studies of Biologically Active Cannabinoids and Antimicrobial Activities of Cannabis Indica. *Asian Pac. J. Trop. Dis.* **2015**, *5*, 897–902, doi:10.1016/S2222-1808(15)60953-7.
73. Cardenia, V.; Gallina Toschi, T.; Scappini, S.; Rubino, R.C.; Rodriguez-Estrada, M.T. Development and Validation of a Fast Gas Chromatography/Mass Spectrometry Method for the Determination of Cannabinoids in Cannabis Sativa L. *J. Food Drug Anal.* **2018**, *26*, 1283–1292, doi:10.1016/j.jfda.2018.06.001.
74. Ross, S.A.; Mehmedic, Z.; Murphy, T.P.; ElSohly, M.A. GC-MS Analysis of the Total Delta9-THC Content of Both Drug- and Fiber-Type Cannabis Seeds. *J. Anal. Toxicol.* **2000**, *24*, 715–717, doi:https://doi.org/10.1093/jat/24.8.715.
75. Caligiani, A.; Palla, G.; Bernardelli, B. GC-MS Analysis of Hashish Samples: A Case of Adulteration with Colophony. *J. Forensic Sci.* **2006**, *51*, 1096–1100, doi:10.1111/j.1556-4029.2006.00202.x.
76. Ilias, Y.; Rudaz, S.; Mathieu, P.; Veuthey, J.-L.; Christen, P. Analysis of Cannabis Material by Headspace Solid-Phase Microextraction Combined with Gas Chromatography-Mass Spectrometry. *Chim. Int. J. Chem.* **2004**, *58*, 219–221, doi:10.2533/000942904777677957.
77. Mechtler, K.; Bailer, J.; de Hueber, K. Variations of Δ9-THC Content in Single Plants of Hemp Varieties. 6.

78. Lewis, R.; Ward, S.; Johnson, R.; Burns, D.T. Distribution of the Principal Cannabinoids within Bars of Compressed Cannabis Resin. *Anal. Chim. Acta* **2005**, *538*, 399–405, doi:10.1016/j.aca.2005.02.014.
79. Ilias, Y.; Rudaz, S.; Mathieu, P.; Christen, P.; Veuthey, J.-L. Extraction and Analysis of Different Cannabis Samples by Headspace Solid-Phase Microextraction Combined with Gas Chromatography-Mass Spectrometry. *J. Sep. Sci.* **2005**, *28*, 2293–2300, doi:10.1002/jssc.200500130.
80. Petrović, M.; Debeljak, Ž.; Kezić, N.; Džidara, P. Relationship between Cannabinoids Content and Composition of Fatty Acids in Hempseed Oils. *Food Chem.* **2015**, *170*, 218–225, doi:10.1016/j.foodchem.2014.08.039.
81. Amirav, A.; Neumark, B.; Margolin Eren, K.J.; Fialkov, A.B.; Tal, N. Cannabis and Its Cannabinoids Analysis by Gas Chromatography–Mass Spectrometry with Cold EI. *J. Mass Spectrom.* **2021**, *56*, doi:10.1002/jms.4726.
82. Yotoriyama, M.; Ishiharajima, E.; Kato, Y.; Nagato, A.; Sekita, S.; Watanabe, K.; Yamamoto, I. Identification and Determination of Cannabinoids in Both Commercially Available and Cannabis Oils Stored Long Term. *J. Health Sci.* **2005**, *51*, 483–487, doi:10.1248/jhs.51.483.
83. Hewavitharana, A.K.; Golding, G.; Tempany, G.; King, G.; Holling, N. Quantitative GC-MS Analysis of Δ9-Tetrahydrocannabinol in Fiber Hemp Varieties. *J. Anal. Toxicol.* **2005**, *29*, 258–261, doi:10.1093/jat/29.4.258.
84. Hazekamp, A.; Peltenburg, A.; Verpoorte, R.; Giroud, C. Chromatographic and Spectroscopic Data of Cannabinoids from Cannabis Sativa L. *J. Liq. Chromatogr. Relat. Technol.* **2005**, *28*, 2361–2382, doi:10.1080/10826070500187558.
85. Stambouli, H.; Bouri, A.E.; Bellimam, M.A.; Bouayoun, T.; Karni, N.E. Cultivation of Cannabis Sativa L. in Northern Morocco. *Bull. Narc.* **2005**, 41.
86. Fodor, B.; Boldizsár, I.; Molnár-Perl, I. Alkylsilyl Speciation and Direct Sample Preparation of Plant Cannabinoids Prior to Their Analysis by GC-MS. *Anal. Chim. Acta* **2018**, *1021*, 51–59, doi:10.1016/j.aca.2018.03.049.
87. Leghissa, A. Method Development for Qualification and Quantificatin of Cannabinoids and Terpenes in Extracts by Gas Chromatography-Mass Spectrometry. 91.
88. Delgado-Povedano, M.M.; Sánchez-Carnerero Callado, C.; Priego-Capote, F.; Ferreiro-Vera, C. Untargeted Characterization of Extracts from Cannabis Sativa L. Cultivars by Gas and Liquid Chromatography Coupled to Mass Spectrometry in High Resolution Mode. *Talanta* **2020**, *208*, 120384, doi:10.1016/j.talanta.2019.120384.
89. Leghissa, A.; Smuts, J.; Qiu, C.; Hildenbrand, Z.L.; Schug, K.A. Detection of Cannabinoids and Cannabinoid Metabolites Using Gas Chromatography with Vacuum Ultraviolet Spectroscopy. *Sep. Sci. Plus* **2018**, *1*, 37–42, doi:10.1002/sscp.201700005.
90. Wianowska, D.; Dawidowicz, A.L.; Kowalczyk, M. Transformations of Tetrahydrocannabinol, Tetrahydrocannabinolic Acid and Cannabinol during Their Extraction from Cannabis Sativa L. *J. Anal. Chem.* **2015**, *70*, 920–925, doi:10.1134/S1061934815080183.
91. Tayyab, M.; Shahwar, D. GCMS Analysis of Cannabis Sativa L. from Four Different Areas of Pakistan. *Egypt. J. Forensic Sci.* **2015**, *5*, 114–125, doi:10.1016/j.ejfs.2014.07.008.
92. Hazekamp, A.; Simons, R.; Peltenburg-Looman, A.; Sengers, M.; van Zweden, R.; Verpoorte, R. Preparative Isolation of Cannabinoids from Cannabis Sativa by Centrifugal Partition Chromatography. *J. Liq. Chromatogr. Relat. Technol.* **2004**, *27*, 2421–2439, doi:10.1081/JLC-200028170.
93. De Backer, B.; Debrus, B.; Lebrun, P.; Theunis, L.; Dubois, N.; Decock, L.; Verstraete, A.; Hubert, P.; Charlier, C. Innovative Development and Validation of an HPLC/DAD Method for the Qualitative and Quantitative Determination of Major Cannabinoids in Cannabis Plant Material. *J. Chromatogr. B* **2009**, *877*, 4115–4124, doi:10.1016/j.jchromb.2009.11.004.
94. Swift, W.; Wong, A.; Li, K.M.; Arnold, J.C.; McGregor, I.S. Analysis of Cannabis Seizures in NSW, Australia: Cannabis Potency and Cannabinoid Profile. *PLoS ONE* **2013**, *8*, e70052, doi:10.1371/journal.pone.0070052.
95. Geschäftsstelle der Arzneibuch-Kommissionen, Bundesinstitut fur Arzneimittel und Miedizinprodukte Monografie Cannabisblüten. In *German Pharmacopoeia*; Geschäftsstelle der Arzneibuch-Kommissionen, Bundesinstitut fur Arzneimittel und Miedizinprodukte: Bonn, 2020 ISBN 978-7692-7553-7.
96. Commission Suisse de Pharmacopée Swissmedic Swiss Agency for Therapeutic Products Pharmacopoeia Division Fleur de Cannabis. In *Pharmacopoeia Helvetica, Supplement 11.3*; Commission Suisse de Pharmacopée Swissmedic Swiss Agency for Therapeutic Products Pharmacopoeia Division: Bernn, 2019.
97. Qamar, S.; Manrique, Y.J.; Parekh, H.S.; Falconer, J.R. Development and Optimization of Supercritical Fluid Extraction Setup Leading to Quantification of 11 Cannabinoids Derived from Medicinal Cannabis. *Biology* **2021**, *10*, 481, doi:10.3390/biology10060481.

98. Peschel, W.; Politi, M. ^{1}H NMR and HPLC/DAD for Cannabis Sativa L. Chemotype Distinction, Extract Profiling and Specification. *Talanta* **2015**, *140*, 150–165, doi:10.1016/j.talanta.2015.02.040.
99. Meng, Q.; Buchanan, B.; Zuccolo, J.; Poulin, M.-M.; Gabriele, J.; Baranowski, D.C. A Reliable and Validated LC-MS/MS Method for the Simultaneous Quantification of 4 Cannabinoids in 40 Consumer Products. *PLOS ONE* **2018**, *13*, e0196396, doi:10.1371/journal.pone.0196396.
100. Berman, P.; Futoran, K.; Lewitus, G.M.; Mukha, D.; Benami, M.; Shlomi, T.; Meiri, D. A New ESI-LC/MS Approach for Comprehensive Metabolic Profiling of Phytocannabinoids in Cannabis. *Sci. Rep.* **2018**, *8*, 14280, doi:10.1038/s41598-018-32651-4.
101. Uziel, A.; Gelfand, A.; Amsalem, K.; Berman, P.; Lewitus, G.M.; Meiri, D.; Lewitus, D.Y. Full-Spectrum Cannabis Extract Microdepots Support Controlled Release of Multiple Phytocannabinoids for Extended Therapeutic Effect. *ACS Appl. Mater. Interfaces* **2020**, *12*, 23707–23716, doi:10.1021/acsami.0c04435.
102. Burnier, C.; Esseiva, P.; Roussel, C. Quantification of THC in Cannabis Plants by Fast-HPLC-DAD: A Promising Method for Routine Analyses. *Talanta* **2019**, *192*, 135–141, doi:10.1016/j.talanta.2018.09.012.
103. Mandrioli, M.; Tura, M.; Scotti, S.; Gallina Toschi, T. Fast Detection of 10 Cannabinoids by RP-HPLC-UV Method in Cannabis Sativa L. *Molecules* **2019**, *24*, 2113, doi:10.3390/molecules24112113.
104. Patel, B.; Wene, D.; Fan, Z. (Tina) Qualitative and Quantitative Measurement of Cannabinoids in Cannabis Using Modified HPLC/DAD Method. *J. Pharm. Biomed. Anal.* **2017**, *146*, 15–23, doi:10.1016/j.jpba.2017.07.021.
105. Pellati, F.; Brighenti, V.; Sperlea, J.; Marchetti, L.; Bertelli, D.; Benvenuti, S. New Methods for the Comprehensive Analysis of Bioactive Compounds in Cannabis Sativa L. (Hemp). *Molecules* **2018**, *23*, 2639, doi:https://doi.org/10.3390/molecules23102639.
106. Ciolino, L.A.; Ranieri, T.L.; Taylor, A.M. Commercial Cannabis Consumer Products Part 2: HPLC-DAD Quantitative Analysis of Cannabis Cannabinoids. *Forensic Sci. Int.* **2018**, *289*, 438–447, doi:10.1016/j.forsciint.2018.05.033.
107. Muscarà, C.; Smeriglio, A.; Trombetta, D.; Mandalari, G.; La Camera, E.; Grassi, G.; Circosta, C. Phytochemical Characterization and Biological Properties of Two Standardized Extracts from a Non-psychotropic *CANNABIS SATIVA* L. Cannabidiol (CBD)-chemotype. *Phytother. Res.* **2021**, ptr.7201, doi:10.1002/ptr.7201.
108. Dubrow, G.A.; Pawar, R.S.; Srigley, C.; Fong Sam, J.; Talavera, C.; Parker, C.H.; Noonan, G.O. A Survey of Cannabinoids and Toxic Elements in Hemp-Derived Products from the United States Marketplace. *J. Food Compos. Anal.* **2021**, *97*, 103800, doi:10.1016/j.jfca.2020.103800.
109. Peace, M.R.; Butler, K.E.; Wolf, C.E.; Poklis, J.L.; Poklis, A. Evaluation of Two Commercially Available Cannabidiol Formulations for Use in Electronic Cigarettes. *Front. Pharmacol.* **2016**, *7*, doi:10.3389/fphar.2016.00279.
110. Aizpurua-Olaizola, O.; Omar, J.; Navarro, P.; Olivares, M.; Etxebarria, N.; Usobiaga, A. Identification and Quantification of Cannabinoids in Cannabis Sativa L. Plants by High Performance Liquid Chromatography-Mass Spectrometry. *Anal. Bioanal. Chem.* **2014**, *406*, 7549–7560, doi:10.1007/s00216-014-8177-x.
111. Wang, M.; Wang, Y.-H.; Avula, B.; Radwan, M.M.; Wanas, A.S.; Mehmedic, Z.; Antwerp, J. van; ElSohly, M.A.; Khan, I.A. Quantitative Determination of Cannabinoids in Cannabis and Cannabis Products Using Ultra-High-Performance Supercritical Fluid Chromatography and Diode Array/Mass Spectrometric Detection. *J. Forensic Sci.* **2017**, *62*, 602–611, doi:10.1111/1556-4029.13341.
112. Citti, C.; Pacchetti, B.; Vandelli, M.A.; Forni, F.; Cannazza, G. Analysis of Cannabinoids in Commercial Hemp Seed Oil and Decarboxylation Kinetics Studies of Cannabidiolic Acid (CBDA). *J. Pharm. Biomed. Anal.* **2018**, *149*, 532–540, doi:10.1016/j.jpba.2017.11.044.
113. Brighenti, V.; Pellati, F.; Steinbach, M.; Maran, D.; Benvenuti, S. Development of a New Extraction Technique and HPLC Method for the Analysis of Non-Psychoactive Cannabinoids in Fibre-Type Cannabis Sativa L. (Hemp). *J. Pharm. Biomed. Anal.* **2017**, *143*, 228–236, doi:10.1016/j.jpba.2017.05.049.
114. Brighenti, V.; Licata, M.; Pedrazzi, T.; Maran, D.; Bertelli, D.; Pellati, F.; Benvenuti, S. Development of a New Method for the Analysis of Cannabinoids in Honey by Means of High-Performance Liquid Chromatography Coupled with Electrospray Ionisation-Tandem Mass Spectrometry Detection. *J. Chromatogr. A* **2019**, *1597*, 179–186, doi:10.1016/j.chroma.2019.03.034.
115. Di Marco Pisciottano, I.; Guadagnuolo, G.; Soprano, V.; Esposito, M.; Gallo, P. A Survey of Δ9-THC and Relevant Cannabinoids in Products from the Italian Market: A Study by LC–MS/MS of Food, Beverages and Feed. *Food Chem.* **2021**, *346*, 128898, doi:10.1016/j.foodchem.2020.128898.

116. Citti, C.; Ciccarella, G.; Braghiroli, D.; Parenti, C.; Vandelli, M.A.; Cannazza, G. Medicinal Cannabis: Principal Cannabinoids Concentration and Their Stability Evaluated by a High Performance Liquid Chromatography Coupled to Diode Array and Quadrupole Time of Flight Mass Spectrometry Method. *J. Pharm. Biomed. Anal.* **2016**, *128*, 201–209, doi:10.1016/j.jpba.2016.05.033.
117. Ambach, L.; Penitschka, F.; Broillet, A.; König, S.; Weinmann, W.; Bernhard, W. Simultaneous Quantification of Delta-9-THC, THC-Acid A, CBN and CBD in Seized Drugs Using HPLC-DAD. *Forensic Sci. Int.* **2014**, *243*, 107–111, doi:10.1016/j.forsciint.2014.06.008.
118. Wakshlag, J.J.; Cital, S.; Eaton, S.J.; Prussin, R.; Hudalla, C. Cannabinoid, Terpene, and Heavy Metal Analysis of 29 Over-the-Counter Commercial Veterinary Hemp Supplements. *Vet. Med. Res. Rep.* **2020**, *Volume 11*, 45–55, doi:10.2147/VMRR.S248712.
119. Pacifici, R.; Marchei, E.; Salvatore, F.; Guandalini, L.; Busardò, F.P.; Pichini, S. Evaluation of Cannabinoids Concentration and Stability in Standardized Preparations of Cannabis Tea and Cannabis Oil by Ultra-High Performance Liquid Chromatography Tandem Mass Spectrometry. *Clin. Chem. Lab. Med. CCLM* **2017**, *55*, doi:10.1515/cclm-2016-1060.
120. Gul, W.; Gul, S.W.; Radwan, M.M.; Wanas, A.S.; Mehmedic, Z.; Khan, I.I.; Sharaf, M.H.M.; ElSohly, M.A. Determination of 11 Cannabinoids in Biomass and Extracts of Different Varieties of Cannabis Using High-Performance Liquid Chromatography. *J. AOAC Int.* **2015**, *98*, 1523–1528, doi:10.5740/jaoacint.15-095.
121. Pavlovic, R.; Panseri, S.; Giupponi, L.; Leoni, V.; Citti, C.; Cattaneo, C.; Cavaletto, M.; Giorgi, A. Phytochemical and Ecological Analysis of Two Varieties of Hemp (Cannabis Sativa L.) Grown in a Mountain Environment of Italian Alps. *Front. Plant Sci.* **2019**, *10*, doi:10.3389/fpls.2019.01265.
122. Calvi, L; Pentimalli, D.; Panseri, S.; Giupponi, L.; Gelmini, F.; Beretta, G.; Vitali, D.; Bruno, M.; Zilio, E.; Pavlovic, R.; et al. Comprehensive Quality Evaluation of Medical Cannabis Sativa L. Inflorescence and Macerated Oils Based on HS-SPME Coupled to GC-MS and LC-HRMS (q-Exactive Orbitrap®) Approach. *J. Pharm. Biomed. Anal.* **2018**, *150*, 208–219, doi:10.1016/j.jpba.2017.11.073.
123. Giupponi, L.; Leoni, V.; Pavlovic, R.; Giorgi, A. Influence of Altitude on Phytochemical Composition of Hemp Inflorescence: A Metabolomic Approach. *Mol. Basel Switz.* **2020**, *25*, doi:10.3390/molecules25061381.
124. Pavlovic, R.; Nenna, G.; Calvi, L.; Panseri, S.; Borgonovo, G.; Giupponi, L.; Cannazza, G.; Giorgi, A. Quality Traits of "Cannabidiol Oils": Cannabinoids Content, Terpene Fingerprint and Oxidation Stability of European Commercially Available Preparations. *Molecules* **2018**, *23*, 1230, doi:10.3390/molecules23051230.
125. Jin, D.; Dai, K.; Xie, Z.; Chen, J. Secondary Metabolites Profiled in Cannabis Inflorescences, Leaves, Stem Barks, and Roots for Medicinal Purposes. *Sci. Rep.* **2020**, *10*, 3309, doi:10.1038/s41598-020-60172-6.
126. Palermiti, A.; Cafaro, A.; Barco, S.; Bucchioni, P.; Franceschini, P.; Cusato, J.; De Nicolò, A.; Manca, A.; De Vivo, E.D.; Russo, E.; et al. Analysis of Cannabinoids Concentration in Cannabis Oil Galenic Preparations: Harmonization between Three Laboratories in Northern Italy. *Pharmaceuticals* **2021**, *14*, 462, doi:10.3390/ph14050462.
127. Scheunemann, A.; Elsner, K.; Germerott, T.; Hess, C.; Zörntlein, S.; Röhrich, J. Extensive Phytocannabinoid Profiles of Seized Cannabis and Cannabis-Based Medicines – Identification of Potential Distinguishing Markers. *Forensic Sci. Int.* **2021**, *322*, 110773, doi:10.1016/j.forsciint.2021.110773.
128. Nuapia, Y.; Maraba, K.; Tutu, H.; Chimuka, L.; Cukrowska, E. In Situ Decarboxylation-Pressurized Hot Water Extraction for Selective Extraction of Cannabinoids from Cannabis Sativa. Chemometric Approach. *Molecules* **2021**, *26*, 3343, doi:10.3390/molecules26113343.
129. Pexová Kalinová, J.; Vrchotová, N.; Tříska, J.; Hellerová, Š. Industrial Hemp (Cannabis Sativa L.) as a Possible Source of Cannabidiol. *J. Cent. Eur. Agric.* **2021**, *22*, 110–118, doi:10.5513/JCEA01/22.1.2860.
130. Deidda, R.; Schelling, C.; Roussel, J.-M.; Dispas, A.; Bleye, C.D.; Ziemons, É.; Hubert, P.; Veuthey, J.-L. The Analysis of Cannabinoids in Cannabis Samples by Supercritical Fluid Chromatography and Ultra-High-Performance Liquid Chromatography: A Comparison Study. *Anal. Sci. Adv.* **2021**, *2*, 2–14, doi:10.1002/ansa.202000091.
131. Yangsud, J.; Santasanasuwan, S.; Ahkkarachinoreh, P.; Maha, A.; Madaka, F.; Suksaeree, J.; Songsak, T.; Vutthipong, A.; Monton, C. Stability of Cannabidiol, Δ9-Tetrahydrocannabinol, and Cannabinol under Stress Conditions. *Adv. Tradit. Med.* **2021**, doi:10.1007/s13596-021-00590-7.
132. Zaripov, E.A.; Lee, T.; Dou, Y.; Harris, C.S.; Egorov, A.; Berezovski, M.V. Single-Run Separation and Quantification of 14 Cannabinoids Using Capillary Electrophoresis. *Separations* **2021**, *8*, 30, doi:10.3390/separations8030030.

133. Knezevic, F.; Nikolai, A.; Marchart, R.; Sosa, S.; Tubaro, A.; Novak, J. Residues of Herbal Hemp Leaf Teas – How Much of the Cannabinoids Remain? *Food Control* **2021**, *127*, 108146, doi:10.1016/j.foodcont.2021.108146.
134. Galettis, P.; Williams, M.; Gordon, R.; Martin, J.H. A Simple Isocratic HPLC Method for the Quantitation of 17 Cannabinoids. *Aust. J. Chem.* **2021**, *74*, 453, doi:10.1071/CH20380.
135. Saloner, A.; Bernstein, N. Nitrogen Supply Affects Cannabinoid and Terpenoid Profile in Medical Cannabis (Cannabis Sativa L.). *Ind. Crops Prod.* **2021**, *167*, 113516, doi:10.1016/j.indcrop.2021.113516.
136. Piccolella, S.; Formato, M.; Pecoraro, M.T.; Crescente, G.; Pacifico, S. Discrimination of CBD-, THC- and CBC-Type Acid Cannabinoids through Diagnostic Ions by UHPLC-HR-MS/MS in Negative Ion Mode. *J. Pharm. Biomed. Anal.* **2021**, *201*, 114125, doi:10.1016/j.jpba.2021.114125.
137. Schettino, L.; Prieto, M.; Benedé, J.L.; Chisvert, A.; Salvador, A. A Rapid and Sensitive Method for the Determination of Cannabidiol in Cosmetic Products by Liquid Chromatography–Tandem Mass Spectrometry. *Cosmetics* **2021**, *8*, 30, doi:10.3390/cosmetics8020030.
138. Christinat, N.; Savoy, M.-C.; Mottier, P. Development, Validation and Application of a LC-MS/MS Method for Quantification of 15 Cannabinoids in Food. *Food Chem.* **2020**, *318*, 126469, doi:10.1016/j.foodchem.2020.126469.
139. Reyes-Garcés, N.; Myers, C. Analysis of the California List of Pesticides, Mycotoxins, and Cannabinoids in Chocolate Using Liquid Chromatography and Low-pressure Gas Chromatography-based Platforms. *J. Sep. Sci.* **2021**, *44*, 2564–2576, doi:10.1002/jssc.202001265.
140. Tremlová, B.; Mikulášková, H.K.; Hajduchová, K.; Jancikova, S.; Kaczorová, D.; Ćavar Zeljković, S.; Dordevic, D. Influence of Technological Maturity on the Secondary Metabolites of Hemp Concentrate (Cannabis Sativa L.). *Foods* **2021**, *10*, 1418, doi:10.3390/foods10061418.
141. Sánchez-Carnerero Callado, C.; Núñez-Sánchez, N.; Casano, S.; Ferreiro-Vera, C. The Potential of near Infrared Spectroscopy to Estimate the Content of Cannabinoids in Cannabis Sativa L.: A Comparative Study. *Talanta* **2018**, *190*, 147–157, doi:10.1016/j.talanta.2018.07.085.
142. Espel Grekopoulos, J. Construction and Validation of Quantification Methods for Determining the Cannabidiol Content in Liquid Pharma-Grade Formulations by Means of Near-Infrared Spectroscopy and Partial Least Squares Regression. *Med. Cannabis Cannabinoids* **2019**, *2*, 43–55, doi:10.1159/000500266.
143. Borille, B.T.; Marcelo, M.C.A.; Ortiz, R.S.; Mariotti, K. de C.; Ferrão, M.F.; Limberger, R.P. Near Infrared Spectroscopy Combined with Chemometrics for Growth Stage Classification of Cannabis Cultivated in a Greenhouse from Seized Seeds. *Spectrochim. Acta. A. Mol. Biomol. Spectrosc.* **2017**, *173*, 318–323, doi:10.1016/j.saa.2016.09.040.
144. Duchateau, C.; Kauffmann, J.; Canfyn, M.; Stévigny, C.; De Braekeleer, K.; Deconinck, E. Discrimination of Legal and Illegal Cannabis Spp. According to European Legislation Using near Infrared Spectroscopy and Chemometrics. *Drug Test. Anal.* **2020**, *12*, 1309–1319, doi:10.1002/dta.2865.
145. Risoluti, R.; Gullifa, G.; Battistini, A.; Materazzi, S. Detection of Cannabinoids in Veterinary Feeds by MicroNIR/Chemometrics: A New Analytical Platform. *The Analyst* **2020**, *145*, 1777–1782, doi:DOI: 10.1039/C9AN01854A.
146. Deidda, R.; Coppey, F.; Damergi, D.; Schelling, C.; Coïc, L.; Veuthey, J.-L.; Sacré, P.-Y.; De Bleye, C.; Hubert, P.; Esseiva, P.; et al. New Perspective for the In-Field Analysis of Cannabis Samples Using Handheld near-Infrared Spectroscopy: A Case Study Focusing on the Determination of Δ9-Tetrahydrocannabinol. *J. Pharm. Biomed. Anal.* **2021**, *202*, 114150, doi:10.1016/j.jpba.2021.114150.
147. Mendez, J.; Francisco, S. Optimization of Cannabis Grows Using Introduction Fourier Transform Mid-Cannabis Use Is Becoming Increasingly Legal in the United States. One of The. 4.
148. Geskovski, N.; Stefkov, G.; Gigopulu, O.; Stefov, S.; Huck, C.W.; Makreski, P. Mid-Infrared Spectroscopy as Process Analytical Technology Tool for Estimation of THC and CBD Content in Cannabis Flowers and Extracts. *Spectrochim. Acta. A. Mol. Biomol. Spectrosc.* **2021**, *251*, 119422, doi:10.1016/j.saa.2020.119422.
149. Sanchez, L.; Filter, C.; Baltensperger, D.; Kurouski, D. Confirmatory Non-Invasive and Non-Destructive Differentiation between Hemp and Cannabis Using a Hand-Held Raman Spectrometer. *RSC Adv.* **2020**, *10*, 3212–3216, doi:10.1039/C9RA08225E.
150. Sanchez, L.; Baltensperger, D.; Kurouski, D. Raman-Based Differentiation of Hemp, Cannabidiol-Rich Hemp, and Cannabis. *Anal. Chem.* **2020**, *92*, 7733–7737, doi:10.1021/acs.analchem.0c00828.
